# Efficient Red Light–Driven Singlet Oxygen Photocatalysis with an Osmium‐Based Coulombic Dyad

**DOI:** 10.1002/anie.202502840

**Published:** 2025-07-15

**Authors:** Matthias Schmitz, Robert Naumann, Katja Heinze, Christoph Kerzig

**Affiliations:** ^1^ Department of Chemistry Johannes Gutenberg University Mainz Duesbergweg 10–14 55128 Mainz Germany

**Keywords:** Coulombic dyad, Energy transfer, Photocatalysis, Sustainable chemistry, Time‐resolved spectroscopy

## Abstract

Photoactive osmium complexes are widely used sensitizers for the generation of singlet oxygen because they can be excited directly into their triplet states with low‐energy red light. However, their short‐lived excited states reduce quenching efficiencies and reaction quantum yields significantly. To elongate the excited state lifetime, osmium complexes have been linked to organic chromophores to form molecular dyads. This approach, although effective, is time‐ and resource‐consuming, hampering larger‐scale applications. Here, we demonstrate a straightforward approach by directly mixing a readily available cationic osmium complex and an anionic perylene derivative in solution. Strong Coulombic interactions facilitate rapid energy transfer (∼100 ps) from the excited osmium complex to the perylene derivative, mimicking a dyad‐like system. Detailed spectroscopic investigations revealed an increased singlet oxygen formation rate by over one order of magnitude at sub‐millimolar perylene concentrations, attributed to i) the three orders of magnitude longer lifetime of the perylene triplet state produced via intra‐ion‐pair energy transfer and ii) an inherently high singlet oxygen quantum yield of that key species. The novel catalyst system enables highly productive photooxygenations in water and in a MeOH/H_2_O 10:1 mixture, highlighting the broad applicability and versatility of the Coulombic dyad approach for photocatalytic synthesis and wastewater treatment.

## Graphical abstract

Red light‐absorbing bichromophores with straightforward preparation and superior photocatalytic activity were developed. Spectroscopic studies provided deep insights into the intra‐ion‐pair energy transfer key step and the novel approach.

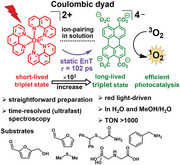

## Introduction

The photochemical generation of singlet oxygen (^1^O_2_) is of major relevance in both synthesis and photodynamic therapy (PDT).^[^ ^1^, ^2^, ^3^, ^4^, ^5^, ^6^ ^]^ In both fields, osmium complexes have gained a lot of interest as they can be excited directly into the triplet metal‐to‐ligand charge transfer ^3^MLCT state with low‐energy red or near‐infrared (NIR) light due to their high spin orbit coupling (SOC), which reduces energy loss upon photon absorption as the higher‐energy singlet state is not populated first.^[^ ^7^, ^8^, ^9^ ^]^ The triplet state can subsequently form ^1^O_2_ upon the collision with molecular oxygen in solution.^[^ ^10^, ^11^, ^12^, ^13^, ^14^ ^]^ This makes applications

1. particularly mild due to the use of low‐energy light, which reduces photodamage and competing absorption by substrates,
2. energy‐efficient as only moderately more than the required energy of ∼1 eV is used for the sensitization of ^1^O_2_ per photon, and
3. very effective for applications on larger scale as red light has a high penetration depth.^[^ ^15^, ^16^, ^17^, ^18^, ^19^, ^20^, ^21^, ^22^, ^23^ ^]^

**Figure 1:**
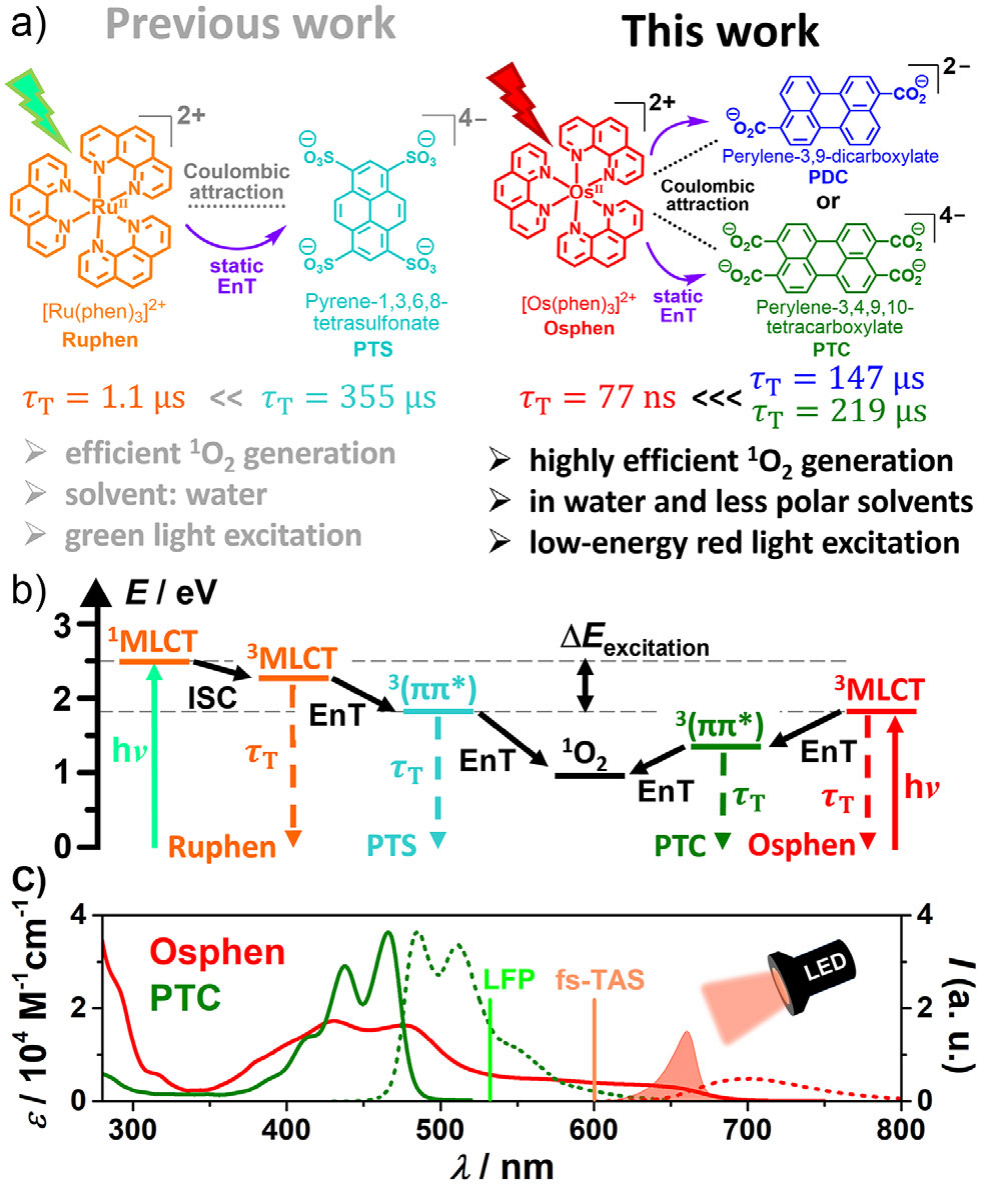
a) Comparison of the key properties and applicability of a previously described and the novel Coulombic dyad photocatalysts presented herein. b) Energetic scheme for the formation of ^1^O_2_ with both dyads. c) Stationary absorption (solid lines) and emission spectra (dotted lines) of the key components of the best‐performing Os‐based Coulombic dyad as well as an indication of the excitation wavelengths used for time‐resolved spectroscopy (vertical line) and lab‐scale irradiation experiments (filled red curve).

Furthermore, this intrinsic advantage regarding the absorption properties of osmium complexes, which bypasses energy loss through intersystem crossing (ISC), is used in photon upconversion to yield high anti‐Stokes shifts.^[^ ^24^, ^25^, ^26^, ^27^, ^28^ ^]^ However, osmium complexes have a decisive disadvantage: Due to the high SOC and the small energy gap between the ^3^MLCT state and the ground state, conventional osmium complexes have short excited state lifetimes,^[^ ^8^, ^29^, ^30^ ^]^ which leads to intrinsically very low quantum yields in diffusion‐controlled processes,^[^ ^8^ ^]^ such as ^1^O_2_ generation in solution. This kinetic issue may cancel out the aforementioned advantages of using low‐energy red light. The challenge of the short excited state lifetime of a metal complex can be approached by a covalent linkage with a purely organic chromophore, whose energetically lower, comparatively much longer‐lived ^3^(ππ*) state is rapidly and quantitatively populated via an intramolecular Dexter energy transfer.^[^ ^31^, ^32^, ^33^, ^34^, ^35^, ^36^, ^37^, ^38^, ^39^, ^40^ ^]^ The longer‐lived triplet states of osmium‐based dyads have also been successfully used for more efficient photon upconversion and PDT.^[^ ^10^, ^11^, ^12^, ^13^, ^28^, ^37^, ^41^, ^42^, ^43^, ^44^, ^45^ ^]^ However, this method includes a multistep and usually nonquantitative synthesis of the bichromophore, which is less desirable given the high cost of osmium. Recently, we have shown that the photophysical properties and kinetic advantages in bimolecular reactions of a molecular dyad are essentially identical or even more advantageous by using a so‐called Coulombic dyad.^[^ ^46^ ^]^ In that study, which was stimulated by several key findings related to counter‐ion and ion‐pairing effects in photochemistry,^[^ ^47^, ^48^, ^49^, ^50^ ^]^ a simple and positively charged metal complex ([Ru(phen)_3_]^2+^, **Ruphen**, phen = 1,10‐phenanthroline) and a negatively charged organic chromophore (1,3,6,8‐pyrenetetrasulfonate, **PTS**) form an ion‐pair in water.^[^ ^46^ ^]^ This approach enabling dyad‐like character bypasses the time‐ and resource‐consuming (multistep) preparation of a molecular dyad. Instead, a Coulombic dyad is achieved by mixing the easily accessible salts of the metal complex and the organic chromophore in solution to use the precious metal complex as efficiently as possible in photocatalysis. Following our proof‐of‐concept study,^[^ ^46^ ^]^ this approach is now applied to a simple dicationic osmium complex and readily available anionic perylene derivatives. Furthermore, its applicability is extended to i) red light–driven photocatalysis, which is of great current interest,^[^ ^15^, ^51^, ^52^, ^53^, ^54^, ^55^ ^]^ and ii) a less polar solvent mixture to enable broader applications (see Figure 1). For this purpose, we present a thorough investigation of the new coulombic dyad with both a dianionic and a tetra‐anionic organic counterpart by laser flash photolysis (LFP) and fs transient absorption spectroscopy (fs‐TAS), a reliable assay to confirm the highly efficient ^1^O_2_ generation and several laboratory‐scale irradiation experiments.

## Results and Discussion

### Coulombic Dyad Design

The easily accessible and water‐soluble chloride salt of osmium tris‐1,10‐phenanthroline [Os(phen)_3_]^2+^ (**Osphen**) served as the red light–absorbing metal complex for the novel Coulombic dyad system. The PF_6_‐salt was synthesized in a single step according to a modified procedure by Constable et al.^[^ ^56^ ^]^ with a yield of 86%, which was quantitatively converted to the chloride salt with an ion exchange resin (see Chapter S2 for details). The UV–vis spectrum in water (see Figure 1c) shows a broad absorption band for the direct transition into the ^3^MLCT state, which shows an absorption onset at ∼710 nm.^[^ ^30^ ^]^ For **^3^Osphen** with a triplet energy, *E*_T_, of 1.80 eV,^[^ ^41^ ^]^ perylene with an *E*_T_ of ∼1.5 eV^[^ ^57^ ^]^ is a suitable energy acceptor, which was already demonstrated in several molecular dyads.^[^ ^37^, ^41^, ^42^, ^58^ ^]^ To enable strong Coulombic interactions with **Osphen**, the water‐soluble potassium salt of the tetra‐anion perylenetetracarboxylate (**PTC**) was used, which was synthesized in one step by the hydrolysis of the commercially available dianhydride in 93% yield (see Chapter S2 for details). The extension of the π system through the carboxylate groups is expected to reduce *E*_T_, which is estimated to be ∼1.30 eV according to DFT calculations (see Chapter S1.9). Thus, the *E*_T_ of **PTC** is ∼0.5 eV below that of the sensitizer, which paves the way for a fast intermolecular energy transfer under formation of **^3^PTC** and practically excludes intermolecular back‐energy transfer,^[^ ^59^ ^]^ forming a localized triplet rather than an excited state equilibrium with triplet reservoir.^[^ ^31^, ^34^ ^]^ The UV–vis absorption spectrum of **PTC** shows a similar resolution of the vibronic transitions of the first electronically excited state as perylene (**Per**) (see Chapter S3). The lowest‐energy absorption band peaking at 466 nm is redshifted by about 30 nm compared to **Per** due to the π system‐extending substituents. A pH‐dependent UV–vis measurement in water indicates that **PTC** is present in its fully deprotonated state at a pH of 11. This maximizes the Coulombic interactions between **PTC** and **Osphen** at a minimal concentration of NaOH (1 mM). A ^1^H NMR experiment at mM concentrations confirms a ground state 1:1 association between **Osphen** and **PTC** in solution with an association constant of *K*_11_ = (1.9 ± 0.4)⋅10^4^ M^−1^ (see Chapter S4), which lays the grounds for both chromophores to act as dyad system.

### Stationary and Time‐Resolved (LFP) Investigations of Initial Energy Transfer Step

**Figure 2:**
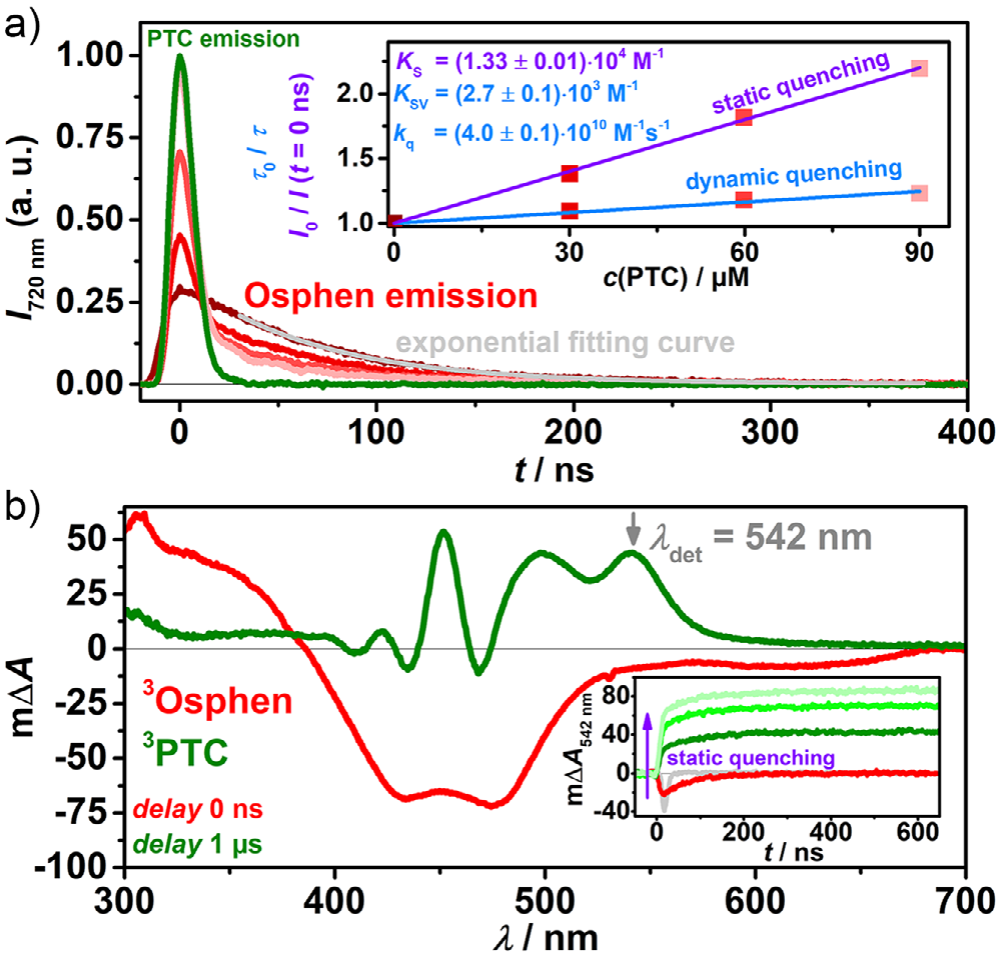
a) (Main plot) time‐resolved emission of an aqueous solution of **Osphen** (*c*(**Osphen**) = 16 µM) with different *c*(**PTC**) (color‐coded red lines) or a solution containing *c*(**PTC**) = 90 µM (green) after laser excitation (*λ*_exc_ = 532 nm); inset: corresponding Stern–Volmer plot. b) (Main plot) transient absorption spectra of a solution containing *c*(**Osphen**) = 16 µM without (red) or with *c*(**PTC**) = 30 µM (green) after laser excitation (*λ*_exc_ = 532 nm); inset: time‐resolved absorption at 542 nm of the same solutions shown above. The solution containing only *c*(**PTC**) = 90 µM is colored in gray.

The quenching of **^3^Osphen** by **PTC** was first investigated by recording steady‐state emission spectra of the excited osmium complex (see Chapter S3). A clear quenching of the **^3^Osphen** phosphorescence could be observed even at **PTC** concentrations as low as 30 µM. In order to quantify the contributions of static and/or dynamic quenching, the phosphorescence was recorded in a time‐resolved manner (see Figure 2a, main plot).^[^ ^60^ ^]^ The unquenched lifetime of **^3^Osphen** in water was determined to be ∼77 ns, which is in line with the literature value.^[^ ^30^ ^]^ In the presence of the quencher **PTC**, an additional emission signal can be observed after 532 nm laser excitation with a laser‐limited lifetime. Similar observations were made in steady‐state measurements and this can be attributed to the emission of **PTC** or its aggregates that may form in aqueous solution.^[^ ^61^ ^]^ **PTC** is essentially transparent at the excitation wavelength of 532 nm. However, based on a recent study,^[^ ^61^ ^]^ **PTC** forms dimers at higher concentrations (>1 mM) that weakly absorb at 532 nm and emit at the detection wavelength of the experiment displayed in Figure 2a. The very small fraction of **PTC** dimers at our concentrations (*c* < 200 µM) does not negatively affect the performance of our Coulombic dyad but its emission is still sufficient to initially mask the very weak phosphorescence of **^3^Osphen**. The filter effect due to **PTC** is negligible (see Chapter S3). After the decay of the **PTC** (dimer) fluorescence, the monoexponential decay of the **^3^Osphen** phosphorescence becomes visible. An exponential fit of the pure **^3^Osphen** emission was used on the one hand to determine the lifetime and thus the rate of dynamic quenching and on the other hand to extrapolate the initial amplitude of the emission (*t* = 0 ns in Figure 2a, main plot), which is important for the quantification of the static quenching process.^[^ ^46^ ^]^ An association constant between **Osphen** and **PTC** in solution of *K*_s_ = (1.33 ± 0.01)⋅10^4^ M^−1^ was determined by a Stern–Volmer analysis based on the decrease of the initial emission intensity (see Figure 2a, inset), which agrees with the results of the ^1^H NMR titration experiment, taking the different concentration ranges into account. This value is on the same order of magnitude as for a similar Coulombic dyad, which is composed of the dicationic **Ruphen** and the tetra‐anionic **PTS**.^[^ ^46^ ^]^ A Stern–Volmer analysis of the dynamic quenching process revealed a quenching rate constant of *k*_q_ = (4.0 ± 0.1)⋅10^10^ M^−1^s^−1^, which is well above the conventional diffusion limit (between uncharged species) in water (6.5⋅10^9^ M^−1^s^−1^)^[^ ^8^ ^]^ and which is similar to what has been observed in the **Ruphen**−**PTS** system.^[^ ^46^ ^]^

Despite the very high value for *k*_q_, less than 9% of the triplet‐excited Os complexes are dynamically quenched by **PTC** at *c*(**PTC**) = 90 µM due to the very short lifetime of **^3^Osphen**. In contrast, static quenching, which is virtually independent of the excited state lifetime of the sensitizer, is the dominant quenching pathway with an efficiency as high as 54% due to preorganization via ion‐pairing at the same concentration of **PTC**. These results imply that a significantly reduced aggregation constant leads to a drastic reduction in the quenching efficiency. This was demonstrated by utilizing reference systems with the dianionic perylenedicarboxylate (**PDC**) in water and the neutral **Per** in acetonitrile as quenchers (see Chapter S6). When using dianionic **PDC**, both the values for *K*_s_ and the static quenching efficiency are halved compared to tetra‐anionic **PTC**, which we attribute to less pronounced Coulombic attraction for the dianionic organic chromophore. With uncharged **Per** as a **^3^Osphen** quencher, only dynamic quenching occurs. Consequently, a very low quenching efficiency is observed under our conditions (see Figure S17). All these findings clearly show that static quenching is a result of the strong Coulombic interactions between the two chromophores. **^3^PTC** shows a similar shape in the TA spectrum compared to **^3^Per** in acetonitrile (compare Figure 2b and Chapter S6). The longest‐wavelength absorption maximum at 542 nm is redshifted by ∼60 nm. The occurrence of static quenching can also be verified by measuring kinetic absorption traces at this wavelength that is indicative of **^3^PTC**. Within the pulse length of the laser, a rapid increase in transient absorption can be observed at 542 nm, indicating a sub‐ns energy transfer from **^3^Osphen** to **PTC**. This static signal rise is followed by a more gradual increase due to the dynamic quenching process. As perylenes usually have a high fluorescence quantum yield and a very low ISC quantum yield,^[^ ^62^ ^]^ practically no **^3^PTC** is formed by direct excitation in the absence of **Osphen**, which was confirmed in a control experiment (see Figure 2b, inset). The artifacts due to the emission of **PTC** prevent further meaningful analysis of static and dynamic quenching via time‐resolved emission at higher **PTC** concentrations for experimental reasons. Nevertheless, in order to estimate the quenching efficiency at higher concentrations, the value of Δ*A* of the resulting **^3^PTC** was recorded in the time‐resolved absorption, which is proportional to the quenching efficiency. This results in an energy transfer quenching efficiency of ∼88% for **^3^Osphen** by **PTC** at *c*(**PTC**)  = 200 µM (see Figure S18C).

### Ultrafast Transient Absorption Spectroscopy (fs‐TAS)

The initial energy transfer populating the triplet state of the organic moiety is a crucial step within a dyad framework. The results obtained from LFP with ns resolution indicate a process on the sub‐ns time scale for static (i.e., intra‐ion‐pair) energy transfer (see Figure 2b, inset).

**Figure 3:**
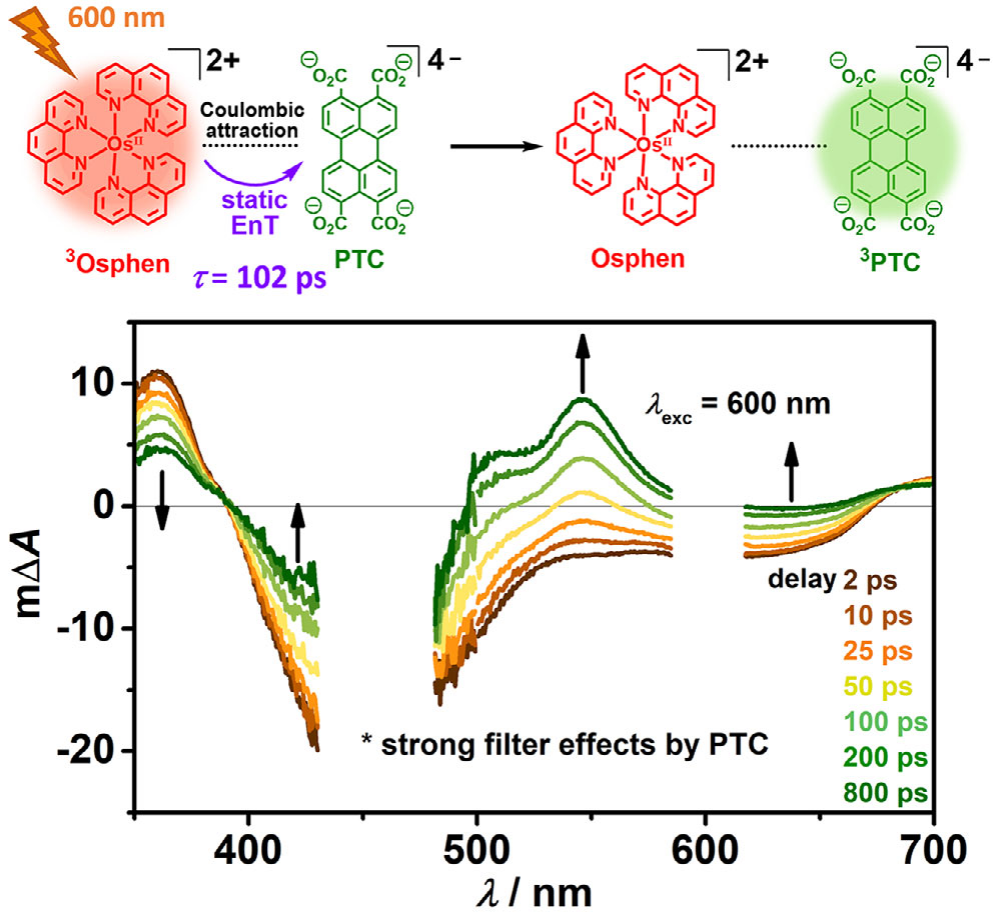
Scheme of the initial intra‐ion‐pair energy transfer step between **Osphen** and **PTC** (top). TA spectra (bottom) of a solution containing *c*(**Osphen**) = 0.65 mM and *c*(**PTC**) = 0.46 mM in an aqueous solution (1 mM NaOH) recorded at certain delay times after laser excitation (*λ*_exc_ = 600 nm, pulse length: <175 fs).

To resolve its kinetics within the **Osphen** and **PTC** ion‐pair in water, fs‐TAS was conducted (Figure 3). Under the selected concentrations, which had to be adapted for obtaining meaningful results with this more sophisticated method, about 55% of the ground state Os complex molecules exist as **Osphen**−**PTC** ion‐pair and dynamic quenching is predicted to occur with a time constant of 34 ns. These conditions allow us to observe and analyze the ultrafast static quenching in isolation. In the spectra shown in Figure 3, data points around 450 and 600 nm are omitted due to artefacts caused by strong filter effects by **PTC** or scattering of the excitation light, respectively. After selective laser excitation at 600 nm, a characteristic transient absorption spectrum of **^3^Osphen** can be observed (compare ns LFP measurements, Figure 2b, main plot), with a ground state bleach from 400–680 nm and an excited state absorption band around 360 nm. Over the course of one nanosecond, an excited state absorption band with a maximum at 542 nm is formed, which is characteristic for the presence of **^3^PTC**. Additionally, the depopulation of **^3^Osphen** is indicated by the simultaneous decrease of the excited state absorption around 360 nm and a reduction of the ground state bleach around 420 nm. The time constant of the initial energy transfer step was determined to be (102 ± 5) ps (see Chapter S5 for details). We regard this time constant as clear evidence for preorganization and a close proximity of the oppositely charged chromophores because it is 330 times faster than the diffusion‐based quenching under these conditions. Analogously, the kinetics of the energy transfer within the ion‐pair consisting of **Osphen** and the dianionic **PDC** was analyzed (see Chapter S5 for details). There, the process occurs with a time constant of 91 ps, which is slightly faster compared to the energy transfer within the **Osphen**–**PTC** pair. This step is most likely influenced by the thermodynamic driving force, the geometry of the ion‐pair, and the electronic coupling between the charged chromophores. A similarly high driving force is expected for both energy transfer steps, with a slightly higher driving force in the case of **PTC**. The energy difference to **^3^Osphen** is estimated with ∼0.39 and ∼0.50 eV for **^3^PDC** and **^3^PTC**, respectively. Conversely, it is assumed that in the case of **PDC**, the steric hindrance in the ion‐pair is lower, which enables a better overlap of the π systems of one phenanthroline ligand and the perylene core structure, resulting in a higher electronic coupling and a faster pseudo‐static energy transfer step. Interestingly, the rate of the population of the organic triplet state is comparable to the kinetics of well‐investigated molecular dyad systems based on Ru, Re, and Pt (between 4.8 and 2000 ps for the cited examples).^[^ ^33^, ^34^, ^63^, ^64^, ^65^, ^66^, ^67^ ^]^ This similarity further emphasizes that Coulomb‐bound ion‐pairs can be regarded as a novel class of bichromophores, which justifies the name Coulombic dyad.^[^ ^46^ ^]^

### Quantification of Increased ^1^O_2_ Formation Rate with a ^1^O_2_ Assay

**Figure 4:**
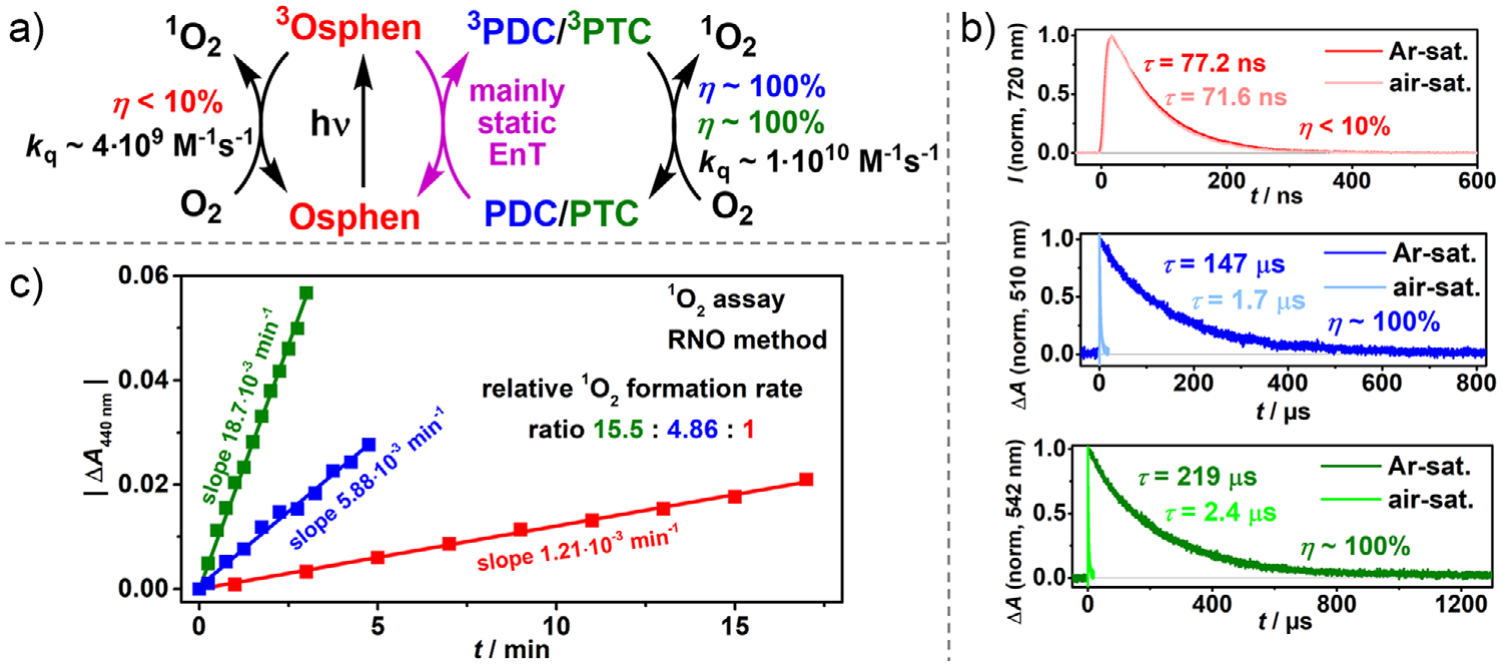
a) Reaction scheme of the less efficient formation of ^1^O_2_ without and the much more efficient formation of ^1^O_2_ with the mediators **PDC** or **PTC**. b) Time‐resolved emission of Ar‐ or air‐saturated aqueous solution containing *c*(**Osphen**) = 16 µM (red) and time‐resolved absorption of a solution containing additionally *c*(**PDC**) = 90 µM or *c*(**PTC**) = 90 µM under similar conditions after 532 nm laser excitation. c) Results of ^1^O_2_ assay displaying different ^1^O_2_ formation rates for an aqueous solution containing *c*(**Osphen**) = 15 µM without (red) or with *c*(**PDC**) = 45 µM (blue)/*c*(**PTC**) = 45 µM (green) under red light excitation (660 nm LED). See Chapter S7 for further information.

As highlighted in the introduction, osmium complexes have attracted considerable attention for singlet oxygen generation due to their beneficial ability to absorb light across the visible spectrum, particularly the red and NIR regions. Despite this advantageous spectral absorption, an inherent challenge arises from the short lifetime of **^3^Osphen** and related complexes in the low nanosecond range. Combined with a poor solubility of molecular oxygen in aqueous solution (0.27 mM at 25 °C),^[^ ^8^ ^]^ a low quantum yield for the ^1^O_2_ generation is thus expected. This is demonstrated by the very low quenching efficiency of **^3^Osphen** by dissolved oxygen of ∼0.07 in air‐saturated water, as shown in Figure 4. In contrast, **^3^PTC** generated in the Coulombic dyad is almost quantitatively quenched by molecular oxygen because its lifetime is more than three orders of magnitude longer. This lifetime‐dependent quenching efficiency difference can be displayed in a kinetic simulation (see Figure S20). To further explore the impact of the lifetime extension on the ^1^O_2_ formation rates, a comparative analysis for aqueous solutions based on the RNO method by Kraljić and Mohsni^[^ ^34^, ^68^, ^69^ ^]^ was performed (see Chapters S1.7 and S7 for details). In this method, an organic nitrosyl compound is converted by ^1^O_2_ to the corresponding nitro derivative, catalyzed by imidazole. The consumption of the nitrosyl compound can be observed via the decrease of its absorption band at 440 nm. This decrease in absorption is proportional to the rate of ^1^O_2_ formation, which means that it can be quantified relative to each other for different systems under uniform irradiation conditions. For experimental reasons, the concentrations of **Osphen** and **PTC** had to be adjusted for this method. However, based on the previous quantitative spectroscopic investigations, the efficiency of each step can be calculated. Utilizing the entire Coulombic dyad with *c*(**Osphen**) = 15 µM and *c*(**PTC**) = 45 µM, an increase by more than one order of magnitude (15.5‐fold) of the ^1^O_2_ formation rate is observed compared to the results using **Osphen** only (see Figure 4c, all photophysical key properties of the **Osphen**‐based Coulombic dyads are summarized in Table S3). According to our analysis in Figure S6D, a quenching efficiency of ∼0.40 is achieved for **^3^Osphen** by **PTC** for the used concentrations. Due to the near quantitative quenching of **^3^PTC** by molecular oxygen, the overall quenching efficiency for the reaction with molecular oxygen increases from ∼0.073 (**Osphen** only) to ∼0.40 (**Osphen** and **PTC**), which represents a 5.5‐fold increase. This discrepancy between predicted and observed ^1^O_2_ formation rate can be explained by the inherent quenching efficiency *f*_Δ_, which describes the fraction of ^1^O_2_ that is formed by the quenching process with molecular oxygen.^[^ ^1^ ^]^ The formation of the superoxide radical anion O_2_^•−^ via electron transfer represents a competing pathway to the desired energy transfer reaction. Hence, *f*_Δ_ has to be higher for **^3^PTC** compared to **^3^Osphen** by a factor of ∼2.8 (∼15.5/5.5). This observation reveals another advantage of the dyad system: The transition from mixed electron transfer/energy transfer mechanisms with ^3^MLCT states to more selective energy transfer quenching with the ^3^(ππ*) state of an organic chromophore. This is in agreement with the observations of our recent study involving a Ru‐based molecular dyad^[^ ^70^ ^]^ and other investigations on bichromophores in photooxygenations.^[^ ^71^, ^72^, ^73^ ^]^ The solvent‐dependent *f*_Δ_ value is reported to be 0.76 for **Osphen** in methanol.^[^ ^74^ ^]^ For analogous Ru complexes, this value is found to decrease in more polar solvents such as water, which favors the formation of the charged species O_2_^•−^.^[^ ^75^ ^]^

Conversely, for the organic chromophore **PTC** with electron‐withdrawing groups, minimal electron transfer to molecular oxygen can be expected for the low‐energy excited state **^3^PTC**, such that we assume *f*_Δ_ to be 1 for **^3^PTC**. Considering this assumption, *f*_Δ_ is estimated to be ∼0.35 (∼1/2.8) for **Osphen**, which corresponds closely to the results of strongly related Ru complexes in water.^[^ ^1^, ^75^ ^]^ When **PDC** is utilized, which is only about half as efficient in **^3^Osphen** quenching compared to **PTC** (Figure S17), a ∼5‐fold increase in the ^1^O_2_ formation rate is observed. This represents a reduction by a factor of ∼3.2 compared to the formation rate using **PTC**. The disproportionate decrease in the formation rate can be attributed to the higher significance of electron transfer with molecular oxygen, given that **^3^PDC** is slightly higher in energy than **^3^PTC**, which can decrease *f*_Δ_. The thermodynamic feasibility of photoinduced O_2_ reduction and thus the influence on *f*_Δ_ for **^3^Osphen**, **^3^PTC**, and **^3^PDC** was investigated by complementary cyclic voltammetry measurements in water (see Chapter S8). Indeed, the excited‐state oxidation potential of **^3^Osphen** in water is more negative compared to those of the perylene‐localized triplets by as much as 0.6 V. In consequence, a high driving force for the photoinduced electron transfer to yield O_2_^•−^ can only be expected for the metal complex, whereas **^3^PTC** and **^3^PDC** are more redox inert, favoring singlet oxygen formation. To underscore the impact of lifetime extension, the widely used complex [Os(bpy)_3_]^2+^ (**Osbpy**),^[^ ^76^, ^77^, ^78^ ^]^ with an even shorter excited state lifetime (*τ*_T_ ∼ 22 ns) than **Osphen**,^[^ ^30^ ^]^ was utilized as energy donor in a Coulombic dyad. The quenching efficiency of **^3^Osbpy** by molecular oxygen in air‐saturated water is as low as ∼3%, which is approximately half as high as that for **^3^Osphen** (see Figure S62). Assuming similar values for *f*_Δ_ and *K*_s_, and consequently for the static quenching efficiency of **^3^Osbpy** by **PTC**, an additional twofold increase in the ^1^O_2_ formation rate in the presence of **PTC** is expected. This would result in an overall increase in the ^1^O_2_ formation rate by a factor of ∼30 compared to the use of **Osbpy** alone. Indeed, this predicted superincrease was validated by the ^1^O_2_ assay, as detailed in Chapter S7.

### Efficient Photooxygenations in Water and a Less Polar Solvent

**Figure 5:**
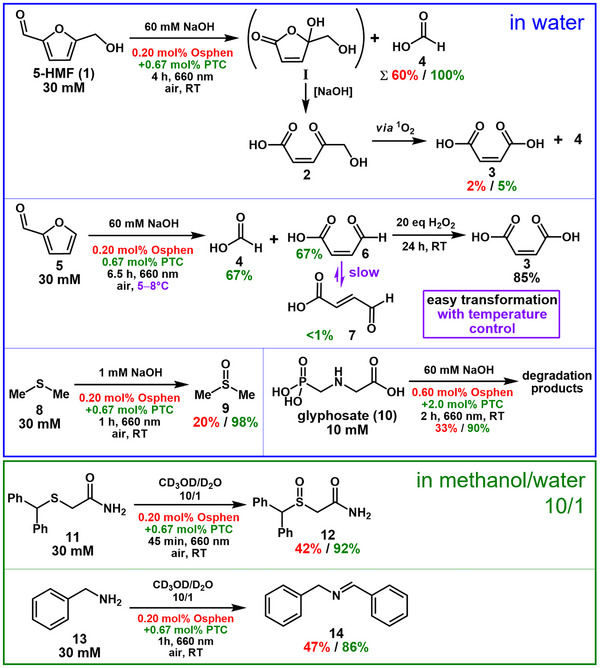
Laboratory‐scale irradiation experiments with red light (660 nm LED) confirming the improved ^1^O_2_ formation rate utilizing the novel Coulombic dyad. Yields were determined via ^1^H NMR spectroscopy. See Chapter S9 and the text for further information.

With the ^1^O_2_ assay presented in the preceding section, it could be shown that a highly efficient generation of ^1^O_2_ with the Coulombic dyad is possible due to the increase in lifetime and an improved value for *f*_Δ_. This is now used for performing efficient red light–driven photooxygenations in air‐saturated water (see Figure 5).

First, we turned to photooxygenations of 5‐HMF (**1**), which is among the promising platform chemicals from a renewable source,^[^ ^79^ ^]^ using the Coulombic dyad consisting of **Osphen** and **PTC**. After a short time, however, a red precipitate formed and the typical greenish color in the presence of dissolved **PTC** disappeared. This can be explained by the pH‐lowering effect caused by the co‐product formic acid (**4**)^[^ ^81^ ^]^ and the fact that (fully) protonated **PTC** is poorly soluble in water, which was observed in pH‐dependent UV–vis spectra (see also Chapter S3). In order to avoid the precipitation of **PTC**, two equivalents of NaOH had to be used. Two equivalents are necessary to neutralize **4** and the carboxylic acid **2**, which is formed under alkaline conditions by ring‐opening of the lactone I.^[^ ^34^ ^]^ The metastable **2** is a potential biopolymer precursor and it can be accumulated at reduced temperatures.^[^ ^34^, ^80^, ^81^ ^]^ In addition, a small amount of maleic acid was identified, which is formed as a secondary product from the oxidation of **2** alongside a further equivalent of **4**.^[^ ^34^ ^]^ The basic conditions hardly alter the quenching efficiencies of **^3^Osphen** or **^3^PTC** by molecular oxygen (see Figure S61). Control experiments rule out alternative reaction pathways (see Table S5). Utilizing the entire Coulombic dyad, the reaction is nearly complete after 4 h of irradiation with a 660 nm LED (see Figure S26). When comparing the reaction rates in terms of the initial slopes with and without **PTC**, a significant improvement by a factor of ∼3 is observed. Despite the considerable increase in the reaction rate, an even greater improvement can be expected based on the spectroscopic findings and the outcome of the ^1^O_2_ assay.

One explanation for this is the increased ionic strength due to the use of 60 mM NaOH, which suppresses the efficient association of the oppositely charged chromophores and thus the quenching efficiency of **^3^Osphen** by **PTC**.^[^ ^46^, ^82^ ^]^ In complementary LFP measurements, it was found that the quenching efficiency drops from 0.88 in 1 mM NaOH to 0.35 in 60 mM NaOH (see Figure S18). Taking the results of the ^1^O_2_ assay and the ionic strengths effects into account, the reaction rate should nevertheless be even higher than observed in the presence of **PTC**. Another explanation is the photostability of the catalyst system under long‐term irradiation. A UV–vis absorption study of the reaction solution indicates that **PTC** also reacts with ^1^O_2_ during prolonged irradiation times, which presumably deactivates the organic chromophore (see Chapter S9.1). However, what could initially be regarded as a weakness turns out to be a strength of the novel catalytic system. If perylene would be covalently linked to **Osphen**, the resulting molecular dyad would be irreversibly deactivated after the decomposition initiated by ^1^O_2_. In the case of the Coulombic dyad, only inexpensive **PTC** is decomposed and **Osphen**, which is several orders of magnitude more expensive, remains practically unchanged during the reaction (see Chapter S9.1) and could potentially be recovered after the irradiation reaction. Moreover, the turnover frequency and the photoreactivity of an irradiated photocatalysis solution can be significantly increased again upon the addition of further **PTC** (see Figure S32), emphasizing the stability of **Osphen** and the simplicity of the Coulombic dyad concept. In another approach, the probability of the reaction between **PTC** and ^1^O_2_ can initially be reduced by increasing the concentration of the substrate. By this, a considerable TON relative to the sensitizer of over 1000 was achieved after 7.5 h of irradiation (see Chapter S9.1). For the reactions in which **Osbpy** served as the sensitizer, the difference in reaction rates with and without **PTC** are more pronounced and the reaction rate when using **Osphen** and **PDC** is in‐between the reaction rates with **PTC** and without any organic chromophore (see Chapter S9.1). These findings are consistent with the results of the ^1^O_2_ assay. Furthermore, the photooxygenation of **1** was conducted using the well‐established organic photocatalyst methylene blue (MB), which is known for singlet oxygen generation in aqueous solution operating under red light excitation.^[^ ^83^, ^84^, ^85^, ^86^ ^]^ Under the same reaction conditions, however, MB shows a poor conversion below 10% after 4 h of irradiation, showing no reaction progress after the first 30 min of irradiation (see Chapter S9.1). This can be explained by the limited photostability of the organic chromophore,^[^ ^46^, ^84^, ^87^ ^]^ which can be clearly recognized by the solution turning colorless.

The observation that maleic acid occurs as a minor secondary product stimulated the endeavor to produce this industrially important chemical as a main product from renewable raw materials. Without a hydroxymethyl group, the platform chemical furfural (**5**)^[^ ^79^ ^]^ is present, which yields formic acid and maleic semialdehyde (**6**) after photooxygenation via ^1^O_2_ and a pH value above 4.^[^ ^88^, ^89^ ^]^ It was observed that **6** thermally isomerizes to the *E* isomer **7**, which can be suppressed by ice cooling (see Chapter S9.2). As we found, the semialdehyde **6** could subsequently be oxidized very efficiently with hydrogen peroxide at room temperature to maleic acid. This represents a very simple and efficient one‐pot synthesis, which was previously only possible with more complex catalytic systems, high temperatures, or by utilizing fossil fuels.^[^ ^88^, ^90^, ^91^, ^92^, ^93^, ^94^, ^95^ ^]^

As an additional photooxygenation in water, a thioether was converted to the corresponding sulfoxide, which is in general an important transformation in organic synthesis.^[^ ^96^ ^]^ The photooxygenation of dimethyl sulfide (**8**) produces DMSO (**9**) selectively under the chosen conditions (see Chapter S9.3). To our delight, the reaction is almost complete after only 1 h when the Coulombic dyad system is used, whereas the conversion of 5‐HMF requires 4 h. This is due to i) the need for only 0.5 equivalents of ^1^O_2_ for the photooxygenation of thioethers^[^ ^97^ ^]^ instead of 1 equivalent for 5‐HMF and ii) the low NaOH concentrations, which allow a minimum ionic strength, thereby fully utilizing the potential of the Coulombic dyad. The latter is also reflected by the more pronounced improvement of the reaction rate in the presence of **PTC** by as much as a factor of ∼5.

^1^O_2_ is not only a useful reagent in synthesis but also plays an important role in the degradation of environmental pollutants.^[^ ^98^, ^99^, ^100^ ^]^ Glyphosate (**10**) is the most widely used herbicide^[^ ^101^ ^]^ and its massive use leads to concerning amounts in soil, surface water, and ground water.^[^ ^102^, ^103^ ^]^ It is known to be toxic to aquatic organisms^[^ ^104^, ^105^ ^]^ and probably carcinogenic to humans,^[^ ^106^ ^]^ which raised much interest for the degradation of this pollutant.^[^ ^107^ ^]^ Under alkaline conditions, **10** can be decomposed by ^1^O_2_.^[^ ^108^ ^]^ Glyphosate is known to have several protonation stages^[^ ^109^, ^110^ ^]^ and the decomposition can release acidic compounds^[^ ^111^ ^]^ such that a base excess is needed. The photocatalytic degradation of 10 mM of **10** was carried out with the same NaOH and catalyst concentrations used for the photooxygenation of 5‐HMF. Utilizing the Coulombic dyad system **Osphen**–**PTC**, 90% of **10** is decomposed after 2 h of irradiation according to ^1^H NMR (see Chapter S9.4). The downfield shift of the chemical shifts of the protic glyphosate in ^1^H NMR spectroscopy over the course of the reaction already indicates the formation of acidic degradation products. The signal that emerges at ∼2.5 ppm in the ^31^P NMR spectra can be assigned to orthophosphate.^[^ ^112^ ^]^ This, and the absence of meaningful signals in ^1^H NMR after the irradiation, implies a multistep oxidation of **10** to smaller building blocks like methylamine, ammonia, nitroxides, formaldehyde, or oxalic acid,^[^ ^111^ ^]^ which are either invisible in ^1^H NMR spectroscopy or removed from the solution by the constant air flow. Importantly, the intermediate aminomethylphosphonic acid, which is also toxic^[^ ^102^ ^]^ and which can be observed by the signal occurring ∼3 ppm downfield shifted compared to **10** in ^31^P NMR,^[^ ^112^ ^]^ is practically not present after 2 h of irradiation. Without **PTC**, the decomposition progresses much more slowly, such that only 33% is decomposed after 2 h of irradiation—in perfect agreement with the lower ^1^O_2_ formation rate in the presence of only **Osphen**.

A major difficulty in using water as the solvent is that a large number of industrially or pharmaceutically important substances or their precursors are practically insoluble in water. One well‐established approach to overcome solubility issues in aqueous solutions is to use cyclodextrins as solubilizers,^[^ ^113^ ^]^ which was also demonstrated for the first Coulombic dyad.^[^ ^46^ ^]^ In the present study, the Coulombic dyad was used in a different solvent environment, which was exploited for the synthesis of the drug modafinil (**12**, see Figure 5 for its structure).^[^ ^96^ ^]^ Modafinil is formed from the non‐water soluble precursor **11** via photooxygenation of the sulfur atom (see Chapter S9.5). As we found, all components are soluble in a less polar solvent mixture containing methanol/water 10:1 (v/v). For this solvent mixture, a significant acceleration of the reaction through the presence of **PTC** could also be observed (92% versus 42% yield after 45 min of irradiation, see also Figure S52), which can be attributed to efficient static quenching of **^3^Osphen** by **PTC** and the significantly higher lifetime of **^3^PTC** compared to **Osphen**. This beneficial situation leads to an overall higher quenching efficiency by molecular oxygen, as demonstrated by additional LFP measurements in this solvent mixture (see Chapter S9.5.1). With the promising results in this solvent mixture in hand, ^1^O_2_ mediated oxidative dimerization of benzylamine (**13**) was carried out,^[^ ^114^ ^]^ leading to the formation of **14** in 47% yield after 1 h of irradiation utilizing **Osphen**. Here, it was also possible to increase the rate of the reaction by the addition of **PTC**, resulting in an improved yield of 86% under otherwise identical conditions (see Chapter S9.6).

## Conclusion

As has emerged from this study, short‐lived osmium complexes can be used for the efficient red light–driven generation of singlet oxygen when combined with a perylene salt in a Coulombic dyad system. The initial energy transfer from the excited osmium complex to the organic triplet state is essentially as fast as in traditional molecular dyad systems. Although the actual energy transfer kinetics are comparable with ion‐pairs containing dianionic and tetra‐anionic perylenes as organic counterparts, the association constants in the ground states determine the overall efficiencies for the Coulombic dyad photocatalysts under study. The pronounced preassociation of the **Osphen**–**PTC** dyad in solution via ion‐pairing facilitates the efficient formation of the long‐lived perylene triplet state with an excited state lifetime being more than three orders of magnitude longer than that of the osmium complex. This results in a significant kinetic advantage for singlet oxygen generation in aqueous solutions. Additionally, the higher singlet oxygen quantum yield for the quenching of the organic triplet state by oxygen further enhances the efficiency of the overall singlet oxygen production. The versatility of this system was demonstrated by the efficient photooxygenation of 5‐HMF, furfural, and dimethyl sulfide, as well as the effective degradation of the pollutant glyphosate in water. Moreover, the successful and selective generation of modafinil from a nonwater‐soluble precursor and the benzylamine oxidation in a less polar solvent mixture indicates a broad applicability and transferability of the straightforward Coulombic dyad concept to solvents beyond pure water.

## Supporting Information

The supporting information contains experimental details, additional steady‐state and time‐resolved spectroscopic results, quantum‐mechanical calculations, and details about the irradiation experiments. The authors have cited additional references within the Supporting Information.^[^ ^115^, ^116^, ^117^, ^118^, ^119^, ^120^, ^121^, ^122^, ^123^, ^124^, ^125^, ^126^, ^127^, ^128^, ^129^, ^130^, ^131^, ^132^, ^133^ ^]^

## Conflict of Interests

The authors declare no conflict of interest.

## Supporting information



Supporting Information

## Data Availability

The data that support the findings of this study are available in the main article and/or the Supporting Information. The data sets shown in the main paper and DFT output files are accessible via the JGU library (https://doi.org/10.25358/openscience‐12602) and the homepage of the senior corresponding author.
