# Supplementary material for: Efficient Red Light–Driven Singlet Oxygen Photocatalysis with an Osmium‐Based Coulombic Dyad
Source: Angew Chem Int Ed Engl. 2025 Jul 15;64(35):e202502840. doi: 10.1002/anie.202502840 (PMC12377441; doi:10.1002/anie.202502840)
Supplement: Supplementary file 1 — Supporting Information [file ANIE-64-e202502840-s001.pdf]

Supporting Information for

Efficient Red Light-Driven Singlet Oxygen Photocatalysis with an Osmium-Based Coulombic Dyad

Matthias Schmitz,*[a] Robert Naumann,[a] Katja Heinze[a] and Christoph Kerzig*[a]

[a] M. Schmitz, Dr. R. Naumann, Prof. Dr. K. Heinze, Prof. Dr. C. Kerzig
Department of Chemistry
Johannes Gutenberg University Mainz
Duesbergweg 10–14, 55128 Mainz, Germany
E-mail: smatthia@uni-mainz.de, ckerzig@uni-mainz.de

Table of contents

S1 Experimental section 3
S1.1 General experimental details 3
S1.2 Steady-state measurements 3
S1.3 Nanosecond laser flash photolysis 3
S1.4 Femtosecond transient absorption spectroscopy (fs-TAS) 4
S1.5 TCSPC measurements 4
S1.6 LED irradiation experiments 4
S1.7 $^1O_2$ assay 6
S1.8 NMR spectroscopy and HRMS spectrometry 6
S1.9 Quantum-chemical computations 7
S1.10 Cyclic voltammetry 8
S1.11 Summary of photophysical data 9
S2 Synthetic procedures 10
S3 Steady-state absorption and emission spectra 13
S4 NMR titration experiment 15
S5 Femtosecond transient absorption spectroscopy (fs-TAS) 17
S6 Quenching efficiencies of differently charged perylene derivatives or in strongly alkaline solution 21
S7 $^1O_2$ assay 23
S8 Cyclic voltammetry 26
S9 Photooxygenations 27
S9.1 Photooxygenation of **5-HMF** (**1**) 27
S9.2 Photooxygenation of **5** 36
S9.3 Photooxygenation of **8** 37
S9.4 Degradation of **10** 39
S9.5 Photooxygenation of **11** 41
S9.5.1 Spectroscopic investigations in MeOH/H_2O 43
S9.6 Photooxygenation of **13** 44
S10 Additional lifetime measurements 45
S11 NMR spectra 47
S12 Literature 52

S1 Experimental section
S1.1 General experimental details

Unless noted otherwise, all chemicals for optical spectroscopy and irradiation experiments were obtained commercially in high purity and used as received (acetone, ≥99.8%, Fisher Chemical; acetonitrile, 99.9%, VWR Chemicals; Amberlite IRA-410, Sigma-Aldrich; ammonium hexafluorophosphate, 98%, abcr; benzylamine (**13**), >99.0%, TCI; 2,2'-bipyridine (bpy), 95%, Alfa Aesar; deuterium oxide (D_2O), 99.90%, Deutero; tris(2,2'-bipyridyl)dichlororuthenium(II) hexahydrate (**Rubpy**), 99.95%, Sigma-Aldrich; *N*,*N*-dimethyl-4-nitrosoaniline, 98%, fisher scientific; dimethyl fumarate, 99%, Alfa Aesar; dimethyl sulfide (**8**), >99%, TCI; dimethyl sulfone, 98%, Sigma-Aldrich; [(diphenylmethyl)thio]acetamide (**11**), 98%, BLDpharm; ethanol abs., 99.8%, VWR Chemicals; ethylene glycol, ≥99%, Carl Roth; fumaric acid, >98%, Acros; furfural (**5**), 95%, Sigma-Aldrich; glyphosate (**10**), ≥94%, thermo scientific; 5-(hydroxymethyl)furfural (**1**), 98%, abcr; imidazole, >98%, TCI; maleic acid, >99%, TCI; methanol, ≥99.8%, VWR Chemicals; methanol-d_4, 99.80%, Deutero; methylene blue (**MB**), ≥97%, Sigma-Aldrich; modafinil (**12**), ≥98%, Sigma-Aldrich; perylene (**Per**), >98%, Alfa Aesar; 3,9-perylenedicarboxylic acid, ≥95%, Biosynth; 3,4,9,10-perylenetetracarboxylic 3,4:9,10-dianhydride, 97%, Sigma-Aldrich; 1,10-phenanthroline (phen) monohydrate, >98.0%, TCI; phosphoric acid, ≥85%, Sigma-Aldrich; potassium hexachloroosmate(IV), >99%, Sigma-Aldrich; potassium hydroxide, 85–100%, Honeywell; sodium acetate (anhydrous), >99%, Sigma-Aldrich; sodium chloride, >99.5%, fisher chemical; sodium hydroxide, ≥98%, Sigma-Aldrich; sodium phosphate dibasic (Na_2HPO_4), 97%, Sigma-Aldrich; sodium trifluoroacetate (**TFA**), >98%, TCI; tetrabutylammonium chloride, >99%, Sigma-Aldrich). Aqueous solutions were prepared using ultrapure Millipore MilliQ water with a specific resistance of 18.2 MΩ cm (20 °C). Prior to all spectroscopic measurements and irradiation experiments under oxygen-free conditions, argon from Nippon Gases (5.0) was bubbled through the sample solutions (approx. 3 ml) for 10 minutes to remove dissolved oxygen.

S1.2 Steady-state measurements
Absorption spectra were recorded using a *Perkin Elmer* LAMBDA 365 instrument. Steady-state emission measurements were performed with a *Perkin Elmer* FL-6500 spectrometer. All steady-state absorption and emission measurements were carried out at room temperature (20 ± 2 °C). Very low concentrations of the emitting substances were used to avoid filter effects.

S1.3 Nanosecond laser flash photolysis
For the recording of transient absorption spectra or time-resolved absorption or emission with ns laser pulses, a LP980KS setup from *Edinburgh Instruments* was used with either a Nd:YAG laser from *Litron* (Nano LG 300-10) or a Nd:YAG laser from *Quantel* (Q-smart 450) with frequency-doubled output (532 nm). For both lasers, the laser pulse duration was about 5 ns and the pulse frequency was 10 Hz. The excitation energy was adjusted with a mechanical attenuator (*Litron*) or by modifying the Q-switch delay (*Quantel*). Pulse energy measurements were conducted using a pyroelectric detector equipped with a broadband absorber sourced from *gentec-eo* (QE25LPS-MB-QED-D0). Unless otherwise specified, the intensity when using 532 nm as the excitation wavelength was ~20 mJ per laser pulse. To ensure a uniform excitation throughout the detection volume, beam expanders (*Thorlabs*) were employed. The beam diameter in front of the cuvette window was approximately 1.2 cm (*Litron*) and 0.8 cm (*Quantel*). This allowed accurate determination of the concentrations of the excited states in

the cuvette and provided reliable kinetic analyses. Transient absorption spectra were detected using an iCCD camera from *Andor*. Unless otherwise stated, transient absorption spectra were integrated over 100 ns. Single-wavelength kinetics were recorded using a *Hamamatsu* photomultiplier tube (R928). Spectroscopic experiments were performed using a temperature-controlled cell holder. Unless otherwise stated, LFP measurements were performed at 20 °C.

S1.4 Femtosecond transient absorption spectroscopy (fs-TAS)

fs-Transient absorption experiments were conducted using a Helios pump-probe setup from *Ultrafast Systems* paired with a regeneratively amplified 1030 nm laser (Pharos, *Light Conversion,* 1030 nm, < 175 fs, pulse energy: 2 mJ). The effective laser repetition rate of 1 kHz was set via an internal pulse picker. A small portion of the 1030 nm fundamental was directed to the optical delay line and subsequently used to generate broadband probe light by focusing the beam onto a sapphire for measurements in the Vis/NIR (450 nm – 900 nm). For measurements in the UV/Vis mode (330 nm – 500 nm) the second harmonic was focused onto a second sapphire instead of the fundamental. The pump pulse was generated with an optical parametric amplifier (Apollo Y, *Ultrafast Systems*). The sample solutions were measured in a 1 mm quartz cuvette. To generate spectra that cover the whole spectral region from 350 nm to 900 nm, the UV/Vis and Vis/NIR part of the transient absorption spectra were recorded separately under identical conditions and were combined using the overlap of both datasets in the visible region (475 nm – 525 nm). Preprocessing of the data, including chirp and baseline correction, was performed using the Surface Xplorer 4.3.0 software from *Ultrafast Systems*.

A global fit analysis for determining the time constants of intramolecular photoinduced processes was carried out using the well-established python package KiMoPack.[1] The relative error for the time constant determination by fs-TAS is estimated to be ~5%.

S1.5 TCSPC measurements

A *mini-*τ spectrometer from *Edinburgh Instruments* was used to determine the phosphorescence lifetime of **Osphen**. The pulsed lasers EPL-375 (λ_{exc} = 371 nm, pulse width: 58.3 ps) or EPL-450 (λ_{exc} = 446 nm, pulse width: 74.5 ps) from *Edinburgh Instruments* were used as excitation source. The desired detection range was set using the built-in bandpass filter. The internal response function (IRF) was determined by measuring stray light with a diluted ludox solution in the absence of any filters.

S1.6 LED irradiation experiments

Application-related irradiation experiments were conducted with a 660 nm LED from *Thorlabs* (Visible Mounted LED M660L4, 1050 mW output) with 100% output power.

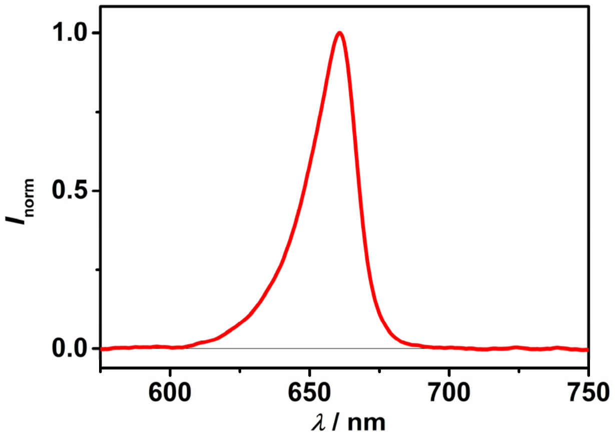

Figure S1 Emission spectrum of the used LED. The spectrum was measured by irradiation a water-containing cuvette in the FL-6500 instrument, recording the Rayleigh scattered light. The light source was installed in a 90° angle with respect to the detector.

All photooxygenations were carried out in a 22 ml glass vial with 5 ml of the reaction mixture. A water bath was used to ensure that the temperature of the irradiated solution was close to room temperature.

The photooxygenations were conducted with the *Thorlabs* 660 nm LED in combination with a collimator (*Thorlabs*, SM1U25-A), a T-Cube LED driver (*Thorlabs*, 1200 mA, LEDD1B) and a converging lens (*Thorlabs*, *f* = 60 mm, LB4592). The light from the LED was focused with the converging lens on the solution in the reaction vessel. The reaction vessel was sealed with a septum. A cannula whose needle tip ended in the headspace of the reaction vessel was connected to vacuum pump, which created a low negative pressure (25 ± 10 mbar below ambient pressure). Another cannula whose needle tip extended to the bottom of the reaction vessel and the end of which was connected to the ambient air led to a constant air flow through the reaction solution (see Figure S2). The LED was operated at full power (*I* = 1.2 A, int = 100%) during the entire irradiation period.

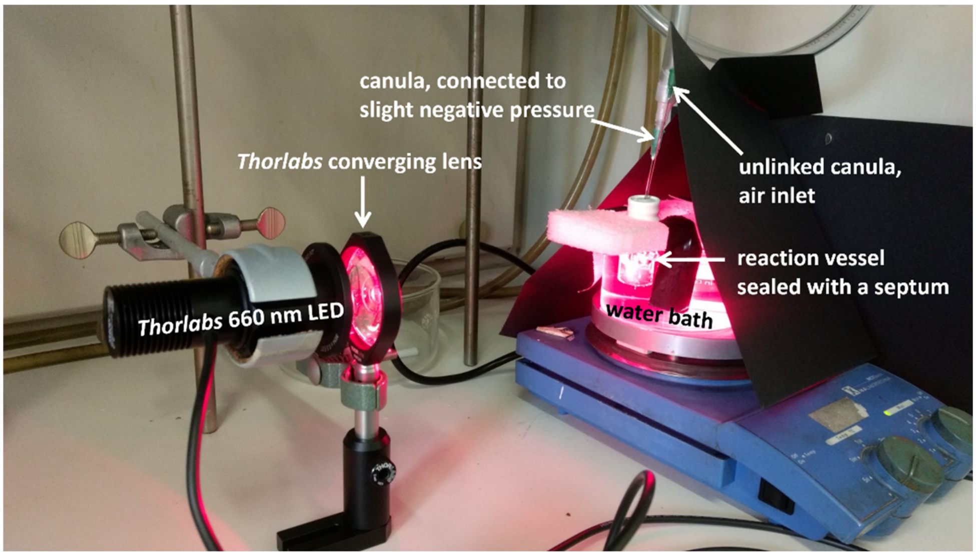

Figure S2 Reaction setup for the photooxygenation with the 660 nm *Thorlabs* LED.

The photooxygenations of **8**, **11** and **13** were carried out in a plastic stopper-sealed reaction vessel. In contrast to the setup presented above, no constant air flow was generated to prevent evaporation of the reactant or methanol during the reaction. The volume in the headspace of the reaction vessel contains approximately 1 equivalent of oxygen, which is sufficient to achieve a complete conversion.[2] The solution was stirred vigorously with a magnetic stir bar to ensure efficient oxygen uptake into the solution.

The reactions were carried out in non-deuterated solvents, with the exception of the synthesis of modafinil (**12**) and **14**. For reaction control for the reactions in water, a ^{1}H NMR spectrum was recorded from a mixture of 0.25 mL reaction solution and 0.35 mL D_2O. A very intense signal is obtained for water, centered at 4.79 ppm and extending approximately from 5.2 - 4.2 ppm, which has been cut out in the shown ^{1}H NMR spectra.

To quantify the reactant and products, the corresponding signals were compared to the signal from the internal standard. For photocatalysis yields below or above 10%, relative errors of 10% or 5% are expected, respectively.

S1.7 $^1O_2$ assay

For the $^1O_2$ assay, cuvettes with 2 mm pathlength were used, which were sealed with a *Teflon* stopper. The series of measurements summarized in one Figure (see S22–S24) were carried out with the same stock solution and an identical setup (see Figure S3) within a few hours. This ensures a reliable comparison of the relative $^1O_2$ formation rates. The used *Thorlabs* 660 nm LED was operated at an intensity of 100% for I = 0.35 A.

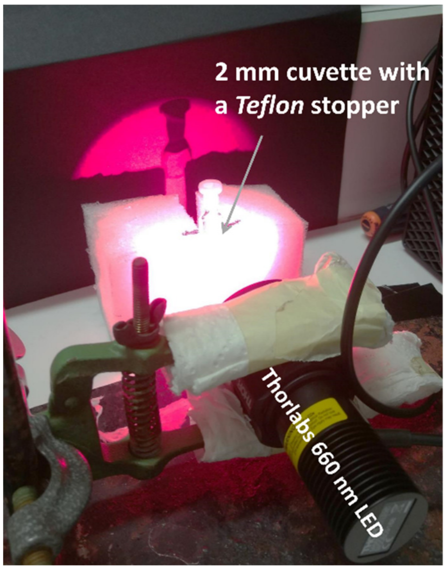

Figure S3 Irradiation setup for the $^1O_2$ assay.

S1.8 NMR spectroscopy and HRMS spectrometry

NMR spectroscopic studies were performed on an Avance III HD 300 MHz or Avance II 400 MHz multi nuclear magnetic resonance spectrometer from *Bruker* at 20 °C. The used solvents were water-d_2 or methanol-d_4 from *Deutero*. The ^{1}H NMR spectra were calibrated using the residual non-deuterated solvent.[3] ^{31}P NMR spectra were calibrated with 85% phosphoric acid as an external standard (δ= 0 ppm). In ^{19}F NMR spectroscopy, chemical shifts were referenced to trifluoroacetate (δ = -76.2 ppm).[4] Chemical shifts are given as δ values in ppm and all coupling constants are given in Hertz (Hz).

High resolution mass spectrometry (HRMS) was performed on a 6545 QTOF-HRAM-MS from *Agilent* using electrospray ionization (ESI).

S1.9 Quantum-chemical computations

Density Functional Theory (DFT) computations were performed using the program Orca 5.0.4.[5] The functional B3LYP and the basis set def2-SVP were used. After geometry optimizations, vibration frequencies were calculated. In each case, no imaginary vibrational frequencies were obtained, indicating convergence on minimum structures. The lowest triplet states were calculated starting from the energy-minimized singlet ground state geometries. Triplet state energies were determined by comparing the energies of the optimized structures of the singlet ground state and lowest triplet state for each compound. To determine electronic excitation energies and oscillator strengths of the lowest 30 transitions (see Figure S4), TD-DFT calculations were performed with previously geometry-optimized structures at the same level of theory. Additional single-point calculations were carried out to obtain the spin densities of the optimized triplet state based on Mulliken population analysis. The structures and spin densities (iso value: 0.005) were displayed using the software Avogadro1.2.0.[6] The color code for the atoms in the ball and stick representation is as follows: carbon (black), hydrogen (white), oxygen (red). In the depiction of spin densities, blue-colored surfaces denote positive spin densities, whereas red-colored surfaces denote negative spin densities.

Table S1: Predicted triplet-state energies based on DFT calculations.

**compound**	**E_T / eV**
**Per (charge: 0)**	1.49
**PDC (charge: –2)**	1.41
**PTC (charge: –4)**	1.30

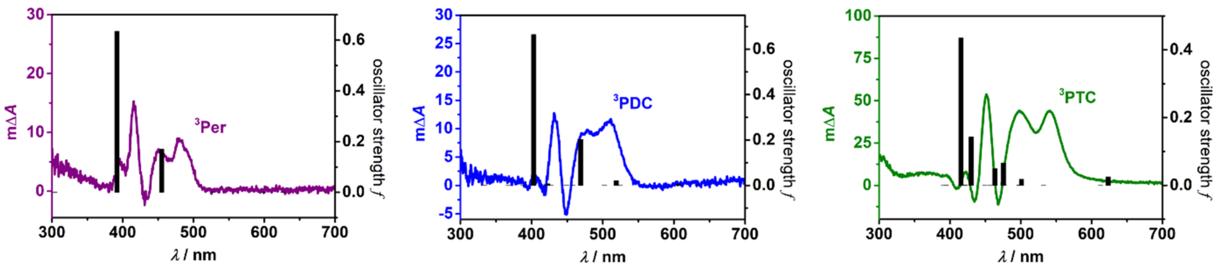

Figure S4 Experimental transient absorption spectra of the triplet states of the perylene species reported herein in acetonitrile (**Per**) or water (1mM NaOH, **PDC**, **PTC**) after laser excitation (λ_{exc} = 532 nm, *delay* 1 µs) in the presence of **Osphen** as the sensitizer (*c*(**Osphen**) = 16 µM, Cl salt in aqueous solution and PF_6 salt in acetonitrile, concentration of perylene derivatives: 30 µM) in combination with the calculated oscillator strengths for the $T_1 \rightarrow T_n$ transitions.

Table S2: Structures of the S_0 and T_1 states and spin densities of the T_1 states of the perylene derivatives.

compound	structure S_0 state	structure T_1 state	spin density T_1 state
**Per**	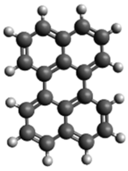	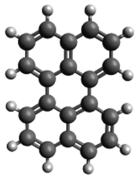	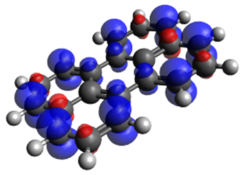
**PDC**	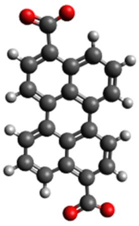	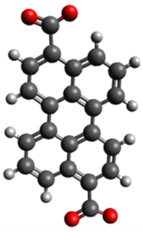	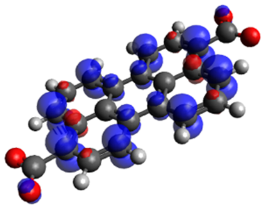
**PTC**	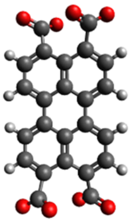	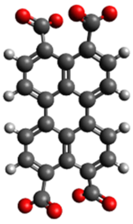	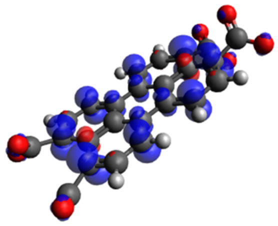

S1.10 Cyclic voltammetry

Cyclic voltammetry was performed with an EmStat4s potentiostat from *PalmSens*. A glassy carbon working electrode, a Pt wire counter electrode and an Ag/AgCl (3M NaCl, +0.209 V vs NHE) reference electrode were used. All measurements were carried out in an aqueous 0.1 M Na_2HPO_4 solution. The measured potentials are given against SCE (+0.250 V vs NHE)[7] and were converted using E(SCE) = E(Ag/AgCl (3 M NaCl)) – 0.041 V.

S1.11 Summary of photophysical data

Table S3: Summary of the key photophysical data for the Coulombic dyad systems consisting of **Osphen** and **PDC** or **Osphen** and **PTC** in 1 mM NaOH.

	**Osphen (reference)**	**PDC**[a]	**PTC**[a]
τ_T	77.2 ns	147 μs	219 μs
E_T	1.80 eV[b]	1.41 eV[c]	1.30 eV[c]
k_q[d]	-	$1.17{\cdot}10^{10}$ $M^{-1}s^{-1}$	$4.0{\cdot}10^{10}$ $M^{-1}s^{-1}$
K_s[e]	-	$3.6{\cdot}10^{3}$ M^{-1}	$1.33{\cdot}10^{4}$ M^{-1}
η_{static} [f]	-	0.24	0.54
$\eta_{dynamic}$[f]	-	0.06	0.09
τ_{EnT}[g]	-	91 ps	102 ps
Triplet quenching efficiency by dissolved O_2	0.073	~1	~1
Rel. $^1O_2$ formation rate[h]	1	4.86	15.5

[a] In combination with **Osphen** as sensitizer. [b] From ref. [8]. [c] Determined by DFT at B3LYP/def2-SVP level of theory. [d] Dynamic quenching of ^{3}**Osphen** by perylene derivative. [e] Static quenching between **Osphen** and perylene derivative. [f] Quenching of ^{3}**Osphen** for *c*(**Osphen**) = 16 μM and a concentration of the perylene salt of 90 μM. Dynamic quenching has been corrected for initial static quenching. [g] Time constant of the energy transfer from ^{3}**Osphen** to yield the triplet state of the respective perylene derivative. [h] For *c*(**Osphen**) = 15 μM and a concentration of the perylene salt of 45 μM.

S2 Synthetic procedures

Synthesis of tris(1,10-phenanthroline)osmium(II) dichloride (Osphen)

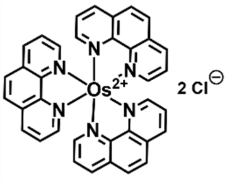

Osphen was prepared by a modified procedure of Constable *et al.*[9] 1,10-Phenanthroline monohydrate (0.20 g, 1.00 mmol, 3.0 eq), potassium hexachloroosmate(IV) (0.16 g, 0.33 mmol, 1.0 eq) and ethylene glycol (100 ml) were added to a three-necked 250 ml round-bottom flask equipped with a Dimroth condenser. Argon was bubbled though the solution for 30 minutes to remove any dissolved oxygen. The solution was then heated at reflux for 2 h. After cooling to room temperature, the reaction mixture was poured into 100 ml of an aqueous solution containing ammonium hexafluorophosphate (1.00 g, 6.10 mmol, 18.0 eq) and stirred for 15 minutes. A black precipitate formed, which was filtered out, washed several times with water and dried under vacuum to yield $[Os(phen)_3](PF_6)_2$ as a black solid (0.29 g, 0.28 mmol, 85%).

HRMS, ESI (*m*/*z*): Calculated for $[M\text{-}2PF_6]^{2+}$: 366.0839, found 366.0835 (100% rel. intensity); Calculated for $[M\text{-}PF_6]^{+}$: 877.1319, found 877.1321 (7.3% rel. intensity). Calculated for $[M\text{-}2PF_6\text{+}Cl]^{+}$: 767.1366, found - (not found).

Anion exchange was carried out with the ion exchange resin Amberlite IRA-410. 50 g of this resin was first washed several times with 40 ml ethanol until the wash solution remained clear. The resin then swelled in ethanol in a glass column for 24 hours. To load the resin with chloride, it was rinsed four times with 40 ml of an aqueous 10 mM NaCl solution, with the aqueous solution remaining in the column for one hour in each run. The column was then washed three times with 40 ml methanol. $[Os(phen)_3](PF_6)_2$ (65 mg, 0.064 mmol) was dissolved in 15 ml methanol and filled into the column. After 2 h, the solution was drained and rinsed five times with 30 ml methanol each, so that the solution of the last washing step remained colorless. The combined organic phases were evaporated to dryness under reduced pressure. This yielded $[Os(phen)_3]Cl_2$ (**Osphen**) as a black solid (51 mg, 0.064 mmol, 100%).

HRMS, ESI (*m*/*z*): Calculated for $[M\text{-}2Cl]^{2+}$: 366.0839, found 366.0839 (100% rel. intensity); Calculated for $[M\text{-}Cl]^{+}$: 767.1366, found 767.1356 (0.56% rel. intensity). Calculated for $[M\text{-}2Cl\text{+}PF_6]^{+}$: 877.1319, found 877.1322 (0.27% rel. intensity).
^{1}H NMR (300 MHz, D_2O): δ / ppm = 8.35 (dd, *J* = 8.3, 1.2 Hz, 6H), 8.21 (s, 6H), 8.00 (dd, *J* = 5.4, 1.2 Hz, 6H), 7.51 (dd, *J* = 8.3, 5.4 Hz, 6H).
UV-Vis spectrum in H_2O (rel. absorbance): λ / nm = 560 (0.049), 478 (0.17), 432 (0.18), 316 (0.058), 264 (1.0). $\varepsilon_{431\ nm} = 1.73\cdot10^4\ M^{-1}cm^{-1}$.

The analytical data sets are in agreement with literature values.[10]

In the HRMS analysis, both the **Osphen** di-cations and the ions with one counterion each (PF_6^- or Cl^-) were measured, whereby the PF_6^- anion had a higher tendency to be present as an ion-pair with **Osphen** during the measurement (higher relative intensity). In the chloride salt, traces of the **Osphen** di-cation paired with a single PF_6^- anion can be found. In a semi-quantitative ^{19}F NMR experiment using sodium trifluoroacetate (**TFA**) and ammonium hexafluorophosphate in well-defined concentrations as internal standards, it was concluded that the PF_6^- content in the chloride salt is <0.5 % (see Figure S67) and thus an essentially quantitative ion exchange was achieved.

Synthesis of tris(2,2‘-bipyridine)osmium(II) dichloride (Osbpy)

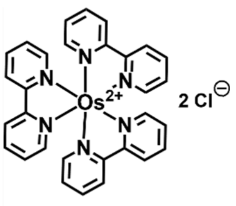

Osbpy

Osbpy was prepared by a modified procedure of Constable *et al.*[9] 2,2‘-Bipyridine (157 mg, 1.0 mmol, 3.1 eq), potassium hexachloroosmate(IV) (156 mg, 0.32 mmol, 1.0 eq) and ethylene glycol (100 ml) were added to a three-necked 250 ml round-bottom flask equipped with a Dimroth condenser. Argon was bubbled though the solution for 45 minutes to remove any dissolved oxygen. The solution was then heated at reflux for 2 h. The black reaction mixture was cooled to room temperature and added to 100 ml of water in which ammonium hexafluorophosphate (1.2 g, 7.4 mmol, 23 eq) was previously dissolved. The solution was filtered and the black residue was washed 3 times with 30 ml water each and dried under vacuum. This yielded $[Os(bpy)_3](PF_6)_2$ as a black solid (213 mg, 0.23 mmol, 72%).

HRMS, ESI (*m*/*z*): Calculated for $[M\text{-}2PF_6]^{2+}$: 330.0839, found 330.0836 (100% rel. intensity); Calculated for $[M\text{-}PF_6]^{+}$: 805.1319, found 805.1321 (17% rel. intensity). Calculated for $[M\text{-}2PF_6\text{+}Cl]^{+}$: 695.1366, found - (not found).

71.0 mg of this salt (0.075 mmol) were dissolved in 5 ml acetone and mixed with 2 ml of a saturated solution of tetrabutylammonium chloride in acetone. After 2 h storage at 4 °C, the solution was filtered and the black solid was washed five times with 5 ml acetone each. This yielded $[Os(bpy)_3]Cl_2$ (**Osbpy**) as a black solid (13.7 mg, 0.019 mmol, 25%).

HRMS, ESI (*m*/*z*): Calculated for $[M\text{-}2Cl]^{2+}$: 330.0839, found 330.0836 (100% rel. intensity); Calculated for $[M\text{-}Cl]^{+}$: 695.1366, found 695.1355 (0.14% rel. intensity). Calculated for $[M\text{-}2Cl\text{+}PF_6]^{+}$: 805.1319, found 805.1321 (0.48% rel. intensity).
^{1}H NMR (300 MHz, D_2O): δ / ppm = 8.49 (d, *J* = 8.2 Hz, 6H), 7,88–7,77 (m, 6H), 7.72 (d, *J* = 5.7 Hz, 6H), 7.25 (ddd, *J* = 7.5, 5.8, 1.3 Hz, 6H).
UV-Vis spectrum in H_2O (rel. absorbance): λ / nm = 590 (0.0340), 480 (0.15), 435, (0.14), 385 (0.11), 367 (0.12), 330 (0.12), 290 (1.0), 245 (0.32). $\varepsilon_{480\ nm} = 1.14 \cdot 10^{4}\ M^{-1}cm^{-1}$.

The analytical data sets are in agreement with literature values.[10]

In the HRMS analysis, both the **Osbpy** dications and the ions with one counterion each (PF_6 or Cl) were measured, whereby the PF_6 anion had a higher tendency to be present as an ion-pair with **Osbpy** during the measurement (higher relative intensity). In the chloride salt, traces of the **Osbpy** di-cation paired with a single PF_6^- anion can be found. In a semi-quantitative ^{19}F NMR experiment using sodium trifluoroacetate (**TFA**) and ammonium hexafluorophosphate in well-defined concentrations as internal standards, it was concluded that the PF_6^- content in the chloride salt is <0.5 % (see Figure S68) and thus an essentially quantitative ion exchange was achieved.

Synthesis of 3,4,9,10-perylenetetracarboxylic acid tetrapotassium salt (PTC)

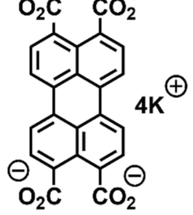

PTC

PTC was prepared by a modified procedure of Wang *et al.*[11] In a 250 ml round-bottom flask equipped with a Dimroth condenser, 3,4,9,10-perylenetetracarboxylic-3,4:9,10-dianhydride (1.98 g, 5.1 mmol, 1.0 eq) and potassium hydroxide (1.12 g, 20.4 mmol, 4.0 eq) were added to 75 ml of water. The reaction solution was heated for 18 h under reflux. After heating, the initially red suspension yielded a yellow-red, clear solution. The solvent was removed under reduced pressure and the orange solid was washed three times with 60 ml abs. ethanol each time. The solid was dried under reduced pressure. This yielded an orange solid of **PTC** (2.70 g, 4.7 mmol, 93%).

^{1}H NMR (300 MHz, D_2O): δ / ppm = 8.40 (d, J = 7.9 Hz, 4H), 7.79 (d, J = 7.8 Hz, 4H).
UV-Vis spectrum in H_2O (rel. absorbance): λ / nm = 439 (0.81), 466 (1.0).

The analytical data sets are in agreement with literature values.[11]

Synthesis of 3,9-perylenedicarboxylic acid dipotassium salt (PDC)

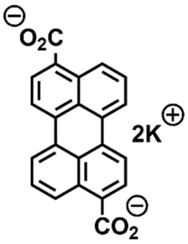

PDC

PDC was prepared by a modified procedure of Wang *et al*.[11] In a 50 ml round-bottom flask, 3,9-perylenedicarboxylic acid (500 mg, 1.5 mmol, 1.0 eq) was mixed with water (6.62 ml) and potassium hydroxide (170 mg, 3.0 mmol, 2.0 eq). The reaction mixture was heated at 100 °C and stirred for 30 min. After cooling to room temperature, the solvent was removed under reduced pressure. 599 mg of a brownish solid was obtained. The crude product was crushed in a mortar, washed twice with 10 ml abs. ethanol and dried under reduced pressure. This yielded a yellow solid of **PDC** (481 mg, 1.2 mmol, 80%).

^{1}H NMR (300 MHz, D_2O): δ / ppm = 8.00–7.83 (m, 6H), 7.49 (t, J = 7.7 Hz, 2H), 7.33 (dt, J = 13.4, 8.0 Hz, 2H).
UV-Vis spectrum in H_2O (rel. absorbance): λ / nm = 420 (0.81), 446 (1.0).

The analytical data sets are in agreement with literature values.[12]

S3 Steady-state absorption and emission spectra

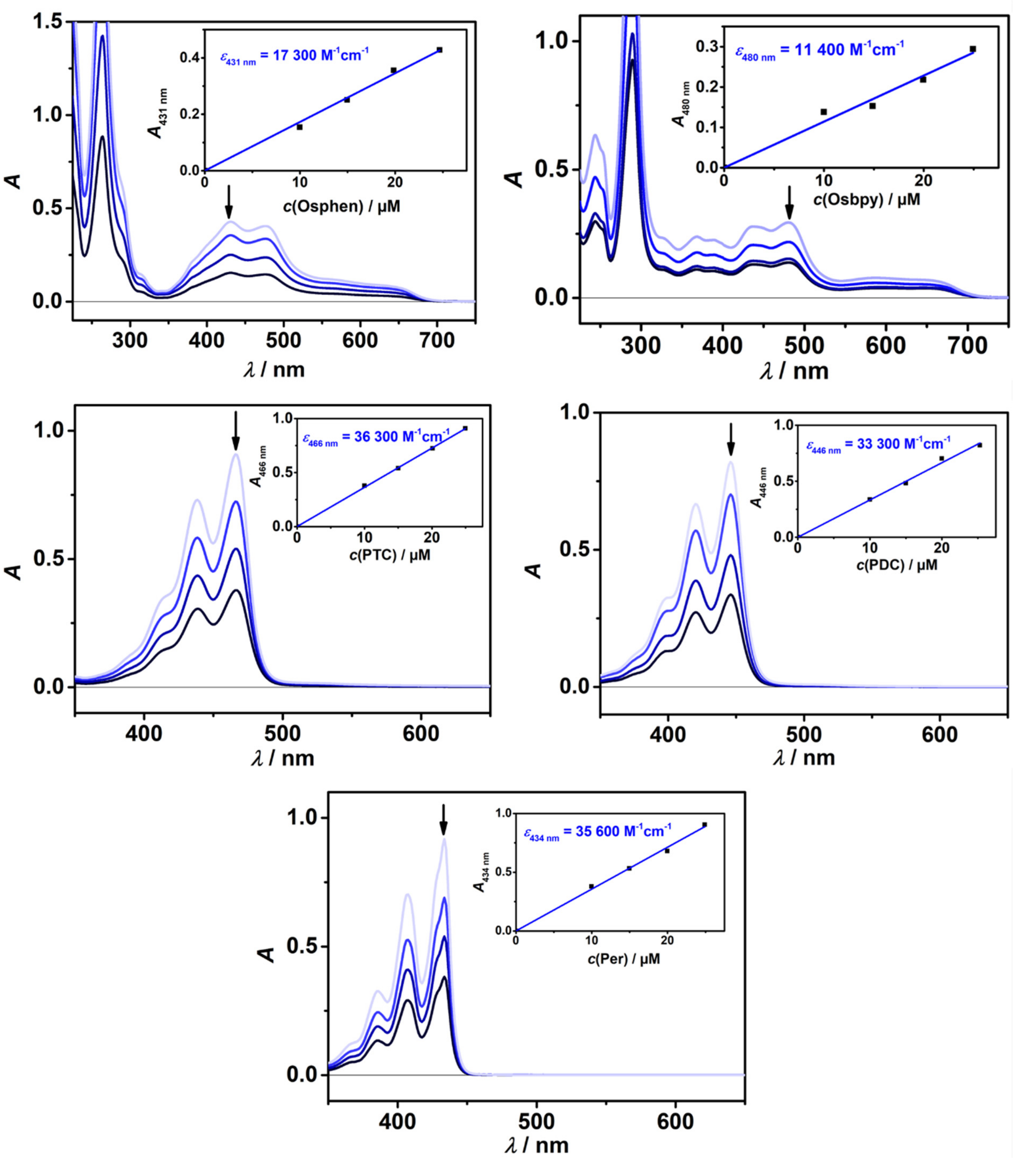

Figure S5 Determination of ground-state molar absorption coefficients of the used osmium complexes and perylene derivatives in water (**Osphen**, **Osbpy**, **PTC**, **PDC**) or acetonitrile (**Per**).

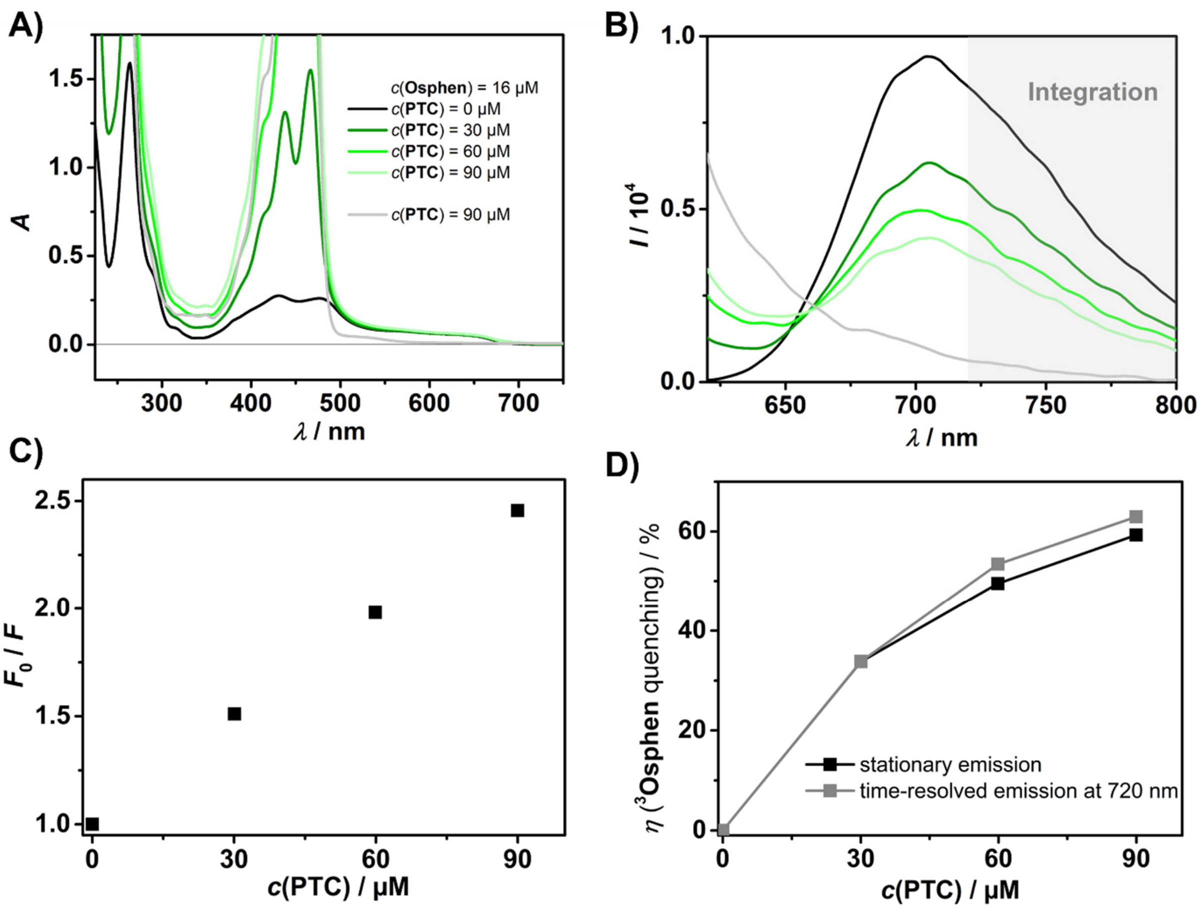

Figure S6 (A) UV-Vis spectra of aqueous solutions (1 mM NaOH) containing **Osphen** with **PTC** in different concentrations or **PTC** alone. (B) Stationary emission spectra of the same samples in Ar-saturated solution (λ_{exc} = 600 nm) with the indication of the integration window for the Stern–Volmer analysis (gray box). (C) Corresponding Stern–Volmer plot. (D) Comparison of the efficiency of ^{3}**Osphen** quenching derived from stationary or time-resolved emission spectroscopy (see Figure 2 in the main part).

PTC and its dimers/aggregates absorb very weakly at 532 nm,[13] resulting in artifacts due to their fluorescence in both the time-resolved and the steady-state emission measurements. The filter effect due to **PTC** absorption is considered to be negligible. In order to keep the influence of this emission as low as possible, in the case of time-resolved emission the exponential fits were applied after the fluorescence had decayed (see Figure 2 of main part) and in the case of stationary emission, the spectra were integrated in the range from 720–800 nm. Based on the outcome of the time-resolved quenching study, the Stern–Volmer plot in Figure S6 C should contain contributions of static and dynamic quenching. A quadratic curve is therefore expected,[14,15] but the result appears to be linear. This can be explained by the fact that only minor dynamic quenching takes place due to the very short lifetime of ^{3}**Osphen** and static quenching dominates due to the very high association constant. If one of the quenching processes dominates, the overall curve appears linear.[14] If both processes are roughly equally important, a textbook-like upward curvature is observed.[15] The quenching efficiencies from the stationary emission are comparable to those from the time-resolved emission (see Figure S6 D). The efficiencies from the stationary emission tend to be slightly lower, which is due to the minimal contamination caused by the emission of **PTC**.

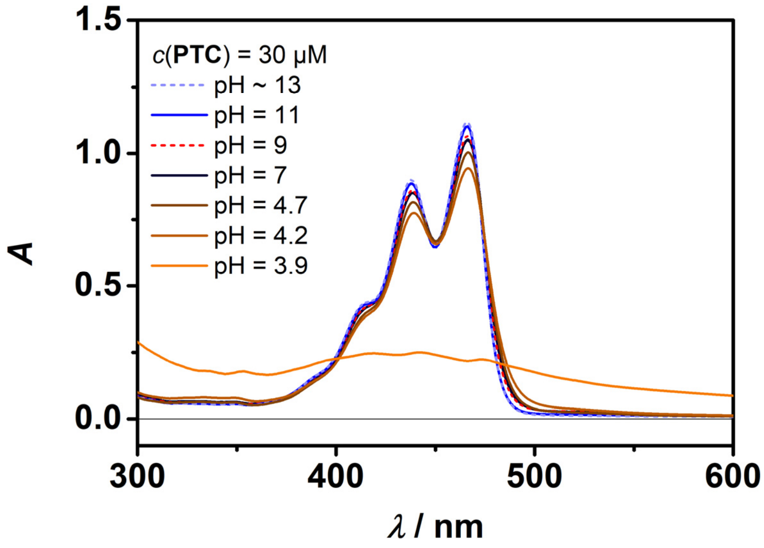

Figure S7 pH-dependent UV-Vis spectra of an aqueous solution of **PTC** (c(**PTC**) = 30 µM, pH = 3.9: $c(H_2SO_4)$ = 60 µM, pH = 4.2: $c(H_2SO_4)$ = 30 µM, pH = 4.7: $c(H_2SO_4)$ = 10 µM, pH = 7: no additive, pH = 9: c(NaOH) = 10 µM, pH = 11: c(NaOH) = 1 mM, pH ~ 13: c(NaOH) = 0.1 M). The recorded spectrum at a pH value of the solvent of 3.9 is mainly influenced by scattering effects caused by the precipitation of **PTC**.

The UV-Vis spectra of **PTC** look similar at a pH of 7 and 9 or 11 and 13 (see Figure S7). A change in the shape of the absorption band can be observed between pH 9 and 11. This is most likely due to the fact that **PTC** is not in a fully deprotonated state at/around neutral pH. Since the absorption spectrum does not change between a pH of 11 and the strongly alkaline pH of ~13, it is assumed that **PTC** is already in its fully deprotonated state at a concentration of c(NaOH) = 1 mM. Therefore, the time-resolved and steady-state optical spectroscopy and the applications are performed in an aqueous solution with 1 mM NaOH. As the pH of the solvent decreases below 7, the shape of the spectrum also changes, apparently due to further protonation of **PTC** to the point where **PTC** is most likely fully protonated and precipitates out of solution (pH = 3.9).

S4 NMR titration experiment

In an NMR titration experiment, **PTC** (guest compound) was added in varying amounts to solutions with a constant **Osphen** concentration (0.48 mM, host compound) in D_2O (with 1 mM NaOD) as solvent. In the corresponding ^{1}H NMR spectra (see Figure S8), it can be seen that the chemical shifts of **Osphen** undergo an upfield shift with increasing amounts of **PTC**. Additionally, the chemical shifts of the protons of **PTC** also undergo an upfield shift for increasing c(**Osphen**):c(**PTC**) ratios (or lower c(**PTC**)). This indicates ion-pairing interactions of the two chromophores in solution. In addition to these shifts, there is also a broadening of the **Osphen** signals, so that only the chemical shifts of **Osphen** whose positions can be reliably determined (for c(**PTC**) = 0–0.60 mM) are taken into account for the determination of the association constant based on this titration experiment (see Figure S8).

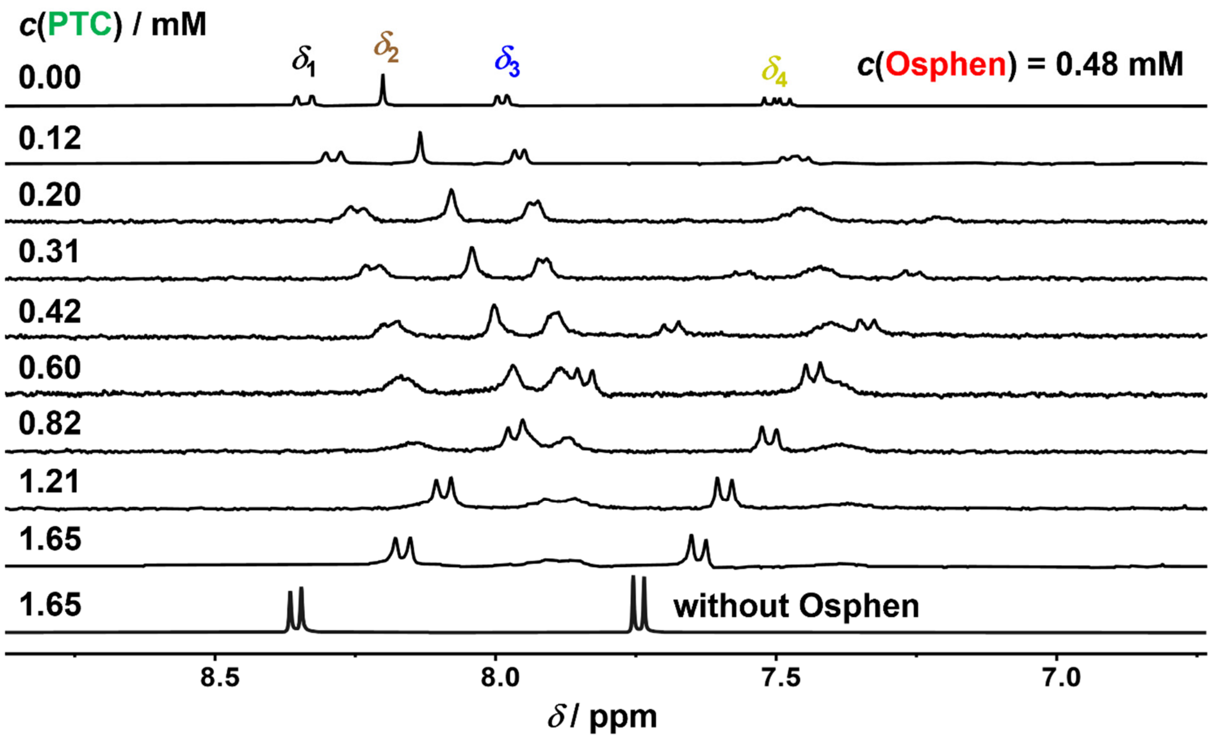

Figure S8 ^{1}H NMR spectra of **Osphen** (signals labeled with δ_1–δ_4) in the presence of **PTC** at different concentrations in D_2O (with 1 mM NaOD) at 20 °C.

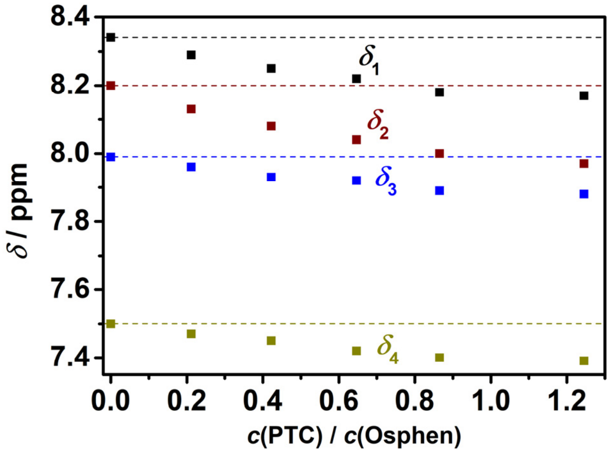

Figure S9 Chemical shifts of the ^{1}H NMR spectra of **Osphen** (signals labeled with δ_1–δ_4) at different guest:host ratios (*c*(**PTC**)/*c*(**Osphen**), with *c*(**PTC**) = 0–0.60 mM) in D_2O (1 mM NaOD) at 20 °C.

The association constant for ion-pairing based on the NMR titration experiment was determined by the program BindFitV0.5[16,17] from the Thordarson group. As recommended by the Thordarson group, the fit as well as the raw data and metadata can be accessed via the following link:

http://app.supramolecular.org/bindfit/view/82e78eec-4ee7-4718-aa2d-4955732f0d3f

A global fit based on a 1:1 aggregation model is in good agreement with the raw data. A fit based on a 2:1 (**Osphen**:**PTC**) model resulted in negative values for one association constant, which is not considered physically reasonable. A 1:2 (**Osphen**:**PTC**) aggregation is not

considered meaningful due to the very high charge of **PTC** with -4 compared to **Osphen** with +2. The association constant K_{11} for the ion-pairing between **Osphen** and **PTC** in D_2O is:

$$K_{11} = (1.9 \pm 0.4) \cdot 10^4\ \mathrm{M}^{-1}.$$

S5 Femtosecond transient absorption spectroscopy (fs-TAS)

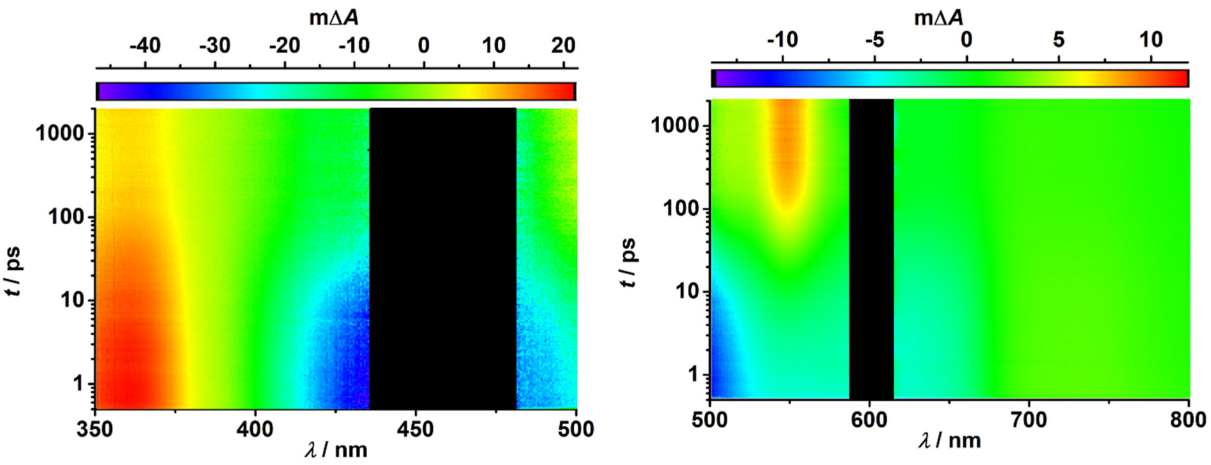

Figure S10 Contour plots of the fs-TAS (UV/Vis (left) and Vis/NIR channel (right)) of an aqueous solution (1 mM NaOH) containing *c*(**Osphen**) = 0.65 mM and *c*(**PTC**) = 0.46 mM after laser excitation (λ_{exc} = 600 nm, pulse length: <175 fs).

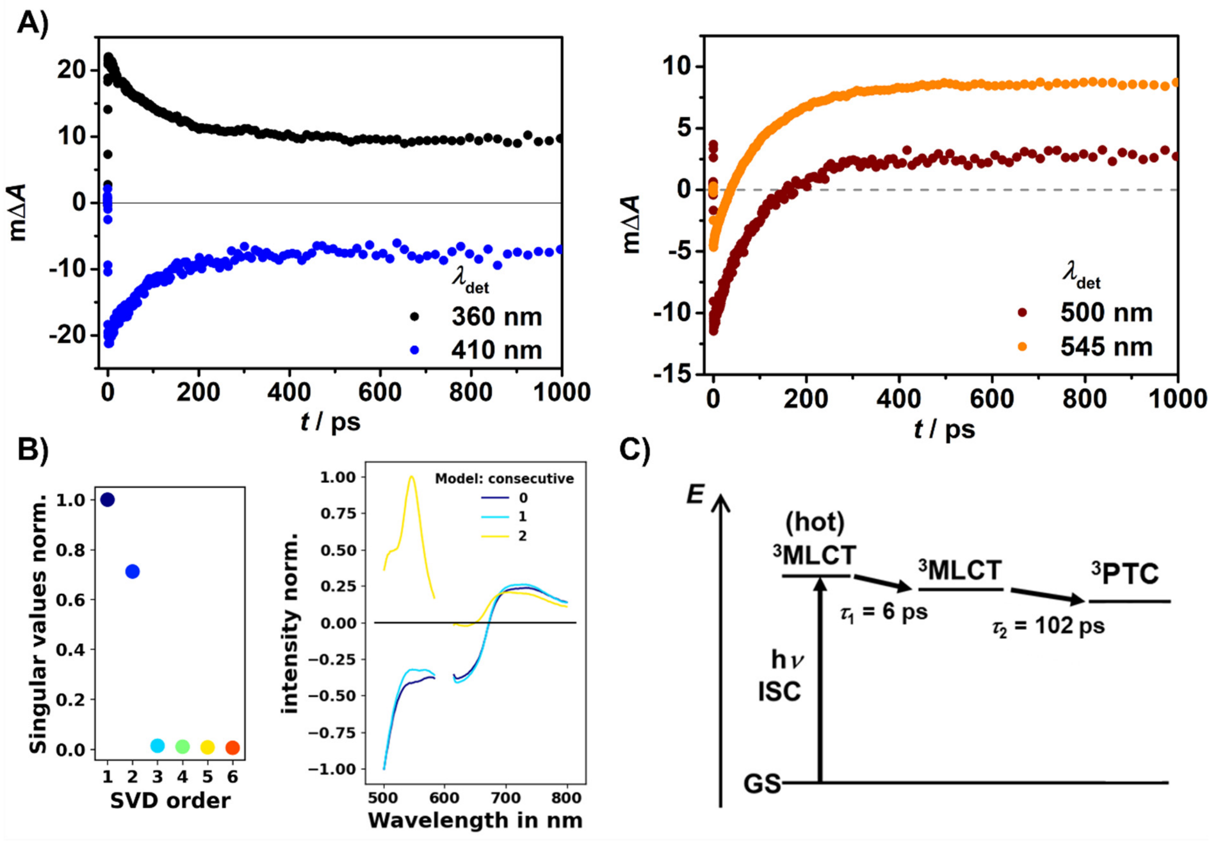

Figure S11 (A) Transient absorption traces at selected wavelengths with pronounced signal change from the measurement shown in Figure S10 and Figure 3 in the main part. (B) Singular value decomposition (SVD) analysis and decay associated spectra of a three-component consecutive fitting model (from 500–800 nm, wavelength range from 585–615 nm was omitted due to light scattering from laser excitation, time range from t = -0.5–0.5 ps was omitted). (C) Schematic representation of the photophysical processes upon photoexcitation of **Osphen** ion-paired to **PTC** with corresponding time constants from the global fit analysis. The shorter lifetime is associated with vibrational cooling.

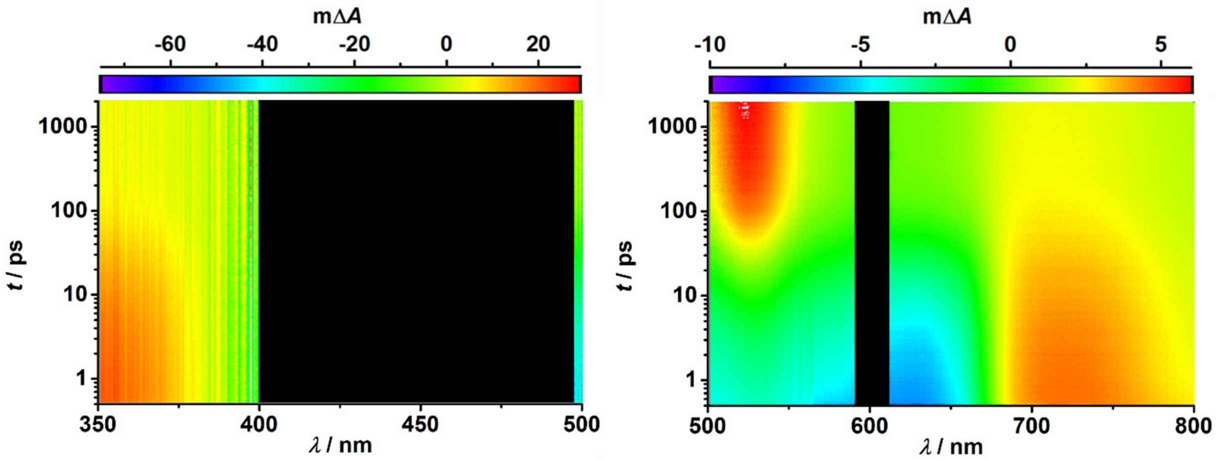

Figure S12 Contour plots of the fs-TAS (UV/Vis (left) and Vis/NIR channel (right)) of an aqueous solution (1 mM NaOH) containing *c*(**Osphen**) = 0.65 mM and *c*(**PDC**) = 0.90 mM after laser excitation (λ_{exc} = 600 nm, pulse length: <175 fs).

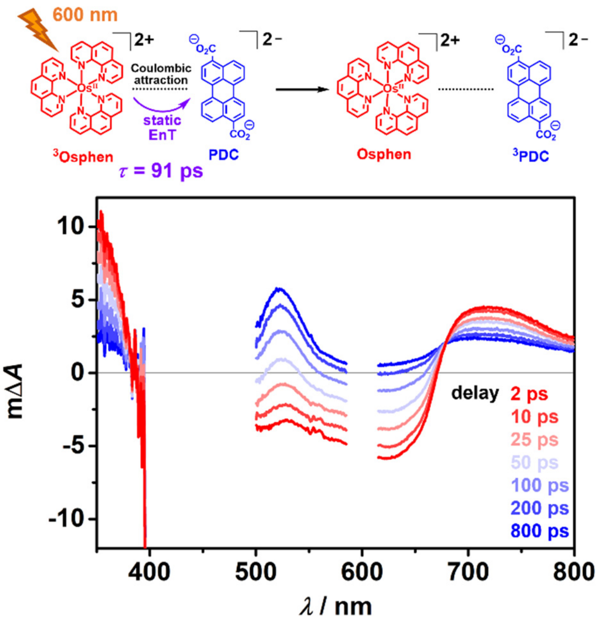

Figure S13 Scheme of the initial intra-ion-pair energy transfer step between **Osphen** and **PDC** (top). TA spectra (bottom) of a solution containing *c*(**Osphen**) = 0.65 mM and *c*(**PDC**) = 0.90 mM in an aqueous solution (1 mM NaOH) recorded at certain delay times after laser excitation (λ_{exc} = 600 nm, pulse length: <175 fs). Data points around 450 nm and 600 nm are omitted due to artefacts caused by strong filter effects by **PDC** or scattering of the excitation light, respectively.

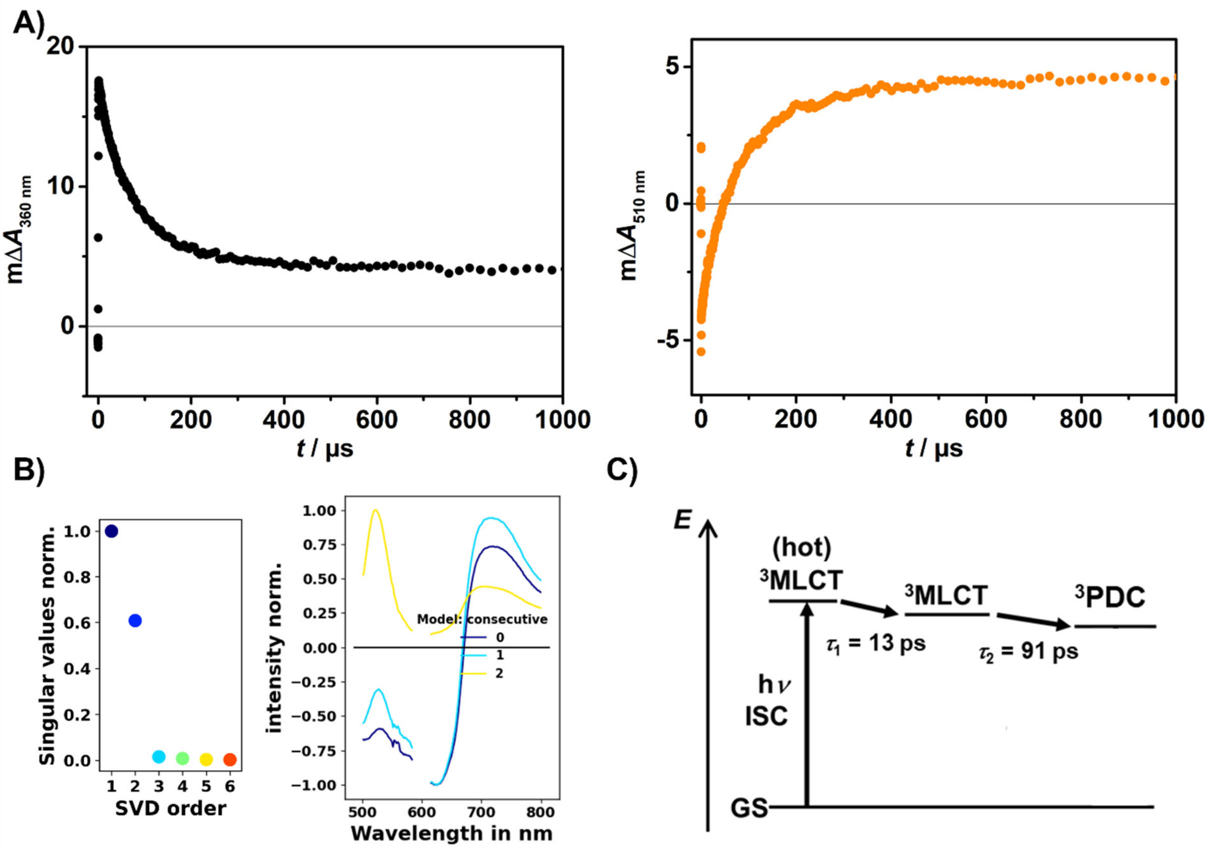

Figure S14 (A) Transient absorption traces at selected wavelengths with pronounced signal change from the measurement shown in Figure S12 and Figure S13. (B) Singular value decomposition (SVD) analysis and decay associated spectra of a three-component consecutive fitting model (from 500–800 nm, wavelength range from 585–615 nm was omitted due to light scattering from laser excitation, time range from t = -0.5–0.5 ps was omitted). (C) Schematic representation of the photophysical processes upon photoexcitation of **Osphen** ion-paired to **PDC** with corresponding time constants from the global fit analysis. The shorter lifetime is associated with vibrational cooling.

Under the selected concentrations, about 64% of the ground state Os complex molecules exist as **Osphen–PDC** ion-pair and dynamic quenching is predicted to occur with a time constant of 43 ns. These conditions allow us to observe and analyze the ultrafast static quenching process in isolation.

S6 Quenching efficiencies of differently charged perylene derivatives or in strongly alkaline solution

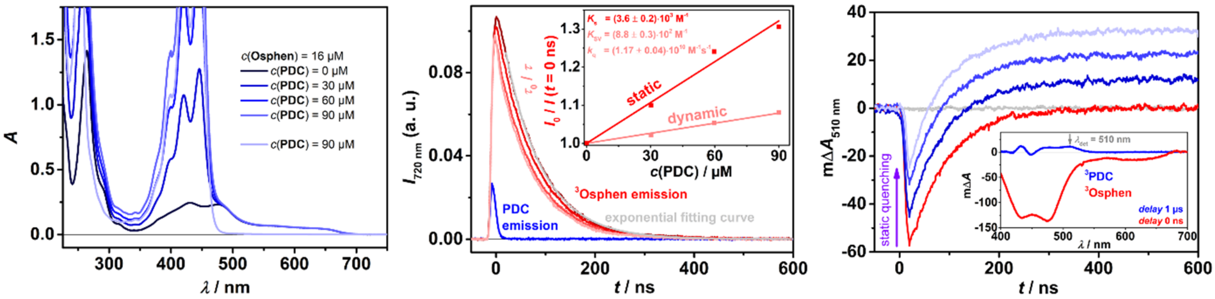

Figure S15 (left) UV-Vis spectra of aqueous solutions (1 mM NaOH) containing **Osphen** and **PDC** in different concentrations or only **PDC**. (middle) Corresponding time-resolved emission at λ_{det} = 720 nm after laser excitation (λ_{exc} = 532 nm) in Ar-saturated solution along with the Stern–Volmer plot (inset). (right) Corresponding time-resolved absorption at λ_{det} = 510 nm after laser excitation (λ_{exc} = 532 nm) in Ar-saturated solution. (right, inset) Transient absorption spectra of a solution containing 16 µM **Osphen** (red) and 30 µM **PDC** (blue) 1 µs after laser excitation (λ_{exc} = 532 nm) in Ar-saturated aqueous solution as well as a reference spectrum with **Osphen** alone.

The contribution of static and dynamic quenching was determined in the same way as for **Osphen** and **PTC** (see main part of the manuscript).

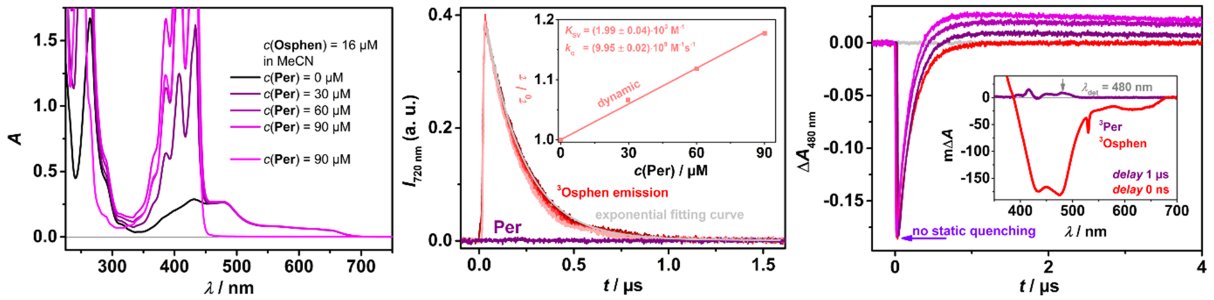

Figure S16 (left) UV-Vis spectra of solutions containing **Osphen** (PF_6 salt) and **Per** in different concentrations or **Per** in acetonitrile. (middle) Corresponding time-resolved emission at λ_{det} = 720 nm after laser excitation (λ_{exc} = 532 nm) in Ar-saturated solution along with the Stern–Volmer plot (inset). (right) Corresponding time-resolved absorption at λ_{det} = 510 nm after laser excitation (λ_{exc} = 532 nm) in Ar-saturated solution. (right, inset) Transient absorption spectra of a solution containing 16 µM **Osphen** (PF_6 salt, red) and 30 µM **Per** (blue) 1 µs after laser excitation (λ_{exc} = 532 nm) in Ar-saturated acetonitrile as well as a reference spectrum with **Osphen** alone. The TA spectrum of ^{3}**Per** is very similar to that reported in literature.[18]

For a better comparison of the quenching studies in water and acetonitrile, the quenching efficiency of dynamic quenching in acetonitrile was calculated using the obtained k_q in acetonitrile together with the unquenched lifetime of ^{3}**Osphen** in water (77 ns), which is 2.6 times shorter compared to that in acetonitrile (~200 ns). This represents an upper limit for the quenching efficiency since the rate of diffusion in acetonitrile is typically higher than in water. The result is shown in Figure S17.

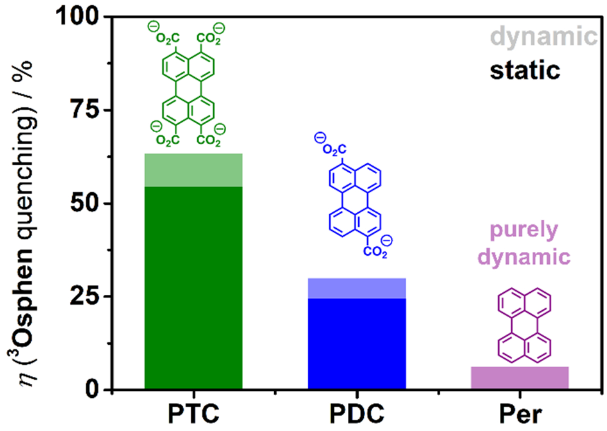

Figure S17 Comparison of static and dynamic quenching efficiencies of ^{3}**Osphen** with 90 µM of **PTC**, **PDC** or **Per** as quenchers in solution. See text for details.

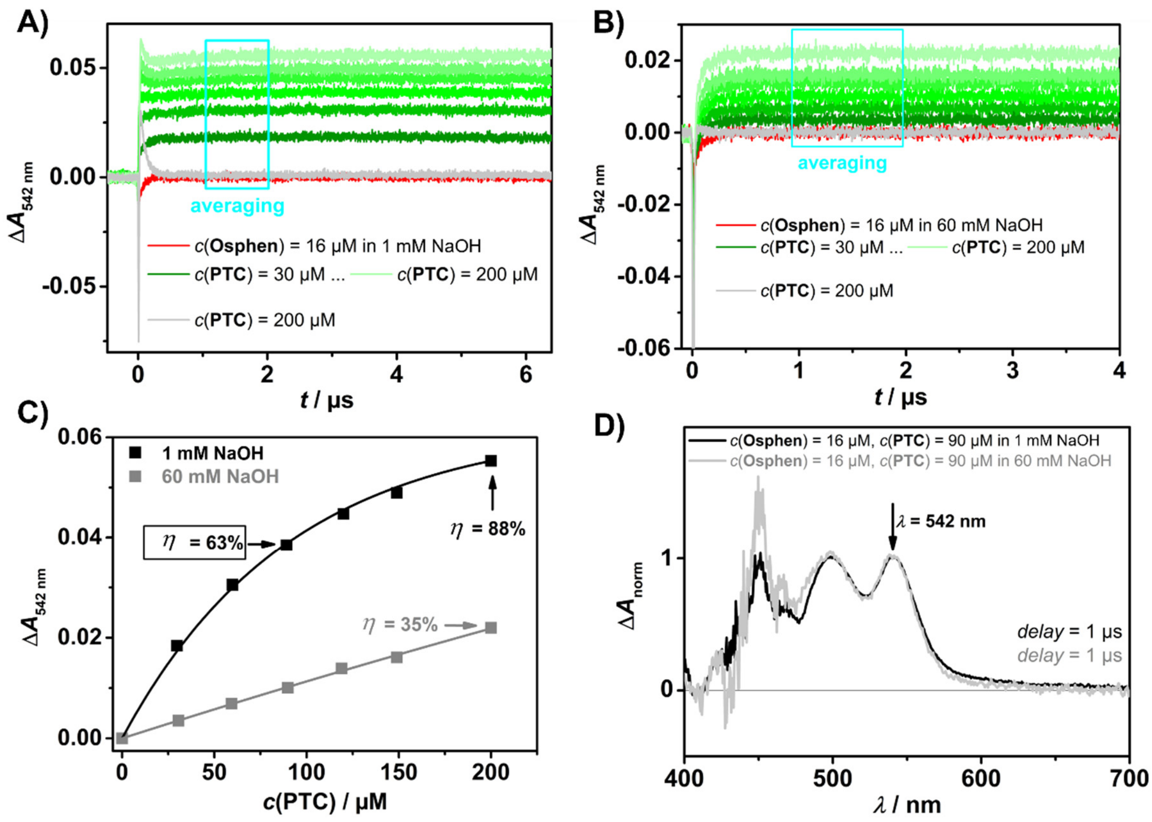

Figure S18 (A) Time-resolved absorption of Ar-saturated aqueous solutions (1 mM NaOH) containing **Osphen** and **PTC** at different concentrations of **PTC** at λ_{det} = 542 nm after laser excitation (λ_{exc} = 532 nm). The cyan boxes indicate the time range in which the signal was averaged for the plot in C). (B) Time-resolved absorption of similar solutions in 60 mM NaOH. (C) *c*(**PTC**) dependent averaged values for $\Delta A_{542\ nm}$ in water containing 1mM or 60 mM NaOH. (D) Normalized transient absorption spectra of solutions containing 16 µM **Osphen** and 90 µM **PTC** in water containing 1mM or 60 mM NaOH.

By increasing the concentration of NaOH, the ionic strength of the solution is increased. This salt effect[19] suppresses the ground state association between **Osphen** and **PTC** leading to a decrease in the efficiency of ^{3}**PTC** formation by a factor of 2.5. The efficiency of dynamic quenching is decreased due to the kinetic salt effect as well. This estimation is based on the

reasonable assumption that the difference molar absorption coefficient of **³PTC** at 542 nm is the same in 1 mM NaOH and 60 mM NaOH.

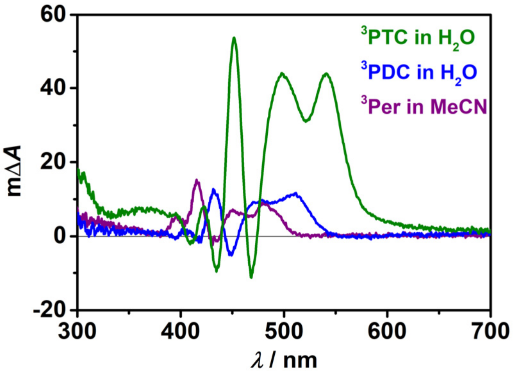

Figure S19 Comparison of the transient absorption spectra of the triplet states of **PTC**, **PDC** and **Per** after laser excitation (λ_{exc} = 532 nm, *delay* 1 µs) in water (1 mM NaOH) or acetonitrile in the presence of **Osphen** (c(**Osphen**) = 16 µM, Cl salt in aqueous solution and PF_6 salt in acetonitrile, concentration of perylene derivatives: 30 µM).

S7 $^1O_2$ assay

Kinetic simulation

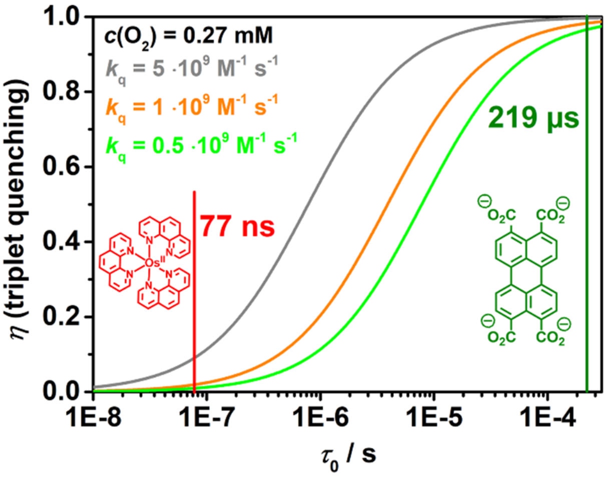

Figure S20 Kinetic simulation of the quenching efficiency of the triplet states **³Osphen** or **³PTC** through oxygen in air-saturated water (c = 0.27 mM)[20] with different quenching rate constants utilizing the well-known kinetic equations[14,20,21].

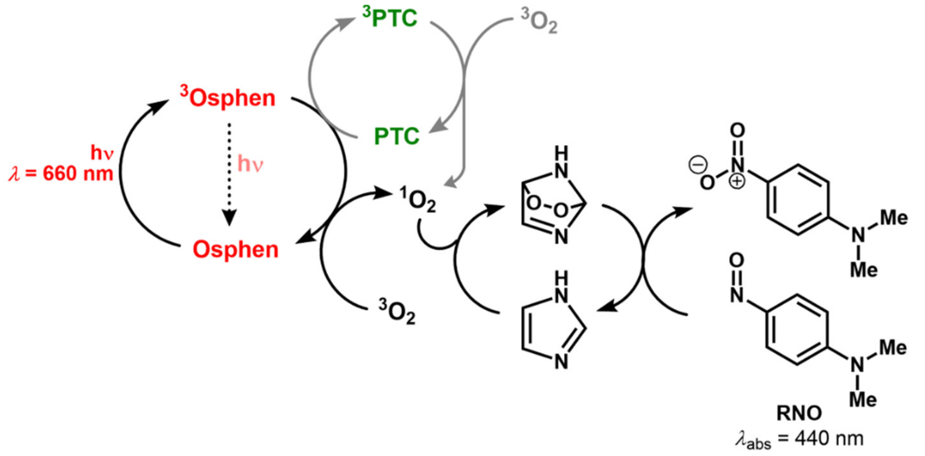

Figure S21 Mechanism of the singlet oxygen production with **Osphen** (and **PTC**) followed by the singlet oxygen assay developed by Kraljić and Mohsni.[22]

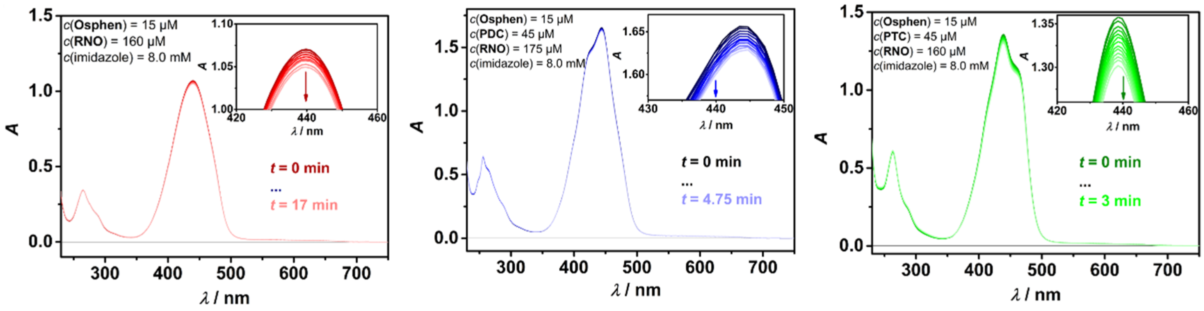

Figure S22 UV-Vis spectra after different irradiation times (660 nm LED) in aqueous solution (1 mM NaOH) in the context of the $^1O_2$ assay (see Figure 4 C in the main part). Concentrations of key species are given in the figure.

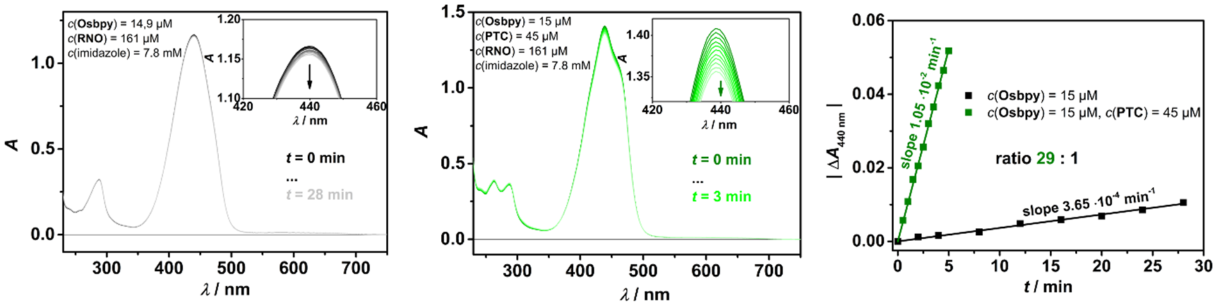

Figure S23 (left, middle) UV-Vis spectra after different irradiation times (660 nm LED) in aqueous solution (1 mM NaOH) in the context of the $^1O_2$ assay. (right) Results of a $^1O_2$ assay displaying different $^1O_2$ formation rates for an aqueous solution containing **Osbpy** with or without **PTC**. Concentrations of key species are given in the figure.

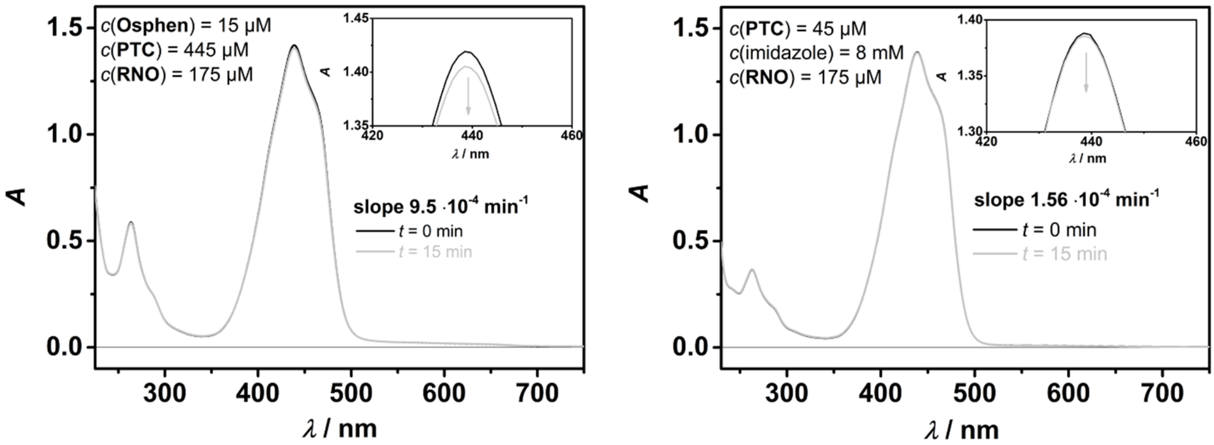

Figure S24 Control experiments for the singlet oxygen assay without imidazole (left) or without **Osphen** (right) in aqueous solution (1 mM NaOH).

Without imidazole, there is a minimal decrease in the absorption band at 440 nm. This can be explained by a minor oxidation of the **RNO** compound due to the large excess of singlet oxygen in the presence of **PTC**. On the other hand, a slight decrease in absorbance at this wavelength may be caused by a small degradation of **PTC** by singlet oxygen. However, the observed decrease at 440 nm, expressed by a slope of $9.5 \cdot 10^{-4}$ min^{-1}, is more than one order of magnitude smaller than the decrease observed in the presence of imidazole ($18.7 \cdot 10^{-3}$ min^{-1}, see Figure 4 in the main part), indicating that these side reactions are of very low importance compared to the main mechanism shown in Figure S21.

S8 Cyclic voltammetry

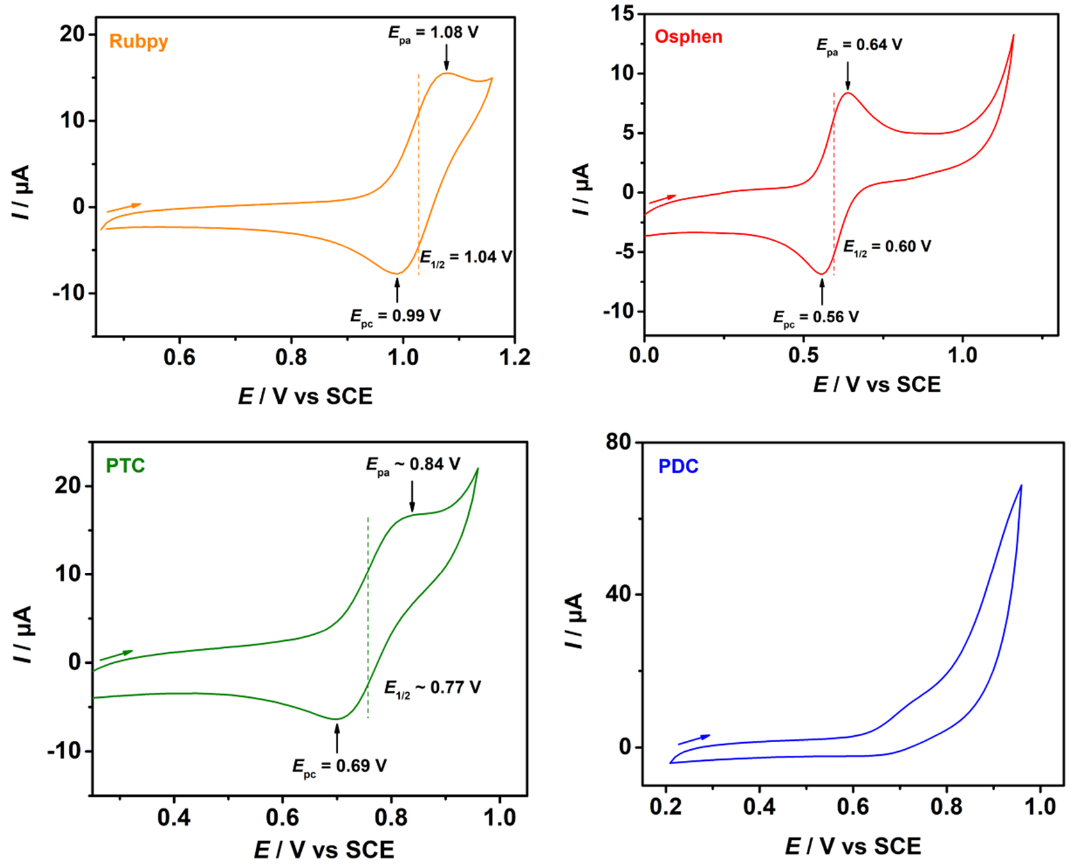

Figure S25 Cyclic voltammograms of aqueous solutions containing 0.5 mM of **Rubpy**, **Osphen**, **PTC** or **PDC** in 0.1 M Na_2HPO_4 (pH = 10.1) with a scan rate of 2 V/s. The arrows indicate the direction of the potential scan.

Cyclic voltammetry measurements were carried out to determine or estimate the oxidation potentials of **Osphen**, **PTC** and **PDC** in the ground state in water (which is a challenging solvent for electrochemical analysis). First, the oxidation potential (half-wave potential $E_{1/2}$) of the well-known photocatalyst **Rubpy** was recorded in water (1.04 V vs SCE, see Figure S25), which is the same as described in literature.[23] The measured $E_{1/2}$ of **Osphen** in water (0.60 V vs SCE) is slightly lower than that in MeCN (0.44 V vs Fc/Fc^+, 0.86 V vs SCE).[24] For **PTC**, $E_{1/2}$ was determined to be 0.77 V vs SCE, which is similar to the known oxidation potential of unsubstituted perylene in MeCN (0.85 V vs SCE).[25] However, for **PDC**, no reliable oxidation potential could be measured under the chosen conditions. Since the potential of **PTC** is similar to that of unsubstituted perylene in MeCN, it is assumed that the carboxylate groups do not have a pronounced influence on $E_{1/2}$ and, therefore, the same value is assumed for **PDC** as for **PTC**. The excited state oxidation potentials ($E_{1/2}$*) of the triplet state of **Osphen**, **PTC** and **PDC** were then calculated based on $E_{1/2}$ and the triplet state energy (see Table S4).

The reduction of molecular oxygen O_2 to the superoxide radical anion ($O_2^{\bullet-}$) takes place at a potential of -0.33 V vs NHE (-0.58 V vs SCE).[26,27] This makes the reduction of molecular oxygen by ^{3}**Osphen** ($E_{1/2}$* = -1.20 V vs SCE) an exergonic event, which explains the relatively low estimated singlet oxygen quantum yield by this photocatalyst as electron transfer seems to be a prominent quenching pathway. On the other hand, the reduction of O_2 by ^{3}**PTC** ($E_{1/2}$* = -0.53 V vs SCE) is slightly endergonic, which should drastically slow down the photoinduced electron transfer and favor Dexter energy transfer process during the quenching event of ^{3}**PTC**

by molecular oxygen. The slightly more energetic triplet state ^{3}**PDC** ($E_{1/2}$* = -0.64 V vs SCE) makes O_2 reduction again an exergonic process, decreasing the singlet oxygen quantum yield, which is in line with experimental observations of the singlet oxygen assay (see main part). In other words, although the oxidation potentials of the triplet states of **PTC** and **PDC** are very close, they fall in a range where the reduction of O_2 changes from slightly endergonic (for ^{3}**PTC**) to slightly exergonic (for ^{3}**PDC**), whereby a small change in potential results in a large change in the rate constant of electron transfer, which is noticeably reflected in the altered singlet oxygen quantum yield.

Table S4: Measured half-wave oxidation potentials of the ground state ($E_{1/2}$) and calculated oxidation potentials of the triplet excited state ($E_{1/2}$*) of **Osphen**, **PTC** and **PDC** using the triplet state energy (E_T).

Compound	$E_{1/2}$ / V vs SCE	E_T / eV	$E_{1/2}$* / V vs SCE
**Osphen**	0.60[a]	1.80[b]	-1.20
**PTC**	0.77[a]	1.30[c]	-0.53
**PDC**	0.77[d]	1.41[c]	-0.64

[a] Measured in this work. [b] From ref. [8]. [c] Determined by DFT (B3LYP/def2-SVP level of theory). [d] The same ground-state potential is assumed as for **PTC**.

S9 Photooxygenations

S9.1 Photooxygenation of **5-HMF** (**1**)

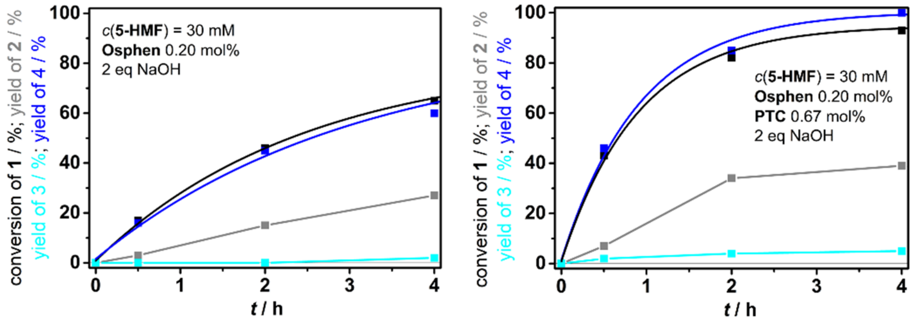

Figure S26 Time-resolved formation of products for the photooxygenation (660 nm LED) of **5-HMF** with **Osphen** in the absence (left) or in the presence of **PTC** (right) in air-saturated 60 mM NaOH.

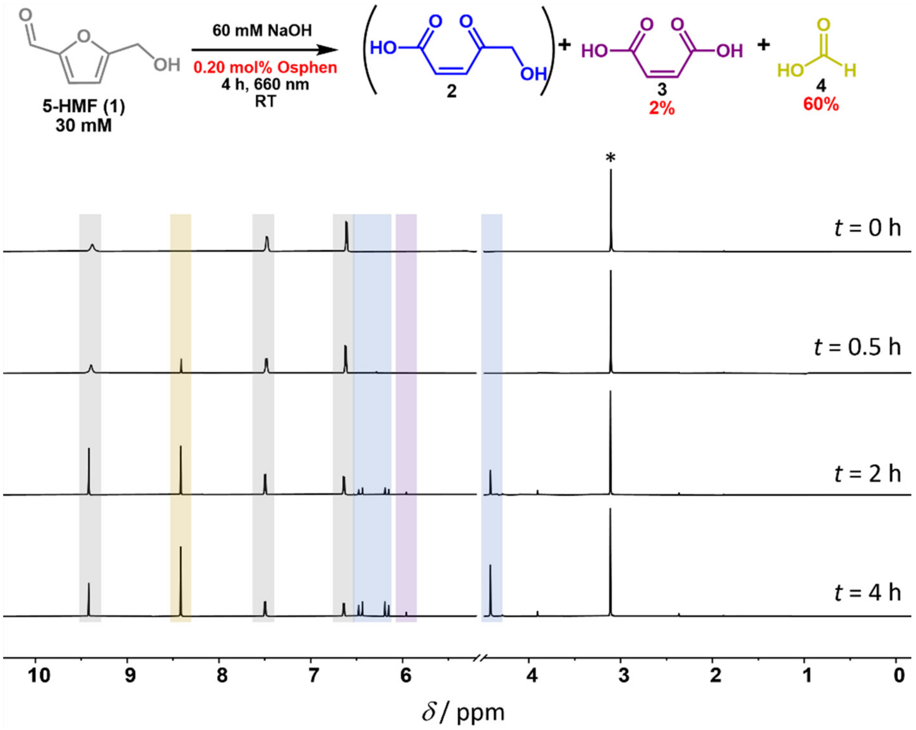

Figure S27 Reaction equation and ^{1}H NMR spectra of the photooxygenation of **5-HMF** with **Osphen** after different irradiation times (660 nm LED) without **PTC** along with signal assignments. The signal marked with an asterisk corresponds to the internal standard dimethyl sulfone with $c \sim 5$ mM. Signal assignment according to reference [28].

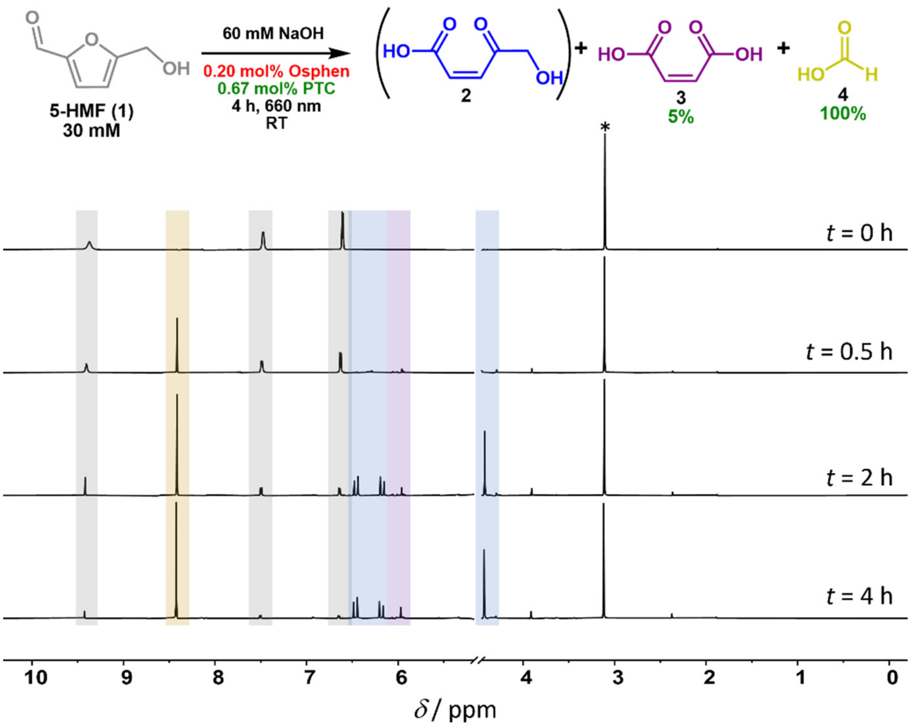

Figure S28 Reaction equation and ^{1}H NMR spectra of the photooxygenation of **5-HMF** with **Osphen** after different irradiation times (660 nm LED) in the presence of **PTC** along with signal assignments. The signal marked with an asterisk corresponds to the internal standard dimethyl sulfone with $c \sim 5$ mM.

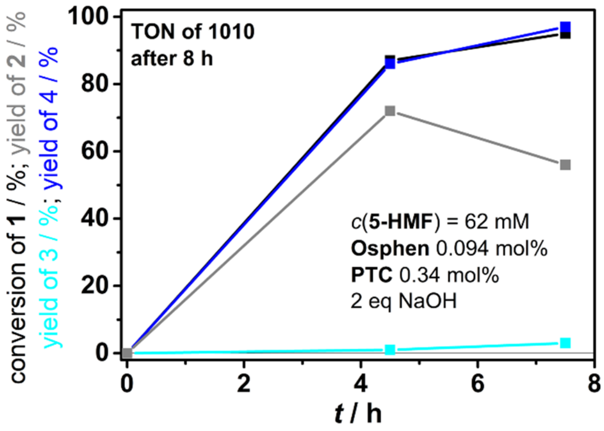

Figure S29 Time-resolved formation of products for the photooxygenation (660 nm LED) of **5-HMF** with **Osphen** in the presence of **PTC** with a higher substrate loading in 120 mM NaOH.

Table S5: Control experiments for the photooxygenation of **5-HMF** with **Osphen** and **PTC** in air-saturated 60 mM NaOH using red light (660 nm LED).

c(**5-HMF**) (1)	**Osphen**	**PTC**	irradiation	air-/Ar-saturated	conversion of **1**	formation of **4**
30 mM	-	0.67 mol%	660 nm LED	air-sat.	5 %	0%
30 mM	0.20 mol%	0.67 mol%	-	air-sat.	5 %	0%
30 mM	0.20 mol%	0.67 mol%	660 nm LED	Ar-sat.	5 %	0%

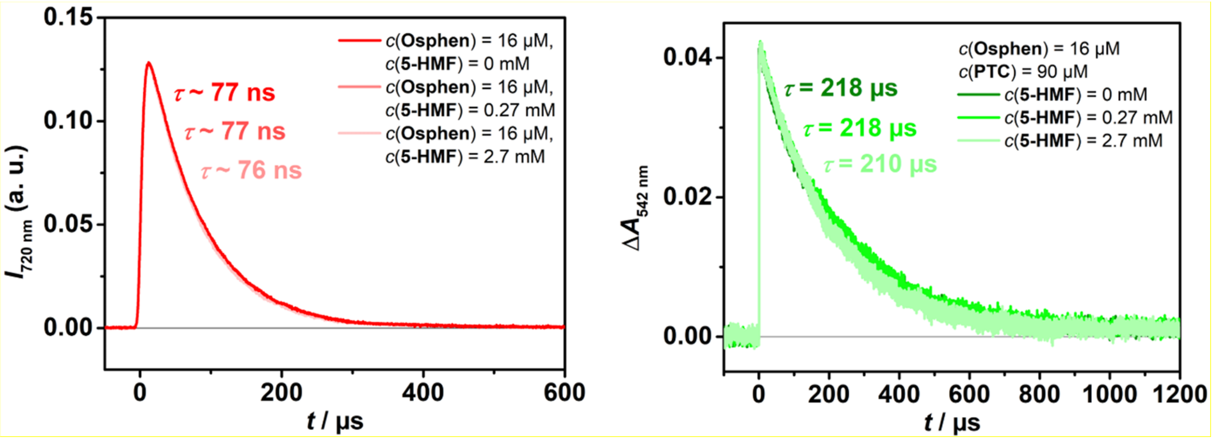

Figure S30 (left) Time-resolved emission traces of a solution containing **Osphen** with or without **5-HMF** in Ar-saturated 1 mM NaOH after laser excitation (λ_{exc} = 532 nm, λ_{det} = 720 nm). (right) Time-resolved absorption traces of a solution containing **Osphen** and **PTC** observing ^{3}**PTC** with or without **5-HMF** in Ar-saturated 1 mM NaOH after laser excitation (λ_{exc} = 532 nm, λ_{det} = 542 nm).

In a further control experiment, a potential direct reaction of **5-HMF** with ^{3}**Osphen** or ^{3}**PTC** was investigated (see Figure S30). Here, **5-HMF** was used in the same concentration as dissolved oxygen in air-saturated water and virtually no quenching of the triplet states of **Osphen** or **PTC**

by **5-HMF** was observed. Even after the concentration of **5-HMF** was increased by an order of magnitude, only very minor quenching can be observed, which is in clear contrast to the highly efficient quenching by molecular oxygen, especially for ^{3}**PTC** (compare to Figure 4 B in the main part).

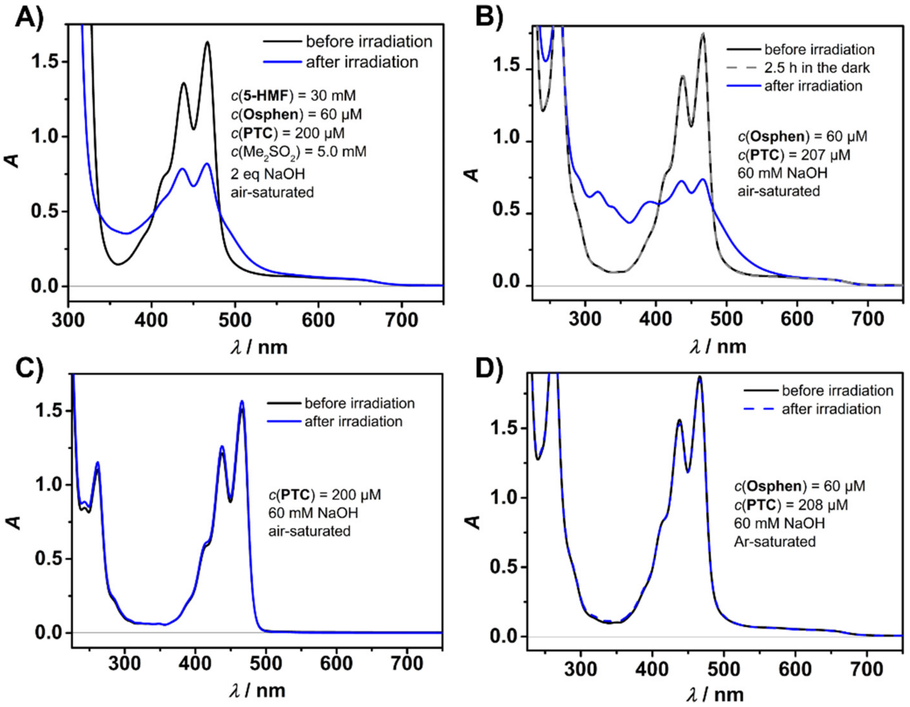

Figure S31 (A) UV-Vis spectra before (black) and after 2.5 h of irradiation (blue) with the 660 nm LED of the air-saturated reaction solution used for the photooxygenation of **5-HMF** (recorded in 2 mm cuvettes). (B) UV-Vis spectra before (black) and after 2.5 h of irradiation (blue) with the 660 nm LED of the same solution as in A) without **5-HMF** and the internal standard dimethyl sulfone. (C) UV-Vis spectra before (black) and after 2.5 h of irradiation (blue) with the 660 nm LED of the same solution as in A) without **5-HMF**, **Osphen** and the internal standard dimethyl sulfone. (D) UV-Vis spectra before (black) and after 2.5 h of irradiation (blue) with the 660 nm LED of the same solution as in A) without **5-HMF**, oxygen and the internal standard dimethyl sulfone.

The UV-Vis spectrum of the reaction solution for the photooxygenation of **5-HMF** with **Osphen** and **PTC** is clearly different after irradiation to the spectrum before irradiation. Particularly striking is the decrease in absorbance at the absorption maximum of **PTC**, which indicates a decomposition of the perylene salt (see Figure S31 A). A similar decrease can also be observed in the absence of the substrate and the internal standard (see Figure S31 B). In contrast, the absorption band of **Osphen** around 650 nm remains unchanged, indicating a high stability of the osmium complex under these conditions. Previously, the high stability of osmium complexes under irradiation conditions has been demonstrated.[29] Without sensitizer or in the absence of oxygen, no decomposition of **PTC** takes place (see Figure S31 C and D). This indicates that **PTC** reacts with the singlet oxygen formed. A reaction of perylenes with singlet oxygen is already literature-known.[30] A reaction of **PTC** with singlet oxygen is also supported by the observation that the decomposition of **PTC** proceeds less slowly in the presence of **5-**

HMF, which in turn also reacts with singlet oxygen. It is assumed that the decomposition of **PTC** progresses significantly towards the end of the reaction when there is little substrate left. The degradation of **PTC** could therefore be avoided when a high concentration of the substrate is present. It is important to emphasize that with **PTC** only the much cheaper part of the catalyst system is decomposed and the expensive sensitizer **Osphen** is hardly affected, which in principle can be recovered and reused. To demonstrate that the sensitizer **Osphen** remains intact, it was shown that the turnover frequency could be significantly increased again after 2.5 h of irradiation by adding **PTC** (see Figure S32). For this purpose, the loading of **Osphen** was reduced.

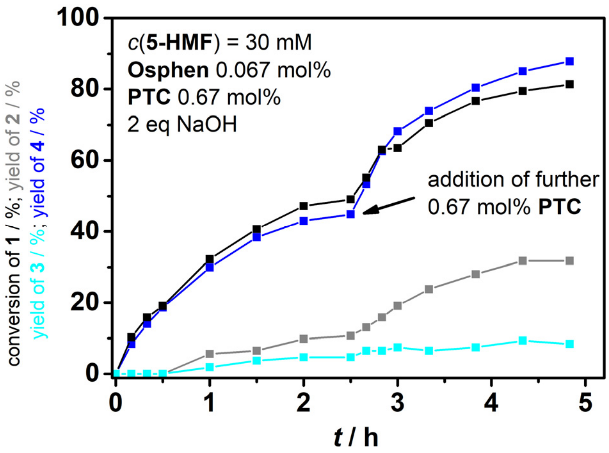

Figure S32 Time-resolved formation of products and conversion for the photooxygenation (660 nm LED) of **5-HMF** with **Osphen** in the presence of **PTC** in air-saturated 60 mM NaOH. After 2.5 h, further 0.67 mol% of **PTC** were added to reactivate the catalytic system, which opperates more effectively with a Coulombic dyad compared to the Os complex alone.

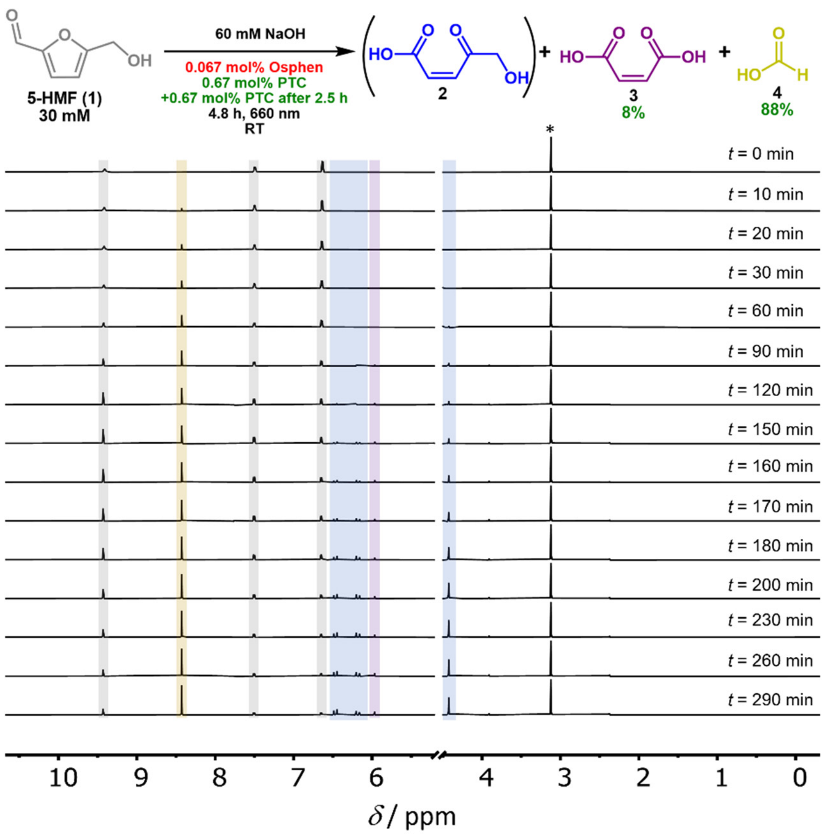

Figure S33 Reaction equation and ^{1}H NMR spectra of the photooxygenation of **5-HMF** with **Osphen** after different irradiation times (660 nm LED) in the presence of **PTC** along with signal assignments. The signal marked with an asterisk corresponds to the internal standard dimethyl sulfone with $c \sim 5$ mM.

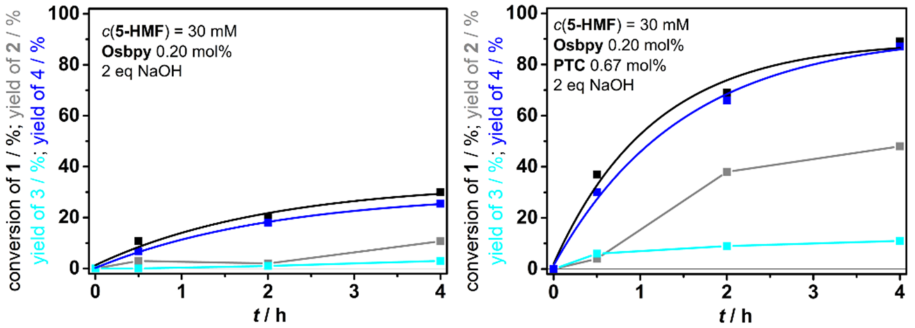

Figure S34 Time-resolved formation of products for the photooxygenation (660 nm LED) of **5-HMF** with **Osbpy** in the absence (left) or in the presence of **PTC** (right) in air-saturated 60 mM NaOH.

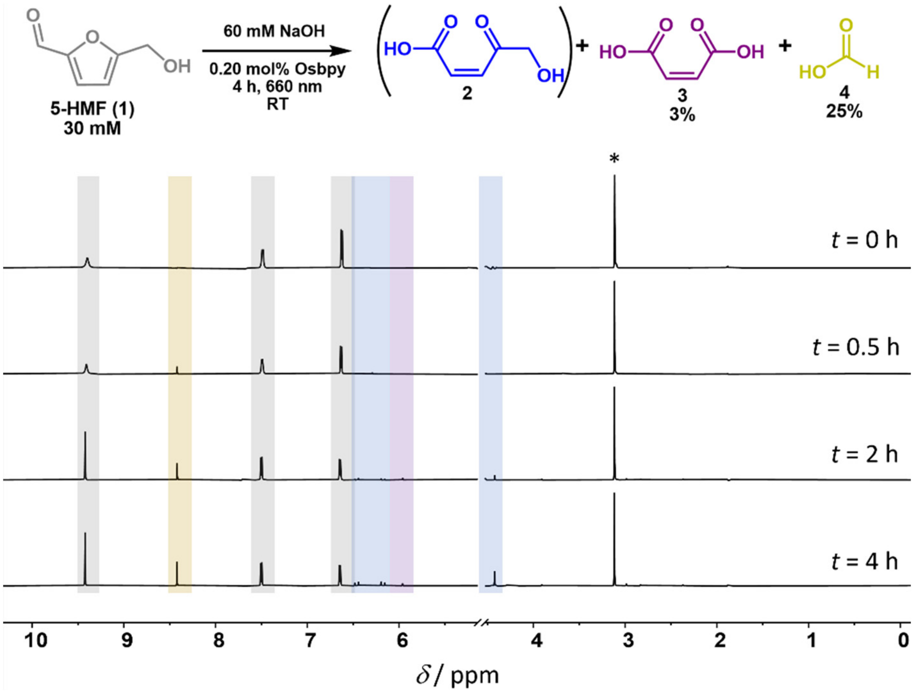

Figure S35 Reaction equation and ^{1}H NMR spectra of the photooxygenation of **5-HMF** with **Osbpy** after different irradiation times (660 nm LED) without **PTC** along with signal assignments. The signal marked with an asterisk corresponds to the internal standard dimethyl sulfone with $c \sim 5$ mM.

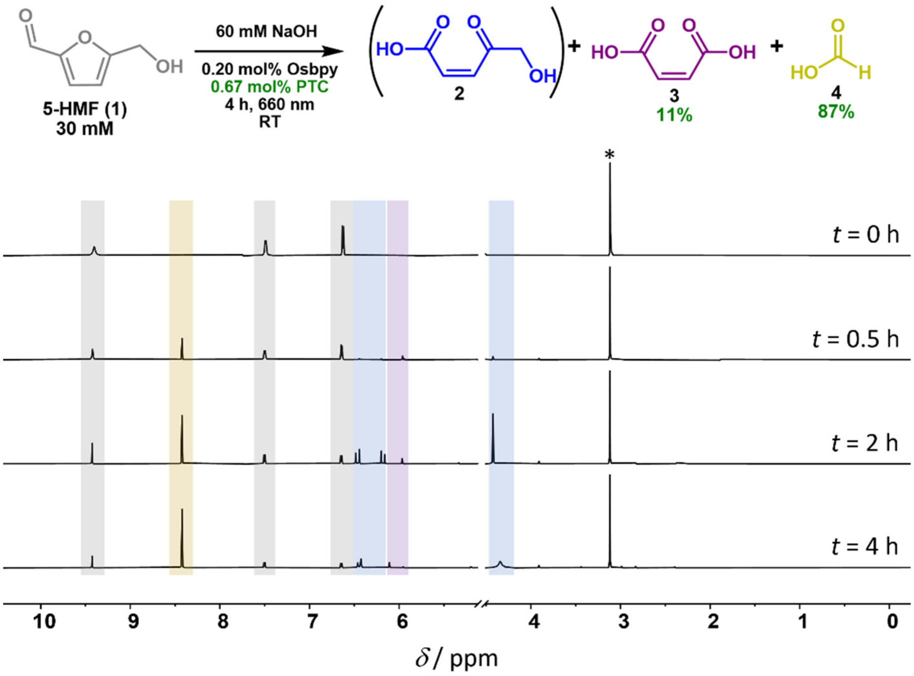

Figure S36 Reaction equation and ^{1}H NMR spectra of the photooxygenation of **5-HMF** with **Osbpy** after different irradiation times (660 nm LED) in the presence of **PTC** along with signal assignments. The signal marked with an asterisk corresponds to the internal standard dimethyl sulfone with $c \sim 5$ mM.

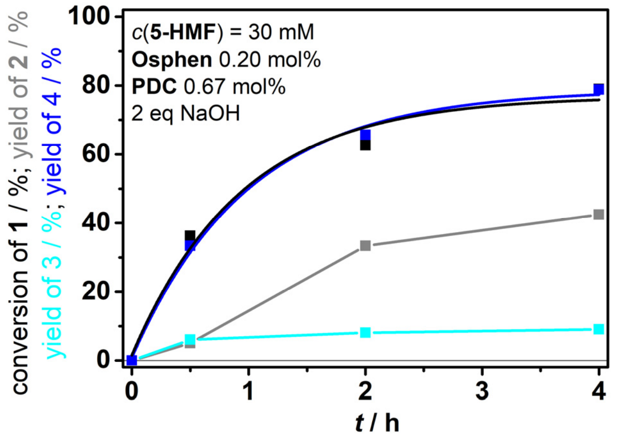

Figure S37 Time-resolved formation of products for the photooxygenation (660 nm LED) of **5-HMF** with **Osphen** in the presence of **PDC** in air-saturated 60 mM NaOH. Results for related experiments with **PTC** are shown in Figure S26 (right).

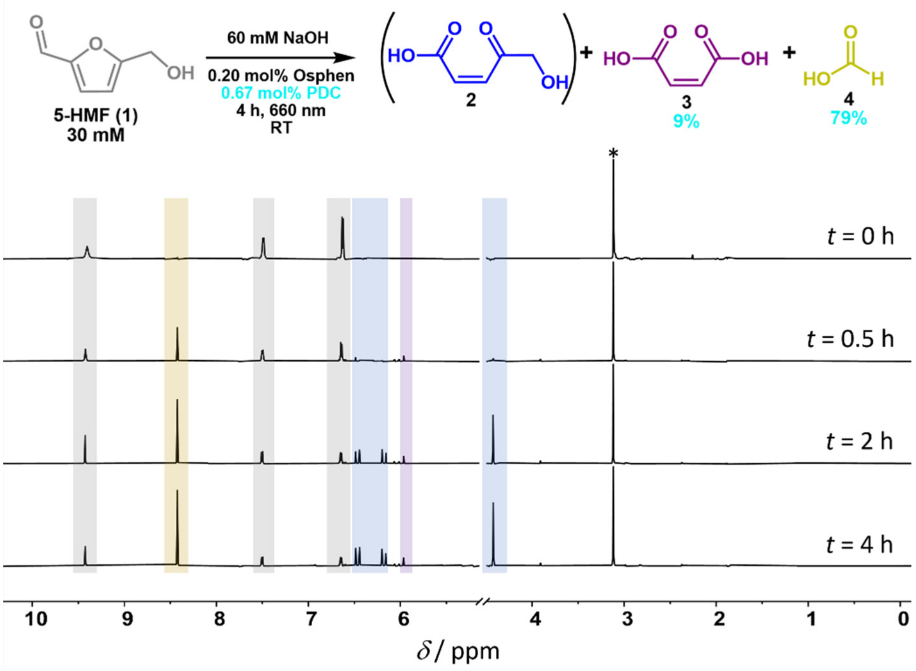

Figure S38 Reaction equation and ^{1}H NMR spectra of the photooxygenation of **5-HMF** with **Osphen** after different irradiation times (660 nm LED) in the presence of **PDC** along with signal assignments. The signal marked with an asterisk corresponds to the internal standard dimethyl sulfone with $c \sim 5$ mM.

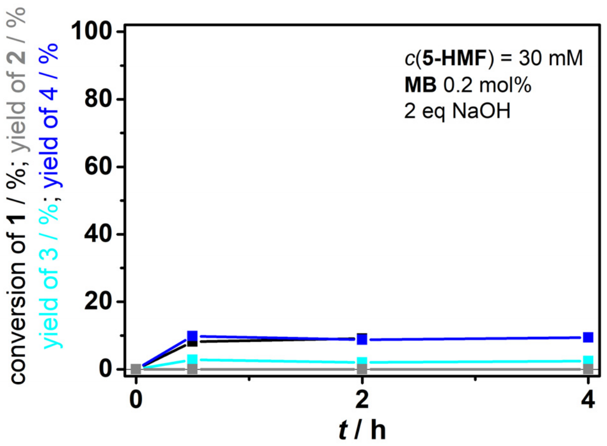

Figure S39 Time-resolved formation of products for the photooxygenation (660 nm LED) of **5-HMF** with methylene blue (**MB**) in air-saturated 60 mM NaOH.

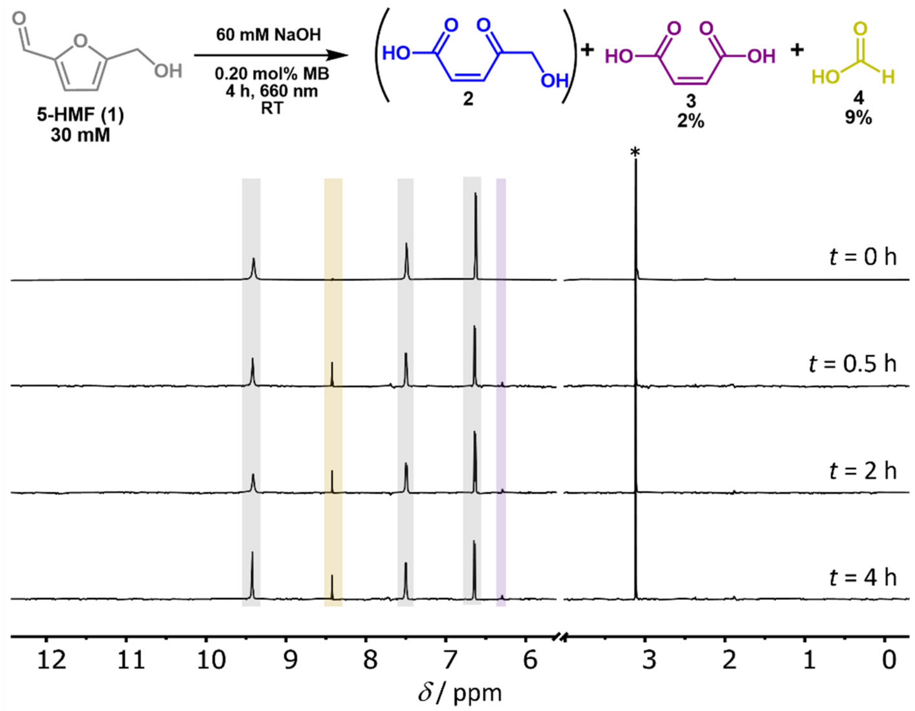

Figure S40 Reaction equation and ^{1}H NMR spectra of the photooxygenation of **5-HMF** with **MB** after different irradiation times (660 nm LED) along with signal assignments. The signal marked with an asterisk corresponds to the internal standard dimethyl sulfone with $c \sim 5$ mM.

S9.2 Photooxygenation of **5**

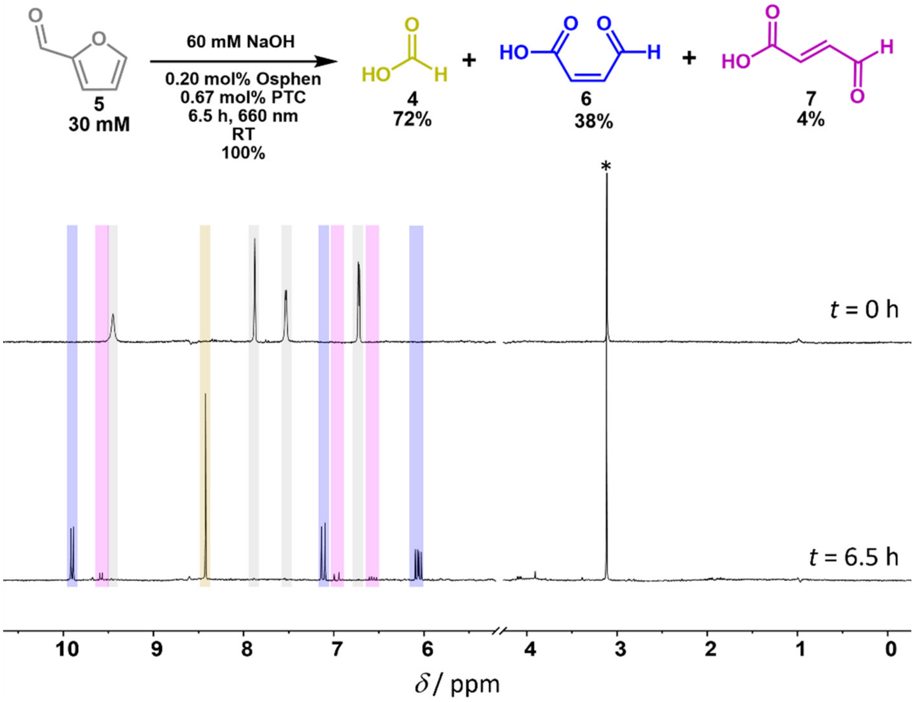

Figure S41 Reaction equation and ^{1}H NMR spectra of the photooxygenation of **5** before and after the irradiation (660 nm LED) with **Osphen** in the presence of **PTC** at room temperature along with signal assignments. The signal marked with an asterisk corresponds to the internal standard dimethyl sulfone with *c* ~ 5 mM. Signal assignment according to references [31,32].

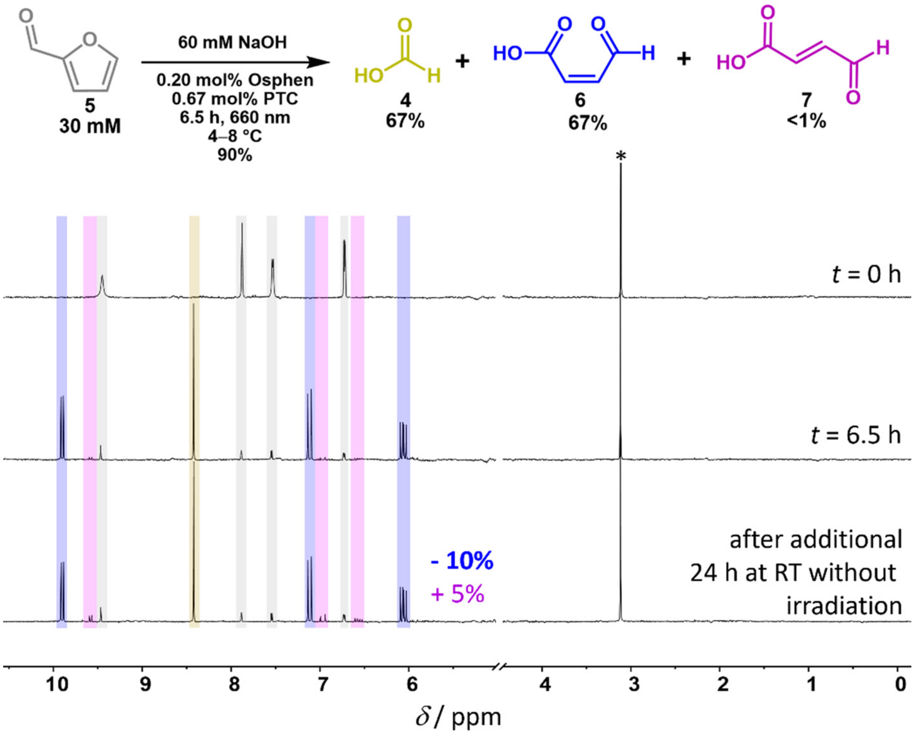

Figure S42 Reaction equation and ^{1}H NMR spectra of the photooxygenation of **5** before and after the irradiation (660 nm LED) at 4–8 °C with **Osphen** in the presence of **PTC** and after standing for additional 24 h in the dark at room temperature (RT) along with signal assignments. The signal marked with an asterisk corresponds to the internal standard dimethyl sulfone with *c* ~ 5 mM.

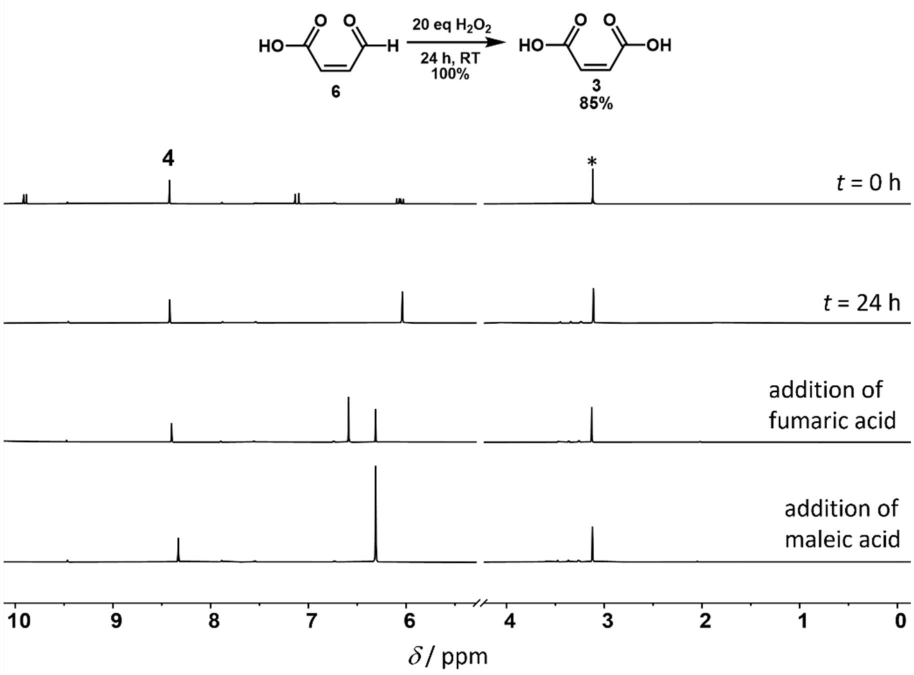

Figure S43 Reaction equation and ^{1}H NMR spectra of the oxidation of **6** to **3** before and after 24 h at room temperature in the dark and after the addition of about 1 eq of fumaric acid or maleic acid as reference. The signal marked with an asterisk corresponds to the internal standard dimethyl sulfone with c ~ 5 mM.

S9.3 Photooxygenation of **8**

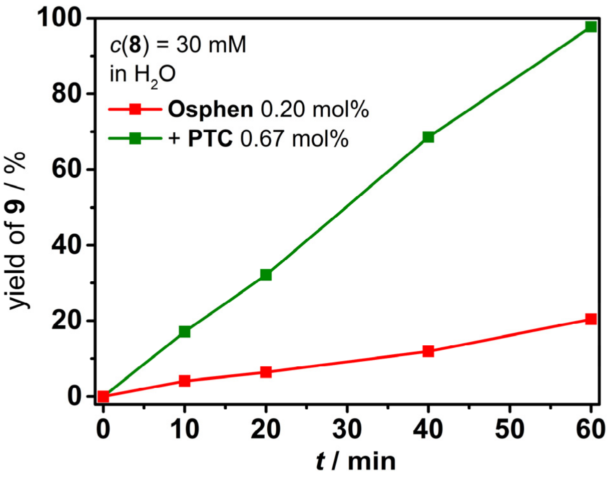

Figure S44 Time-resolved product formation for the photooxygenation (660 nm LED) of **8** with **Osphen** in the absence or in the presence of **PTC** in air-saturated aqueous solution (1 mM NaOH).

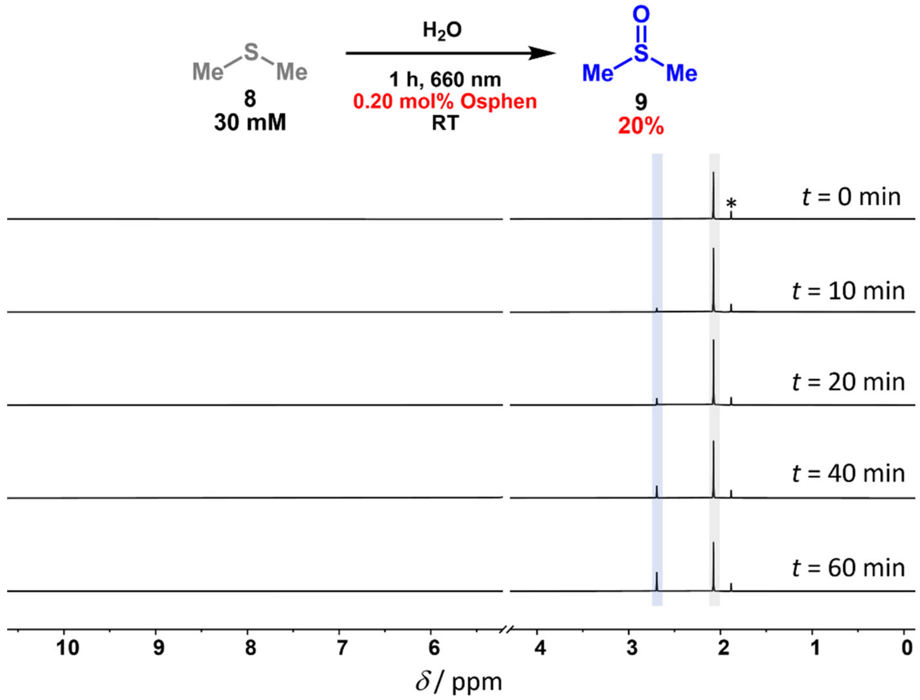

Figure S45 Reaction equation and ^{1}H NMR spectra of the photooxygenation of **8** in aqueous solution (1 mM NaOH) after different irradiation times (660 nm LED) utilizing **Osphen** without **PTC** along with signal assignments. The signal marked with an asterisk corresponds to the internal standard sodium acetate with *c* ~ 5 mM. Signal assignment according to reference [3].

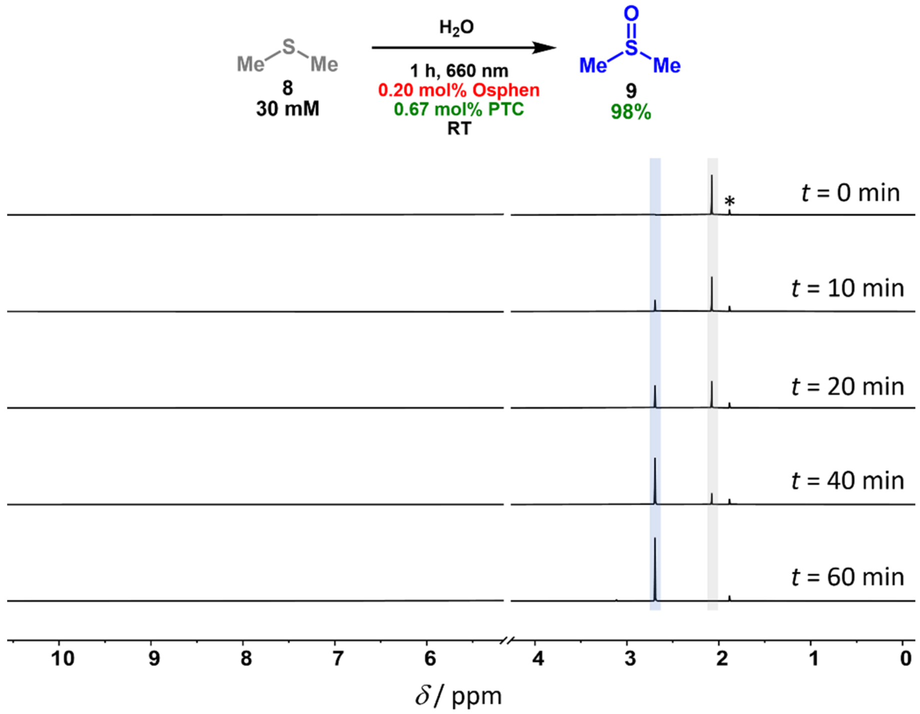

Figure S46 Reaction equation and ^{1}H NMR spectra of the photooxygenation of **8** in aqueous solution (1 mM NaOH) after different irradiation times (660 nm LED) utilizing **Osphen** with **PTC** along with signal assignments. The signal marked with an asterisk corresponds to the internal standard sodium acetate with *c* ~ 5 mM.

S9.4 Degradation of **10**

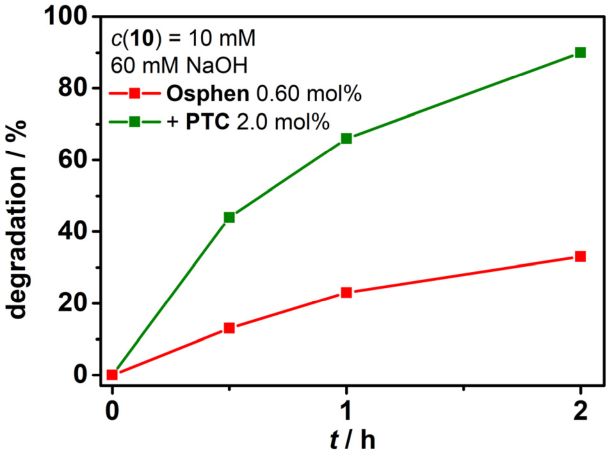

Figure S47 Time-resolved degradation of **10** via $^1O_2$ under irradiation (660 nm LED) with **Osphen** in the absence or in the presence of **PTC** in air-saturated aqueous solution (60 mM NaOH).

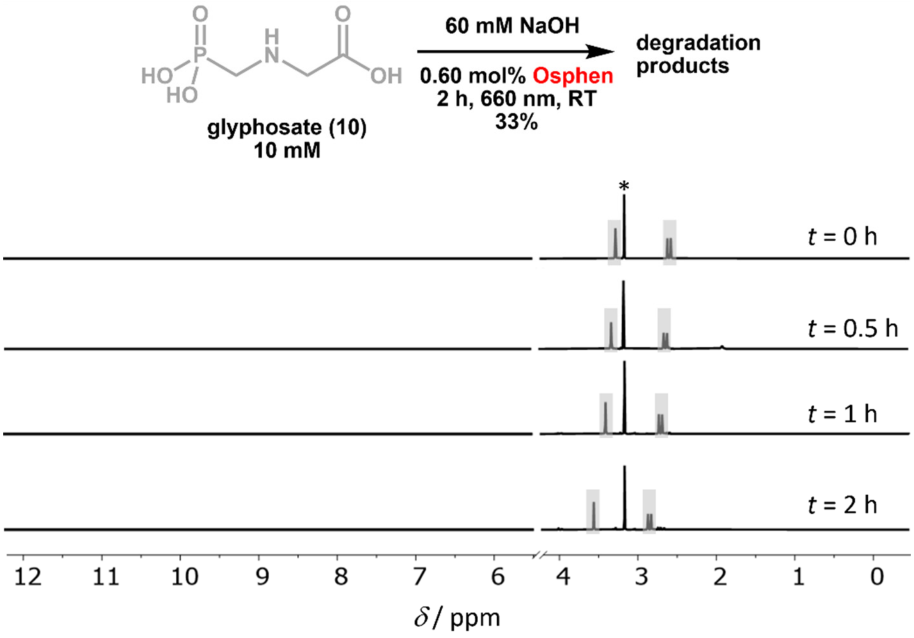

Figure S48 Reaction equation and ^1H NMR spectra of the degradation of **10** via $^1O_2$ generation in aqueous solution (60 mM NaOH) after different irradiation times (660 nm LED) utilizing **Osphen**. The signal marked with an asterisk corresponds to the internal standard dimethyl sulfone with $c \sim 4$ mM.

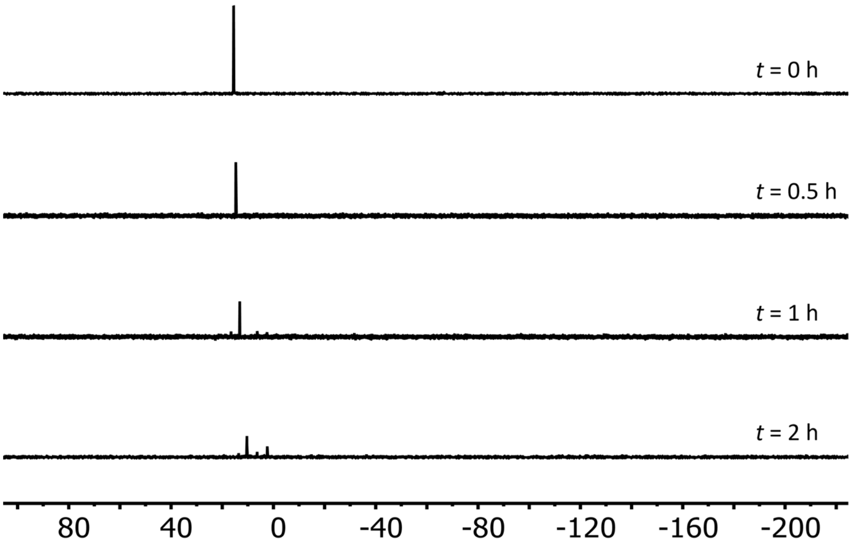

Figure S49 ^{31}P NMR spectra of the degradation of **10** via $^{1}O_2$ generation in aqueous solution (60 mM NaOH) after different irradiation times (660 nm LED) utilizing **Osphen**. See also Figure S48 for corresponding ^{1}H NMR spectra.

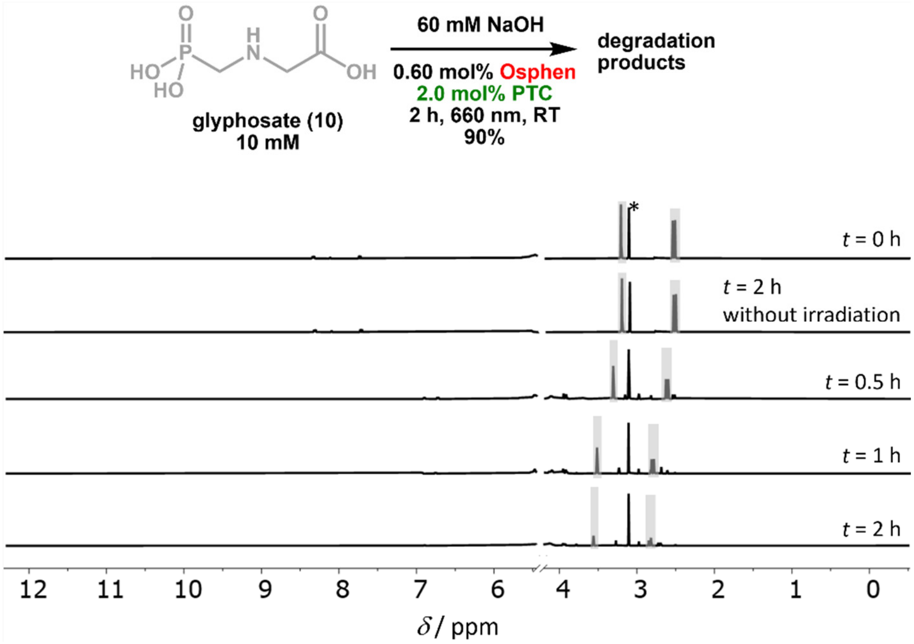

Figure S50 Reaction equation and ^{1}H NMR spectra of the degradation of **10** via $^{1}O_2$ generation in aqueous solution (60 mM NaOH) after different irradiation times (660 nm LED) utilizing **Osphen** and **PTC**. The signal marked with an asterisk corresponds to the internal standard dimethyl sulfone with $c \sim 3$ mM.

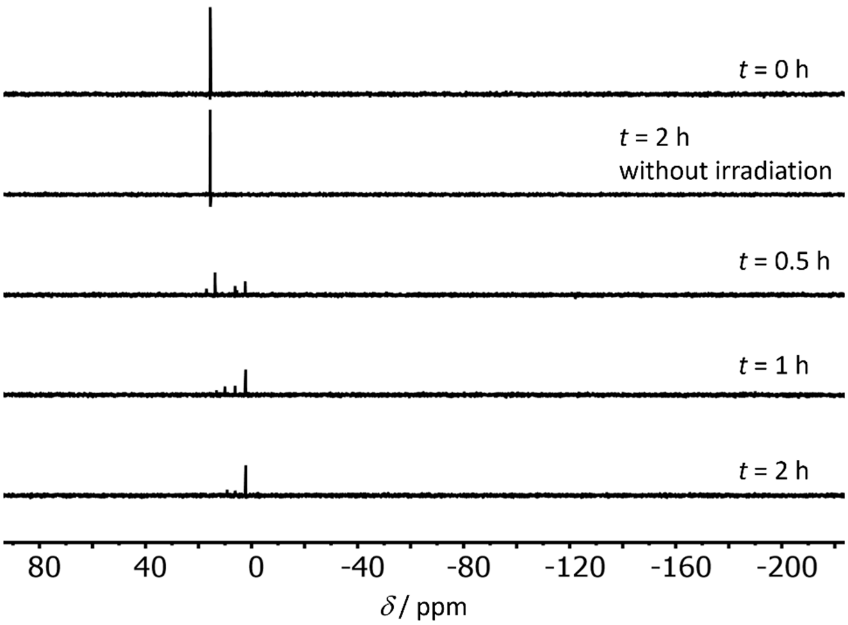

Figure S51 ^{31}P NMR spectra of the degradation of **10** via $^{1}O_2$ generation in aqueous solution (60 mM NaOH) after different irradiation times (660 nm LED) utilizing **Osphen** and **PTC**. See also Figure S50 for corresponding ^{1}H NMR spectra.

S9.5 Photooxygenation of **11**

The solvent mixture MeOH/H_2O 10/1 was identified as ideal, as **11** or the dyad system precipitated out of the solution when the water content is increased or decreased, respectively.

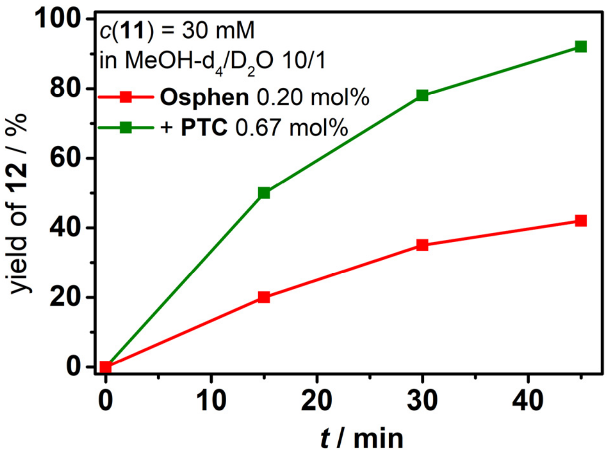

Figure S52 Time-resolved product formation for the photooxygenation (660 nm LED) of **11** with **Osphen** in the absence or in the presence of **PTC** in MeOH-d_4/D_2O under air.

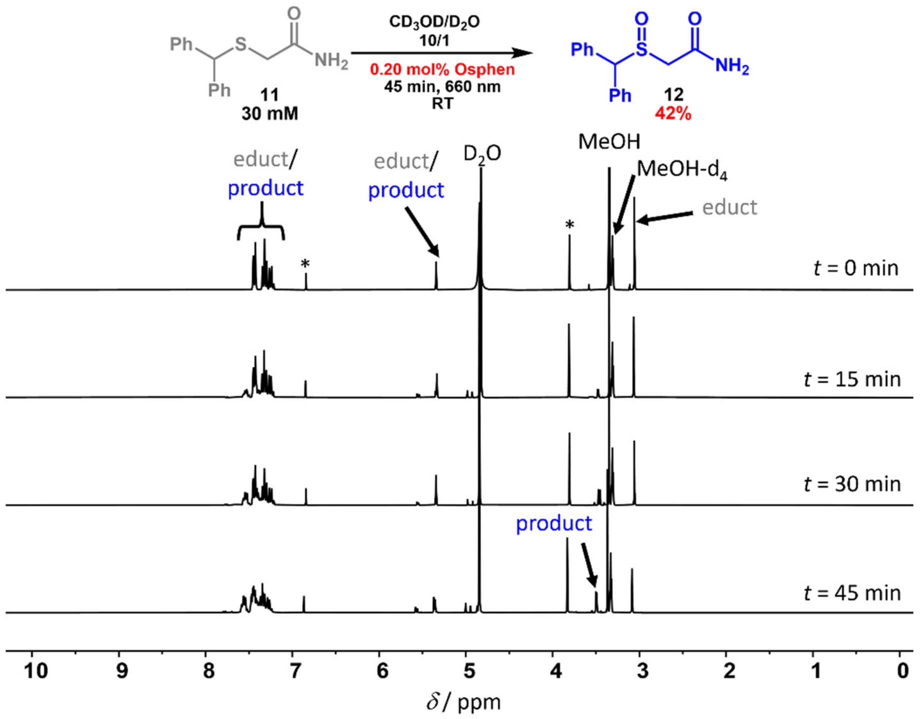

Figure S53 Reaction equation and ^{1}H NMR spectra of the photooxygenation of **11** after different irradiation times (660 nm LED) utilizing **Osphen** without **PTC** along with signal assignments. The signal marked with an asterisk corresponds to the internal standard dimethyl fumarate with $c \sim 30$ mM. Signal assignment according to the reference spectrum in Figure S71.

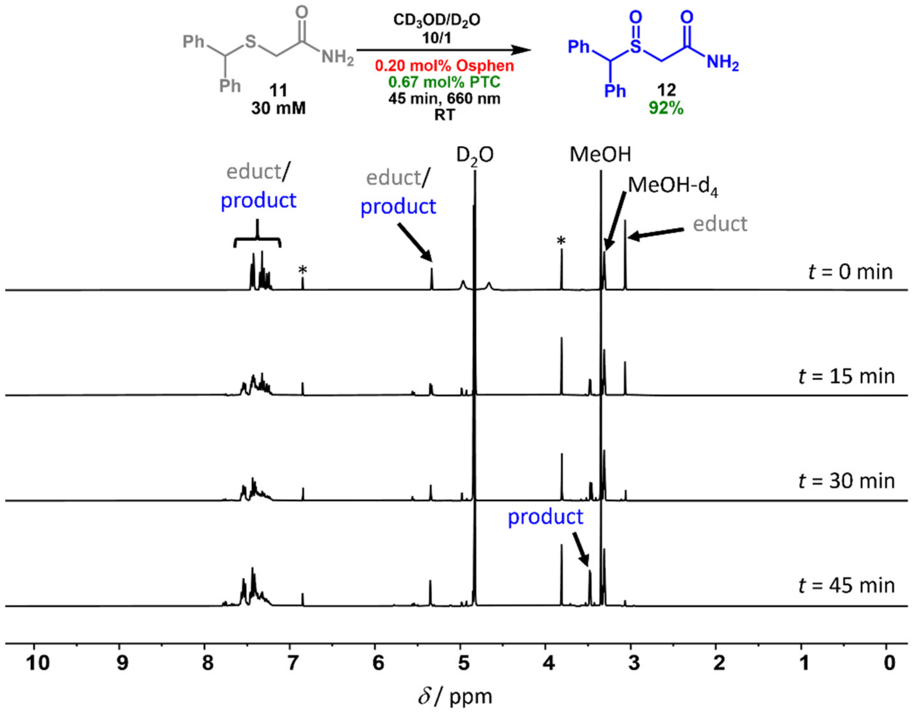

Figure S54 Reaction equation and ^{1}H NMR spectra of the photooxygenation of **11** after different irradiation times (660 nm LED) with **Osphen** in the presence of **PTC** along with signal assignments. The signal marked with an asterisk corresponds to the internal standard dimethyl fumarate with $c \sim 30$ mM.

S9.5.1 Spectroscopic investigations in MeOH/H_2O

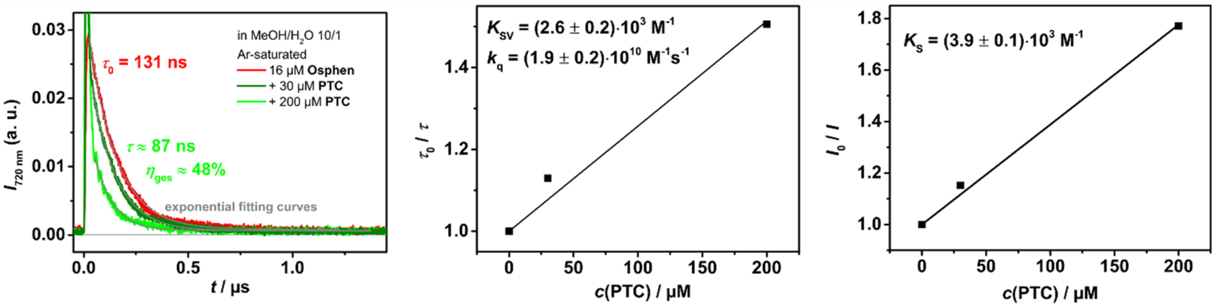

Figure S55 (left) Time-resolved emission (λ_{det} = 720 nm) after laser excitation (λ_{exc} = 532 nm) of Ar-saturated solutions containing **Osphen** without or with **PTC** in MeOH/H_2O 10/1. Corresponding Stern–Volmer plots based on the reduction of the initial emission (middle) or the lifetime (right).

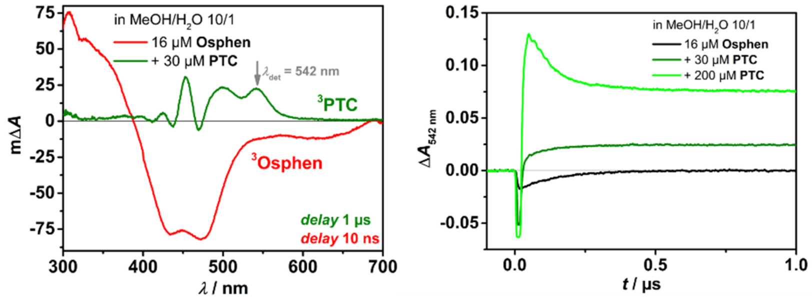

Figure S56 (left) Transient absorption spectra after laser excitation (λ_{exc} = 532 nm) of Ar-saturated solutions containing **Osphen** without (red) or with **PTC** (green) in MeOH/H_2O 10/1. (right) Corresponding time-resolved absorption after laser excitation (λ_{exc} = 532 nm).

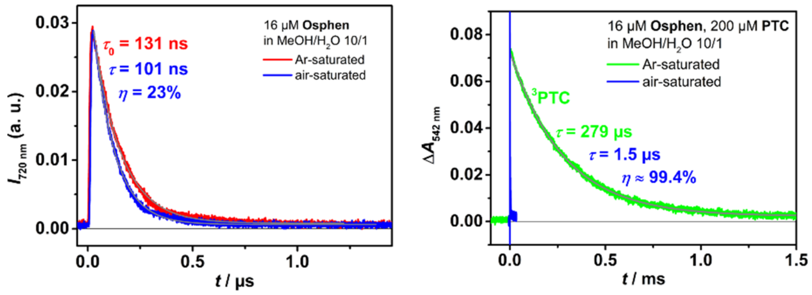

Figure S57 (left) Time-resolved emission (λ_{det} = 720 nm) after laser excitation (λ_{exc} = 532 nm) of Ar-saturated (red) or air-saturated (blue) solutions containing **Osphen** in MeOH/H_2O 10/1. (right) Time-resolved absorption (λ_{det} = 542 nm) after laser excitation (λ_{exc} = 532 nm) of Ar-saturated (green) or air-saturated (blue) solutions containing **Osphen** and **PTC** in MeOH/H_2O 10/1.

S9.6 Photooxygenation of **13**

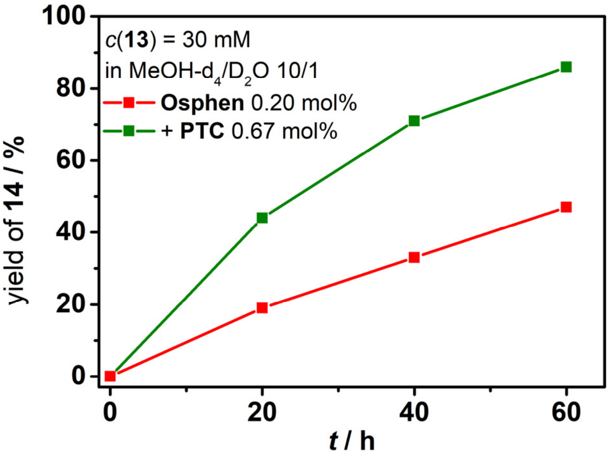

Figure S58 Time-resolved product formation for the photooxygenation (660 nm LED) of **13** with **Osphen** in the absence or in the presence of **PTC** in MeOH-d4/D2O under air.

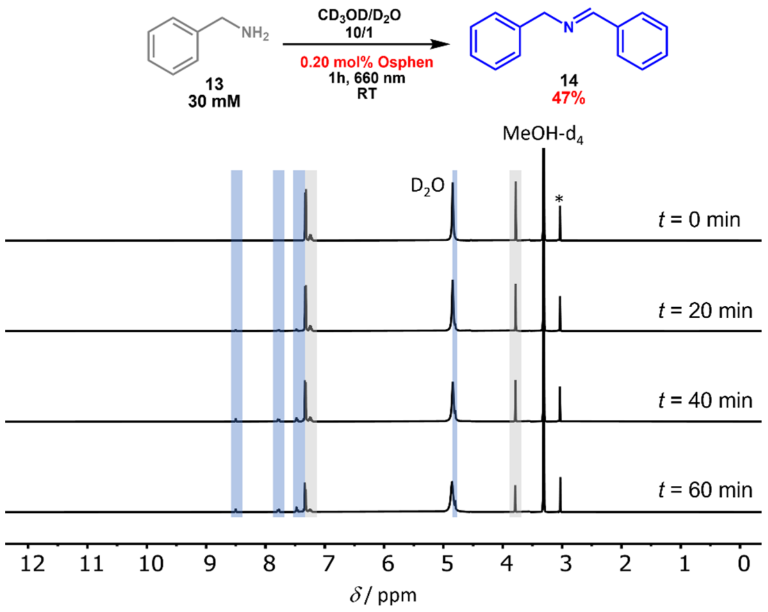

Figure S59 Reaction equation and ^{1}H NMR spectra of the photooxygenation of **13** after different irradiation times (660 nm LED) with **Osphen** in the absence of **PTC** along with signal assignments. The signal marked with an asterisk corresponds to the internal standard dimethyl sulfone with $c \sim 5$ mM.

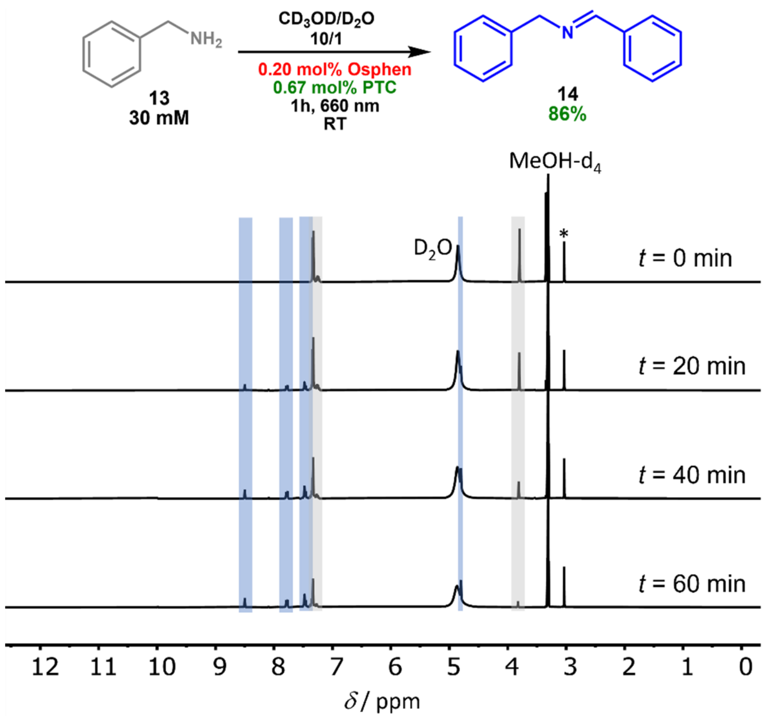

Figure S60 Reaction equation and ^{1}H NMR spectra of the photooxygenation of **13** after different irradiation times (660 nm LED) with **Osphen** in the presence of **PTC** along with signal assignments. The signal marked with an asterisk corresponds to the internal standard dimethyl sulfone with $c \sim 5$ mM. The obtained product chemical shifts are similar to those reported for **14** in chloroform. Signal assignment according to reference [33].

S10 Additional lifetime measurements

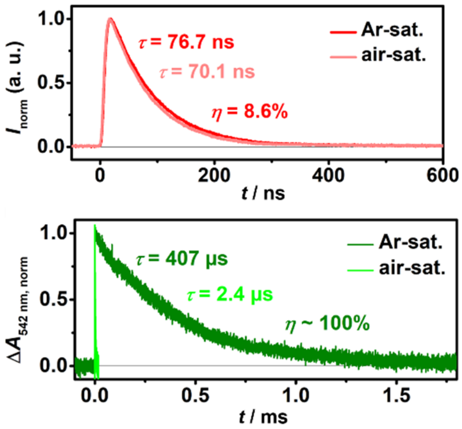

Figure S61 Time-resolved emission of an Ar- or air-saturated aqueous solution (60 mM NaOH) containing c(**Osphen**) = 16 μM (red) and time-resolved absorption of a solution containing additionally c(**PTC**) = 200 μM (green) under similar conditions.

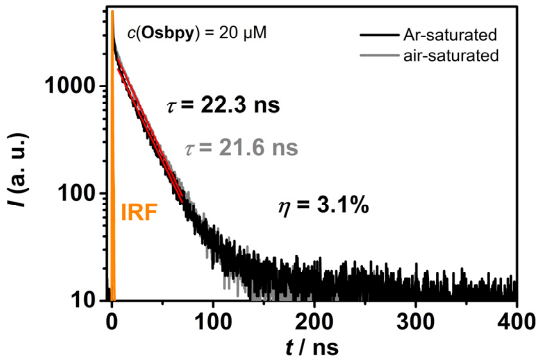

Figure S62 TCSPC measurements of a solution containing 20 µM **Osbpy** in Ar- or air-saturated water upon excitation at 371 nm (detection range approximately 625 – 675 nm). The corresponding lifetime fits are indicated in red.

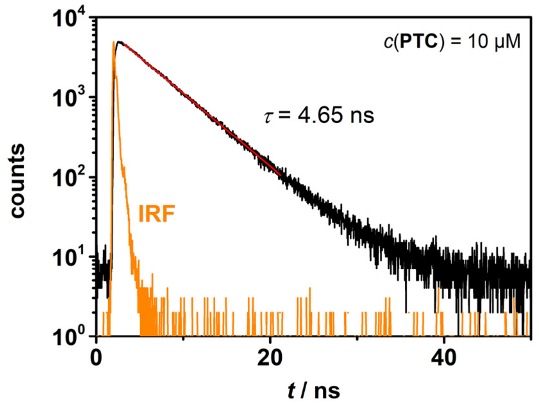

Figure S63 TCSPC measurements of a solution containing 10 µM **PTC** in Ar-saturated water (1 mM NaOH) upon excitation at 446 nm (detection range approximately 575 – 625 nm). The corresponding lifetime fit is indicated in red.

S11 NMR spectra

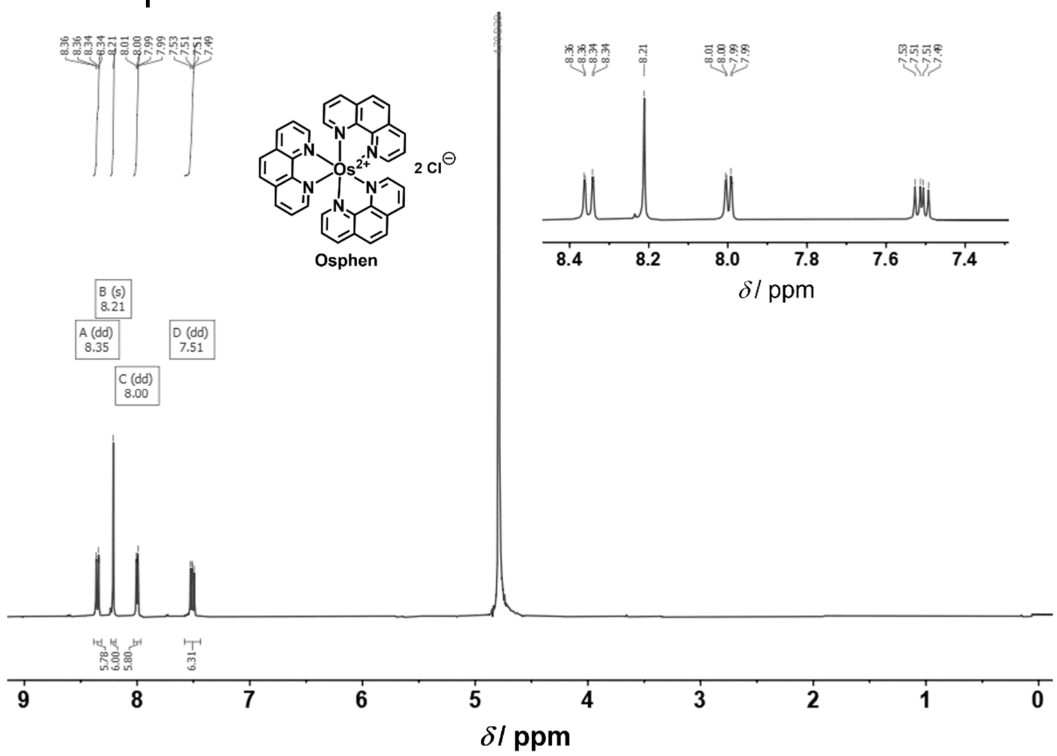

Figure S64 ^{1}H NMR and spectrum of **Osphen** in D_2O.

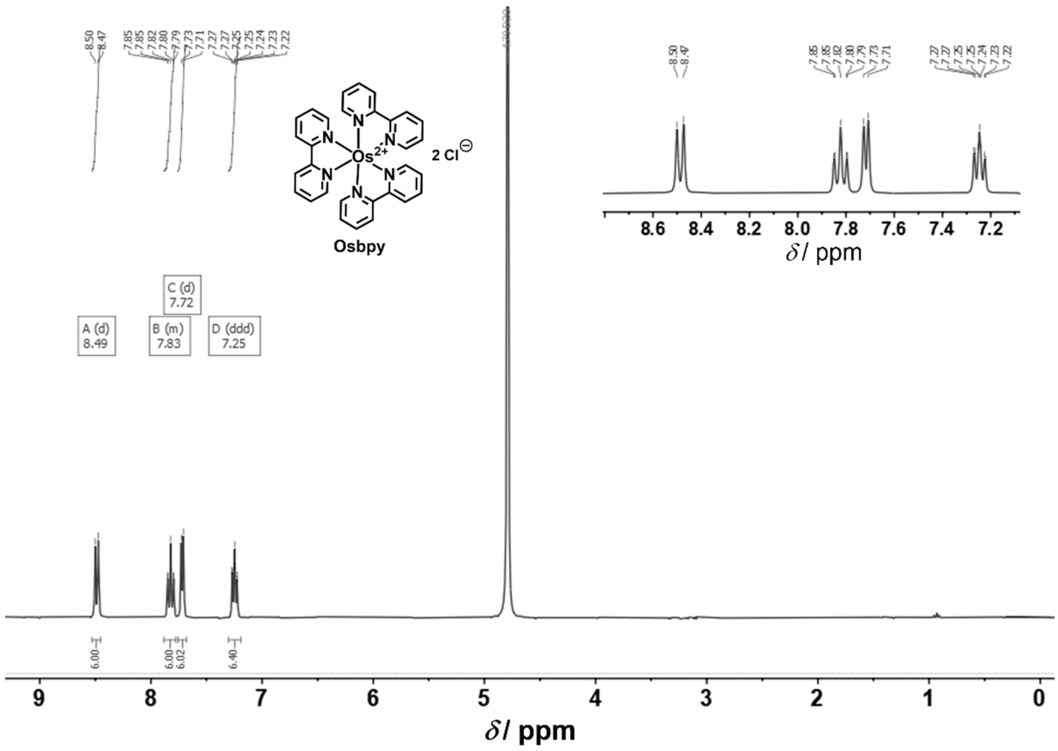

Figure S65 ^{1}H NMR and spectrum of **Osbpy** in D_2O.

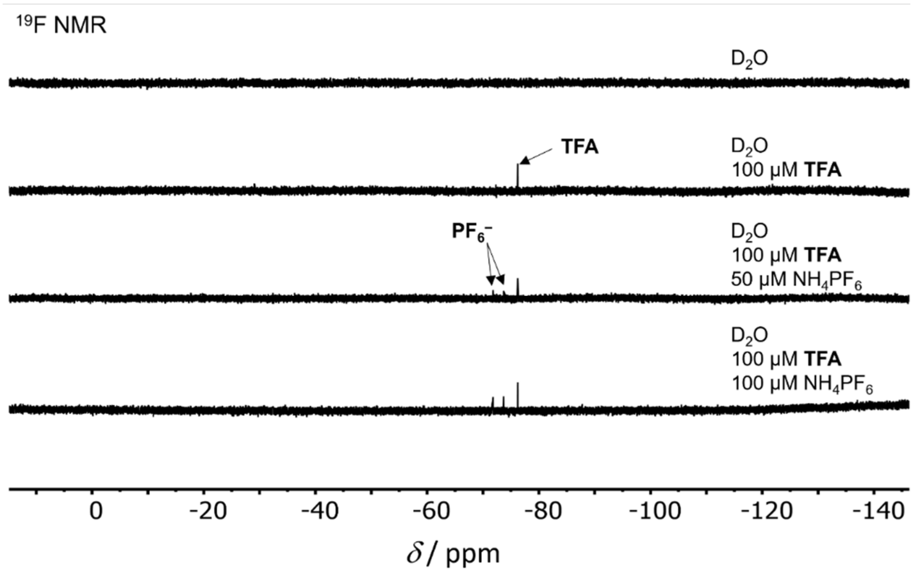

Figure S66 ^{19}F NMR spectra of D_2O solutions containing the additives **TFA** and NH_4PF_6.

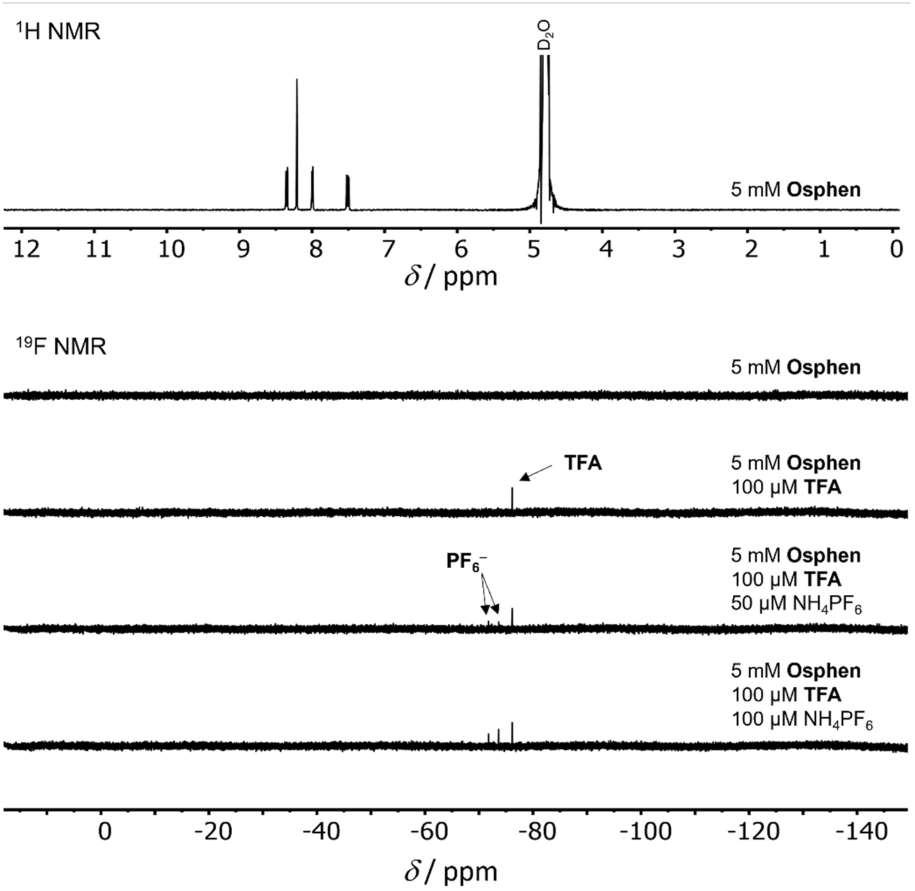

Figure S67 ^{1}H NMR and ^{19}F NMR spectra of solutions containing **Osphen** (chloride salt) with the additives **TFA** and NH_4PF_6 in D_2O.

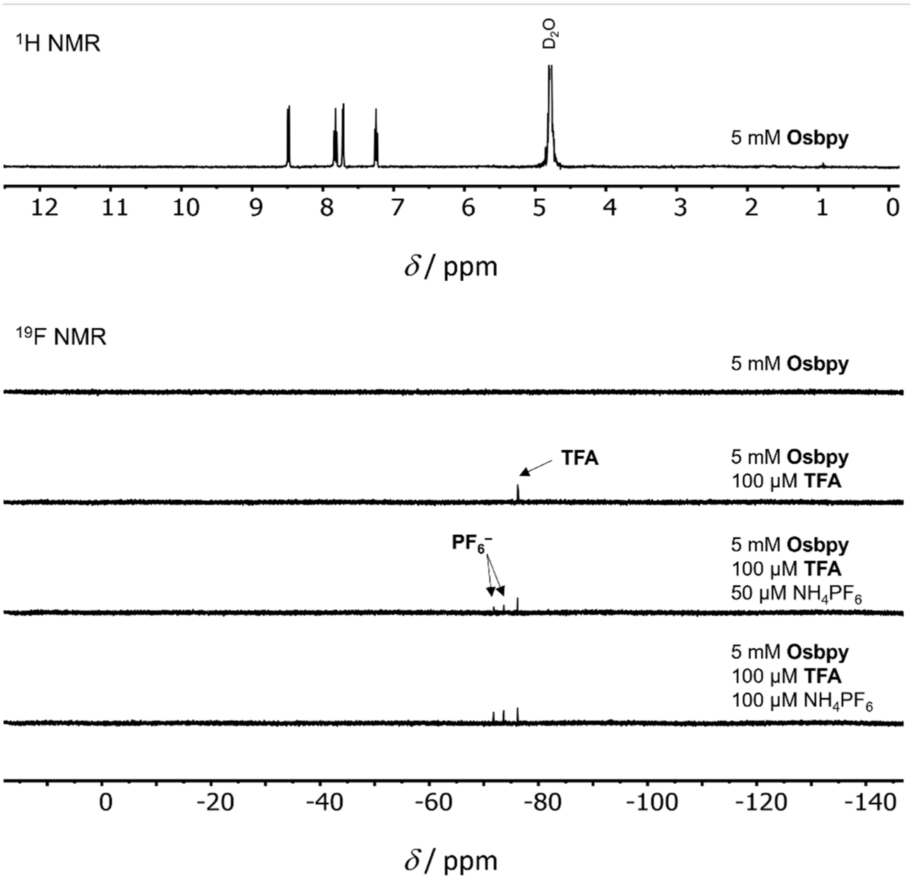

Figure S68 ^{1}H NMR and ^{19}F NMR spectra of solutions containing **Osbpy** (chloride salt) with the additives **TFA** and NH_4PF_6 in D_2O.

For solutions containing 5 mM of the chloride salt of **Osphen** or **Osbpy** (see Figures S67 and S68), the corresponding counterion concentration is 10 mM. Using NH_4PF_6 as internal standard, the detection limit in the NMR experiment was estimated to be ~50 μM (see Figure S66), which corresponds to 0.5% of 10 mM. Since no PF_6^- chemical shifts were observed in the NMR spectra of the chloride salts without additive, the PF_6^- content is estimated to be <0.5%.

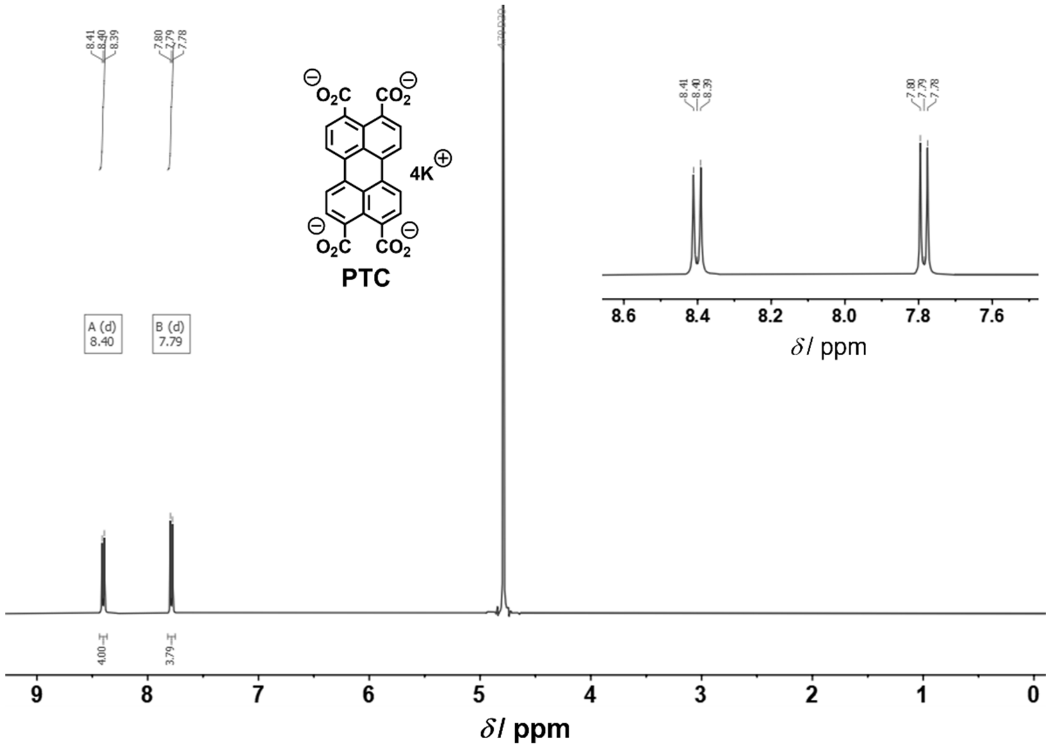

Figure S69 ^{1}H NMR spectrum of **PTC** in D_2O.

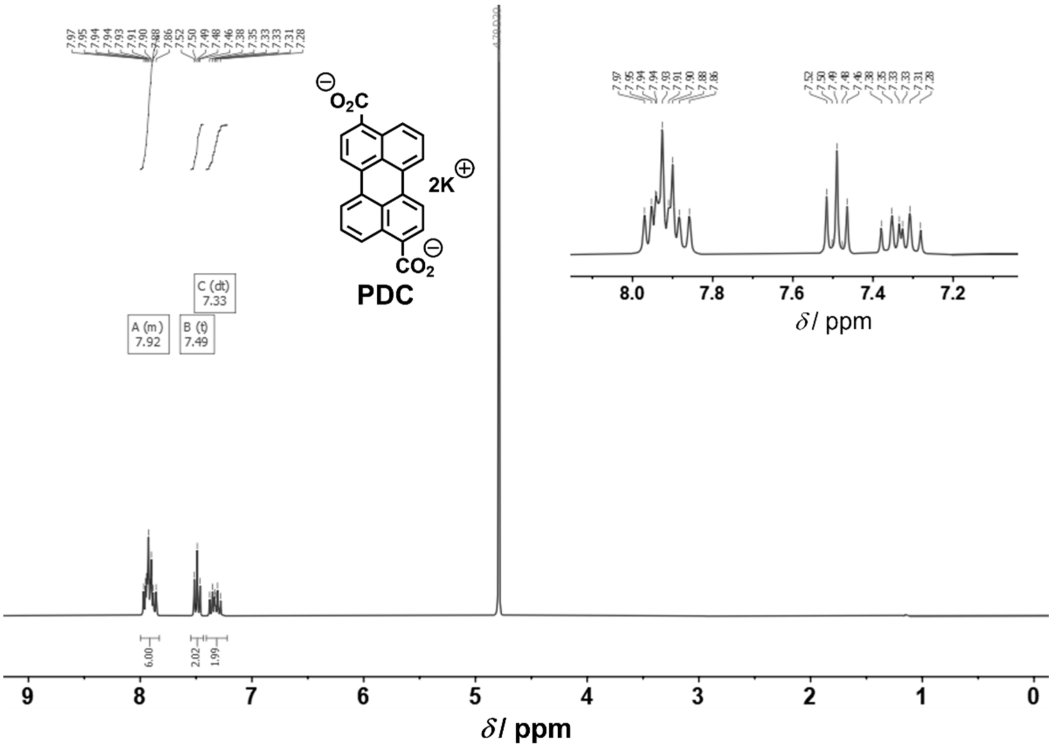

Figure S70 ^{1}H NMR spectrum of **PDC** in D_2O.

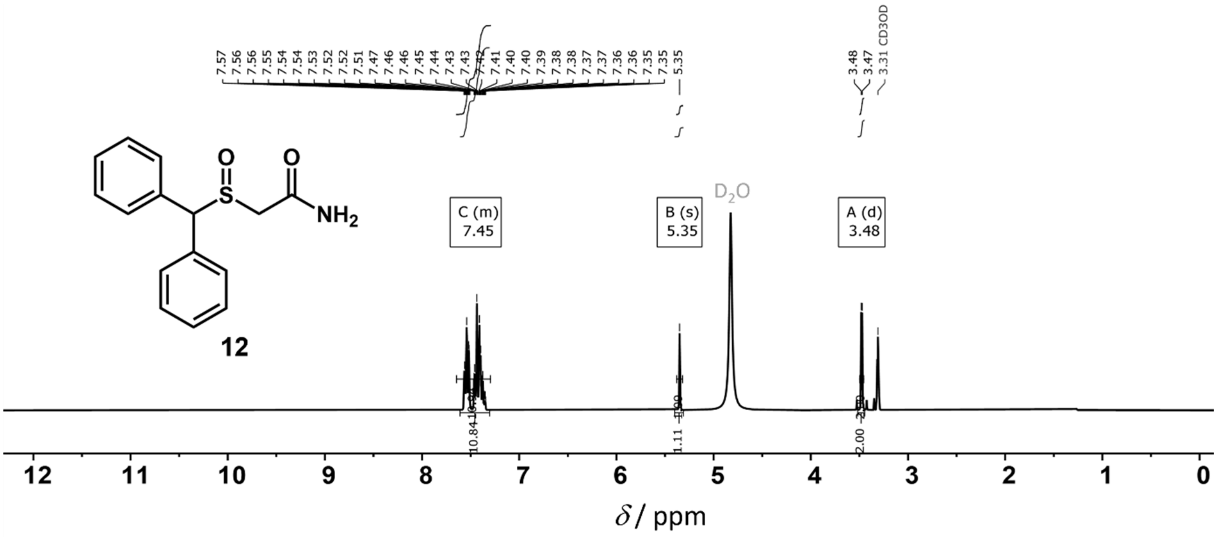

Figure S71 ^{1}H NMR spectrum of modafinil (**12**) in MeOH-d_4/D_2O 10/1.

S12 Literature

[1] C. Müller, T. Pascher, A. Eriksson, P. Chabera, J. Uhlig, *J. Phys. Chem. A* **2022**, *126*, 4087–4099.
[2] F. Jensen, A. Greer, E. L. Clennan, *J. Am. Chem. Soc.* **1998**, *120*, 4439–4449.
[3] G. R. Fulmer, A. J. M. Miller, N. H. Sherden, H. E. Gottlieb, A. Nudelman, B. M. Stoltz, J. E. Bercaw, K. I. Goldberg, *Organometallics* **2010**, *29*, 2176–2179.
[4] F.-F. Zhang, M.-H. Jiang, L.-L. Sun, F. Zheng, L. Dong, V. Shah, W.-B. Shen, Y. Ding, *The Analyst* **2015**, *140*, 280–286.
[5] F. Neese, *WIREs Comput. Mol. Sci.* **2012**, *2*, 73–78.
[6] M. D. Hanwell, D. E. Curtis, D. C. Lonie, T. Vandermeersch, E. Zurek, G. R. Hutchison, *J. Cheminform.* **2012**, *4*, 17.
[7] V. V. Pavlishchuk, A. W. Addison, *Inorg. Chim. Acta* **2000**, *298*, 97–102.
[8] Y. Wei, Y. Li, Z. Li, X. Xu, X. Cao, X. Zhou, C. Yang, *Inorg. Chem.* **2021**, *60*, 19001–19008.
[9] E. C. Constable, P. R. Raithby, D. N. Smit, *Polyhedron* **1989**, *8*, 367–369.
[10] C. Creutz, M. Chou, T. L. Netzel, M. Okumura, N. Sutin, *J. Am. Chem. Soc.* **1980**, *102*, 1309–1319.
[11] Y. Wang, X. Gao, Y. Xiao, Q. Zhao, J. Yang, Y. Yan, J. Huang, *Soft Matter* **2015**, *11*, 2806–2811.
[12] M. Hollauf, P. W. Zach, S. M. Borisov, B. J. Müller, D. Beichel, M. Tscherner, S. Köstler, P. Hartmann, A.-C. Knall, G. Trimmel, *J. Mater. Chem. C* **2017**, *5*, 7535–7545.
[13] C. Magne, S. Streckaite, R. A. Boto, E. Domínguez-Ojeda, M. Gromova, A. Echeverri, F. S. Brigiano, M.-H. Ha-Thi, M. Fanckevičius, V. Jašinskas, A. Quaranta, A. A. Pascal, M. Koepf, D. Casanova, T. Pino, B. Robert, J. Contreras-García, D. Finkelstein-Shapiro, V. Gulbinas, M. J. Llansola-Portoles, *Chem. Sci.* **2024**, *15*, 17831–17842.
[14] J. R. Lakowicz, *Principles of Fluorescence Spectroscopy*, Springer US, Boston, MA, **2006**.
[15] M. Schmitz, M.-S. Bertrams, A. C. Sell, F. Glaser, C. Kerzig, *J. Am. Chem. Soc.* **2024**, *146*, 25799–25812.
[16] P. Thordarson, *Chem. Soc. Rev.* **2011**, *40*, 1305–1323.
[17] D. Brynn Hibbert, P. Thordarson, *Chem. Commun.* **2016**, *52*, 12792–12805.
[18] N. Z. Alqahtani, T. G. Blevins, C. E. McCusker, *J. Phys. Chem. A* **2019**, *123*, 10011–10018.
[19] S. R. Logan, *Trans. Faraday Soc.* **1967**, *63*, 3004–3008.
[20] M. Montalti, A. Credi, L. Prodi, M. T. Gandolfi, *Handbook of Photochemistry*, CRC Press, **2006**.
[21] F. Glaser, C. Kerzig, O. S. Wenger, *Angew. Chem. Int. Ed.* **2020**, *59*, 10266–10284.
[22] I. Kraljić, S. E. Mohsni, *Photochem. Photobiol.* **1978**, *28*, 577–581.
[23] A. Fukatsu, M. Kondo, M. Okamura, M. Yoshida, S. Masaoka, *Sci. Rep.* **2014**, *4*, 5327.
[24] D. Van Der Westhuizen, J. Conradie, K. G. Von Eschwege, *Electroanalysis* **2020**, *32*, 2838–2851.
[25] E. S. Pysh, N. C. Yang, *J. Am. Chem. Soc.* **1963**, *85*, 2124–2130.
[26] P. Wardman, *J. Phys. Chem. Ref. Data* **1989**, *18*, 1637–1755.
[27] Y. A. Ilan, D. Meisel, G. Czapski, *Isr. J. Chem.* **1974**, *12*, 891–895.
[28] A. C. Sell, J. C. Wetzel, M. Schmitz, A. W. Maijenburg, G. Woltersdorf, R. Naumann, C. Kerzig, *Dalton Trans.* **2022**, *51*, 10799–10808.
[29] F. Glaser, O. S. Wenger, *Chem. Sci.* **2023**, *14*, 149–161.
[30] P. Sotero, R. Arce, *J. Photochem. Photobiol. Chem.* **2008**, *199*, 14–22.
[31] N. K. Strizhov, V. V. Poskonin, L. A. Badovskaya, E. P. Kupina, *Russ. J. Org. Chem.* **2002**, *38*, 251–255.
[32] Y.-M. Huang, G.-H. Lu, M.-H. Zong, W.-J. Cui, N. Li, *Green Chem.* **2021**, *23*, 8604–8610.
[33] K. Wu, X.-Y. Liu, P.-W. Cheng, Y.-L. Huang, J. Zheng, M. Xie, W. Lu, D. Li, *J. Am. Chem. Soc.* **2023**, *145*, 18931–18938.
